# Small Extracellular Vesicles in the Pericardium Modulate Macrophage Immunophenotype in Coronary Artery Disease

**DOI:** 10.1016/j.jacbts.2024.05.003

**Published:** 2024-07-03

**Authors:** Soumaya Ben-Aicha, Maryam Anwar, Gemma Vilahur, Fabiana Martino, Panagiotis G. Kyriazis, Natasha de Winter, Prakash P. Punjabi, Gianni D. Angelini, Susanne Sattler, Costanza Emanueli

**Affiliations:** a National Heart and Lung Institute, Imperial College London, London, United Kingdom; b Cardiovascular Program-ICCC, IR-Hospital Santa Creu i Sant Pau, IIB-Sant Pau, Barcelona, Spain; c Hammersmith Hospital, Imperial College Healthcare National Health Service Trust, London, United Kingdom; d Bristol Heart Institute, University of Bristol, Bristol, United Kingdom; e Department of Pharmacology, Otto-Loewi Research Center, Medical University of Graz, Graz, Austria; f Department of Cardiology, Medical University of Graz, Graz, Austria

**Keywords:** coronary artery disease, macrophages, miRNAs, pericardial fluid, small extracellular vesicles

## Abstract

Coronary artery disease (CAD) is a major health issue. This study focused on pericardial macrophages and small extracellular vesicles (sEVs) in CAD. The macrophages in CAD patients showed reduced expression of protective markers and unchanged levels of proinflammatory receptors. Similar changes were observed in buffy-coat–derived macrophages when stimulated with CAD pericardial fluid-derived sEVs. The sEV contained miRNA-6516-5p, which inhibited CD36 and affected macrophage lipid uptake. These findings indicate that sEV-mediated miRNA actions contribute to the decrease in protective pericardial macrophages in CAD.

## Highlights

• Pericardial macrophages in CAD patients exhibit reduced expression of anti-inflammatory and atheroprotective markers, potentially contributing to disease progression.
• Pericardial fluid-derived sEVs from CAD patients alter macrophage phenotype toward a proinflammatory state.
• CAD-PF-sEV macrophages showed lower levels of the miRNA-6516-5p, targeting CD36 and affecting macrophage lipid uptake.

## Visual Abstract

**Figure:**
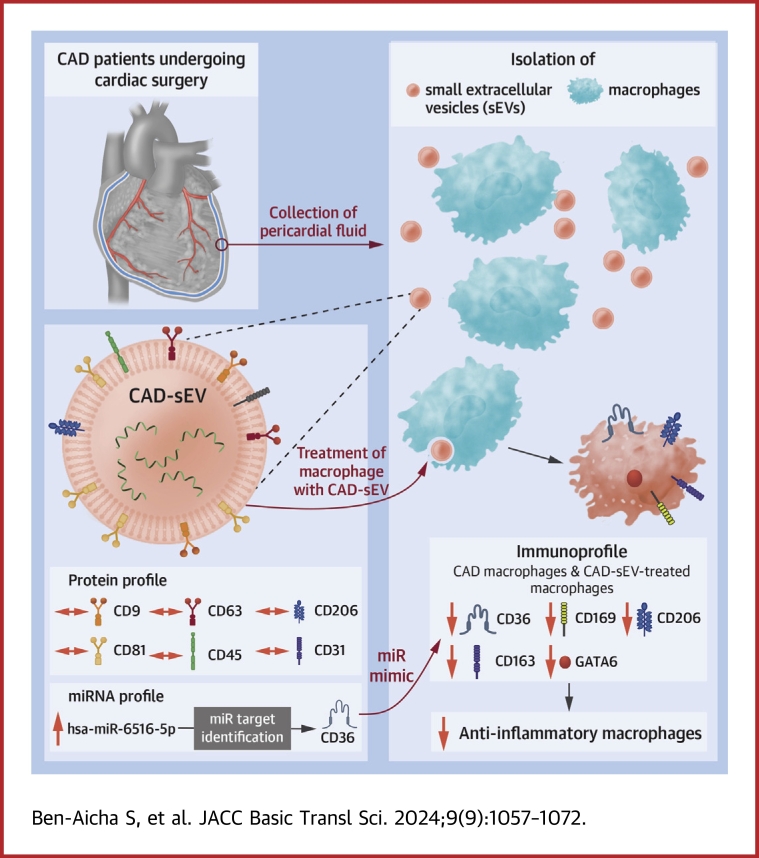

Around 17.9 million people die each year from cardiovascular diseases, 85% of which are caused by heart attack or stroke (World Health Organization). Despite advancements in early diagnosis and treatment, coronary artery disease (CAD) remains one of the most prevalent conditions and the leading cause of ischemic heart disease. CAD is affected by the immune system through complex inflammation and immune-cell responses, where different subtypes of macrophages (Mφ) play central roles.^1^ Mφ induce atherosclerosis progression through cholesterol clearance from the arterial intima through macrophage scavenger receptors, such as CD36.^2^^,^^3^ Yet, conversely, protective roles have also been attributed to Mφ.^3^ Importantly, even if CD36 has different roles in different contexts, CD36 is used as a well-recognized marker for anti-inflammatory Mφ.^4^ Mφ are responsible for immune surveillance and the maintenance of a homeostatic environment by performing crucial roles in clearance such as degradation of erythroid nuclei and apoptotic cells/bodies.^5^ In this anti-inflammatory regard, the Mφ scavenger receptor CD36 has a key protective role by mediating apoptotic cell recognition and phagocytosis.^6^^,^^7^ In fact, high levels of membrane-associated CD36 are associated with reparative and inflammation resolutive Mφ subtypes.^4^^,^^8^ Similarly, CD163 and CD206 surface markers are used to classify macrophages as anti-inflammatory.^4^^,^^8^ Nevertheless, Mφ are highly plastic cells shaped in accordance with their body niche and exposure to pathophysiological conditions.^9^, ^10^, ^11^ Mφ present in the pericardium merit attention because of their close contact with the heart and proximity to the epicardial coronary arteries.^12^ The pericardium is a double-walled sac, which encloses the pericardial cavity and surrounds the heart and the roots of the great vessels. Its inner visceral layer (epicardium) is a serous membrane of mesodermal origin that directly covers the heart. The pericardial sac contains the pericardial fluid (PF), a plasma ultrafiltrate,^13^ and harbors immune cells, including Mφ.^12^, ^13^, ^14^, ^15^ Mφ in the peritoneal cavity are well known to be involved in the monitoring of visceral organs, and the biology of macrophages in different cavities have recently attracted attention.^16^ In particular, single cell RNA-sequencing analyzes have revealed that cavity Mφ are abundantly present in mouse atherosclerotic lesions, suggesting their regulatory role in plaque formation.^15^ Additionally, Deniset et al^17^ recently reported that the PF contains a subpopulation of antifibrotic proregenerative GATA binding protein-6 (GATA-6) + Mφ, able to migrate into the myocardium in response to myocardial infarction (MI) induced by coronary ligation in mice. A later publication, which intended to dispute the intramyocardial migration of the pericardial GATA-6+ Mφ, has otherwise confirmed that these cells accumulated onto the epicardium (hence in possible contact with the epicardial coronary arteries emerge) in response to MI.^12^

We previously reported that the PF of non-CAD patients is enriched in a subset of microRNAs (miRNAs) that are also highly expressed in the heart and thoracic vessels, and have demonstrated that the PF small extracellular vesicles (sEVs) regulate endothelial cell biology and angiogenesis by shuttling the miRNA let-7b-5p.^18^^,^^19^ sEVs are actively secreted nanoparticles (30-200 nm) of endocytic origin. sEVs are surrounded by a lipid bilayer of plasma membrane origin and contain multivarious cargos, including miRNAs and small noncoding single-stranded RNAs, which post-transcriptionally regulate gene expression of their target genes.^20^ sEVs mediate intercellular communication, shuttling their cargos from the cell type of origin and inducing an expressional and functional response in recipient cells.^21^^,^^22^ Moreover, Mφ can be regulated by sEV-miRNAs, affecting inflammation and atherosclerosis.^23^, ^24^, ^25^, ^26^, ^27^

This study is the first to immunoprofile human pericardial Mφ in the setting of CAD. We also investigated the capacity of PF-sEV to shape Mφ phenotype and regulate their lipid uptake via miRNA transfer.

## Methods

### Sample collection and characterization

#### Study design

Human samples were obtained in accordance with the principles of the Declaration of Helsinki 1964 and its later versions. Written informed consent was obtained from all patients. PF cells and sEVs were obtained as leftover from cardiac surgeries performed at the Hammersmith Hospital (Imperial College Healthcare National Health Service [NHS] Trust) under ethical approval number-17/WA/0161-22/WA/0214 and R20003 research protocol. Additional PF-sEV samples were obtained from ARCADIA (REC13/LO/1687 and 17/WA/0161),^28^ which is a prospective observational clinical study in cardiac surgery developed between the Hammersmith Hospital and the Bristol Royal Infirmary (University Hospitals Bristol NHS Foundation Trust). Further details of the patients included are provided in Supplemental Table 1.

#### Pericardial cells and fluid isolation

On the day of surgery, patient PF samples were collected and processed within 40 minutes. To remove any acellular matrix, the samples were centrifugated at 250*g* for 10 minutes. The floating matrix was removed and then a second centrifugation of 300*g* for 10 minutes was performed to isolate the cells. The supernatant was collected for sEV isolation, and cells were cryopreserved at a final temperature of –196 °C (liquid N_2_) for further analysis.

### Macrophage characterization

Pericardial cells were isolated and stained for life/dead discrimination following the supplier's protocol (ZombiNir, Biolegend). Cells were then incubated with FC blocker (564220, BD Biosciences) to avoid unspecific binding, labelled with the appropriate antibody cocktail (Supplemental Table 2) for 50 minutes, and washed twice with commercial flow cytometry buffer (eBioscience, ref. 00-4222-26). Total PF cells were analyzed in Symphony-A3 and DIVA (BD Biosciences) and data analysis was performed using FlowJo 10.8 software.

#### sEV preparation and characterization

sEVs were isolated from PF using size exclusion chromatography (Exospin) following the supplier protocol (Exo-spin03 columns, Cell guidance systems). The sEVs (n = 8 CAD, n = 8 non-CAD) were characterized using the following approaches: The concentration of sEV-sized particles in the PF samples was quantitated by using Nanosight. To this end, a 1:500 dilution was performed in nanoparticle-free phosphate-buffered saline (PBS). sEVs were further stained for tetraspanins analysis on the ExoViewR100 platform (NanoView Biosciences) using ExoView Human Tertraspanins kit (EV-TETRA-C, NanoView Biosciences) following the supplier’s protocol. Enzyme-linked immunosorbent assay (high-density lipoprotein: ab108803; low-density lipoprotein [LDL]: ab190806; Abcam) was performed for sEV lipoprotein content analysis. Concentrations were performed following supplier protocol indications (apolipoprotein [Apo] A1: sEV = 1:100,000; PF = 1:100,000; ApoB: 1:500; sEV: 1:50; PF = 1:200). Additional cell markers were used to infer the sEV cell origin using CD45 (hematopoietic and immune cells), CD206 (anti-inflammatory macrophages), CD31 (endothelial cells, Biolegend), and CD163 and CD86 (additional macrophage markers, Biolegend). For assaying sEV uptake by macrophages, the sEVs were stained using Exoglow following the supplier protocol. Confocal microscopy images of the cells were taken by Leica Stellaris 8 (Leica) and analyzed by Image J Software.^29^

### In vitro macrophage immunoprofiling

#### Preparation of macrophages from buffy coats and exposure to pericardial sEVs

Human-monocyte-derived macrophages were prepared from buffy coats from healthy donors (NHS commercial buffy coats) using gradient separation (Histopaque 1077, Sigma) and adhesion purification. Following Histopaque separation, peripheral blood mononuclear cells were resuspended in RPMI (Life Technologies) with 20% FBS, and monocytes were purified by adherence for 1 hour at 37 °C, 5% CO_2_. The monolayer was washed 3 times with Hank's Balance Salt Solution to remove nonadherent cells. Monocytes were matured for 5 days in RPMI with M-CSF, RANKL (20 ng/mL, PeproTech), and 20% FBS, with medium change every 48 hours. Differentiated cells (Mφ) were harvested and cultured to 12-well plates in a ratio of 1 million cells per well overnight.

Treatment was added in the following groups for 24 hours: untouched Mφ, PBS, lipopolysaccharide (LPS) (1 mg/mL), CAD PF-sEV or non-CAD PF-sEV (5 × 10^7^ particles/mL as previously used^30^). After 24 hours, Mφ were harvested and processed according to the downstream analysis.

#### Macrophage (Mφ) quantitative polymerase chain reaction analysis

Following cell RNA extraction and cDNA preparation (High-Capacity cDNA Archive kit, Applied Biosystems), quantitative polymerase chain reactions (qPCRs) were performed in duplicates using primers for interleukin (IL)-1α, IL-1β, tumor necrosis factor alpha, macrophage mannose receptor 1, scavenger receptor class B type 1, LDL receptor, CD36, and glyceraldehyde-3-phosphate dehydrogenase–endogenous control (Life Technologies) (Supplemental Table 3). The amplification was performed in QuantStudio 6 Flex Real-Time PCR System (Applied Biosystems) in 384-well plates. The amplification curves were analyzed using the Applied Biosystems Sequence Detection System 2.4.1 software. Ct values were obtained and processed by the ddCT method and normalized with the endogenous housekeeping gene.

### Macrophages functional assays

Cells were cultured following the conditions mentioned in the previous text and seeded in 96-well plates for phagocytosis assay (Phagocytosis Assay Kit, Abcam) following the supplier's protocol. Absorbance was measured using Beckman Coulter Paradigm microplate reader (Beckman Coulter). Similarly, fatty acid (FA) uptake was measured by the Fatty Acid Uptake Assay Kit (Abcam). Fluorescence was measured using ClarioStarPlus platform (BMG Labtech), and confocal microscopy images were taken by Leica Stellaris 8 (Leica) and analyzed by Image J Software.^29^

### miRNAs target gene analysis

#### sEV miRNA analyses by small RNA sequencing

The miRNA content of PF sEV samples (n = 4 CAD; n = 4 non-CAD) was determined using the small RNA sequencing Qiagen research platform. To this purpose, sEVs purified from the samples were next shipped to Qiagen for RNA isolation, quality control, miRNA library preparation, sequencing (single-end 75bp at a depth of 12 M), alignment to human genome (hg38), and read count generation.

Raw counts were normalized to counts per million reads mapped. Thereafter, counts per million reads mapped were used to identify miRNAs that were differentially present across groups. miRNAs with log2 fold-change >1 were further studied as lipid metabolism regulators on Mφ. When it comes to FDR, CLC uses Benjamini-Hochberg.^31^ If desired, the reader can read more about the implementation in the CLC Manual.^32^

#### In silico analysis

To identify miRNAs that target lipid metabolism genes in Mφ of relevance in CAD, target prediction tools of miRWalk suite (miRWalk algorithm and databases: TargetScan and miRDB with a binding probability threshold ≥0.95) were used on all miRNAs from section 3.1. Subsequently, KEGG pathways and Gene Ontology terms were found for the predicted targets using DAVID (Database for Annotation, Visualization, and Integrated Discovery) and WebGestalt. Terms with a *P <* 0.05 involving lipid metabolism uptake were considered to further select target candidates. Top target gene was validated by qPCR.

#### miR-6516-5p targets investigation in macrophages

Macrophages were transfected with miR-6516-5p mimic and inhibitor (Life Technologies) with Lipofectamine RNAiMAX Transfection Reagent (Life Technologies) following the supplier recommendations. A miRNA Negative Control #1 was run in parallel. To ensure cell fitness, Mφ were treated 2 hours later with PF-sEV from CAD and non-CAD-sEV. miRNA transfection qPCR analysis was performed following miRCURY LNA RT Kit and miRCURY LNA SYBR Green PCR Kit (Qiagen) protocols (Supplemental Table 3). Ct values were obtained and processed by the ddCT method and normalized with the standardized UniSp6. Luciferase assay was performed in COS7 cells to confirm the interaction between CD36 and miR6516-5p because of their endogenous low levels of CD36.^33^^,^^34^ Culture and assay was performed following the supplier protocol (Origene, Commercially available construct: “CD36 [NM_000072] Human 3' UTR Clone” SC203524).^33^^,^^34^

### Statistics

The Shapiro-Wilks test was used to determine if data were normally distributed. Accordingly, analysis of variance or Kruskal-Wallis test was used to compare >2 groups with a post hoc test for multiple pairwise comparisons (Dunnett's or Dunn's test, respectively). Comparisons between 2 groups were done using the Student’s *t*-test or Mann-Whitney *U* test. A test for outlier data confirmation was performed to identify extreme values for unbiased data analysis. A 2-tailed *P* value <0.05 was considered statistically significant. Data are presented using mean ± SD (normal) and median (Q1, Q3) (non-normal) for continuous variables or count (percentage) for categorical variables. Individual data points are also shown in the figures.

#### Sample size

Using a type I error of 0.05 and 80% power in a 2-sided test, 5 was necessary to determine a difference ≥0.5 U based on previous related studies.^17^ Nevertheless, because of the field's novelty, we performed n = 8 to make our conclusions more robust. The common SD is assumed to be 0.2, and a dropout rate of 0% was anticipated (GRANMO Software). All statistical analyses were performed and graphics using GraphPad Prism version 7.0 software.

## Results

### Pericardial anti-inflammatory macrophage phenotype is reduced in CAD patients

**Figure 1:**
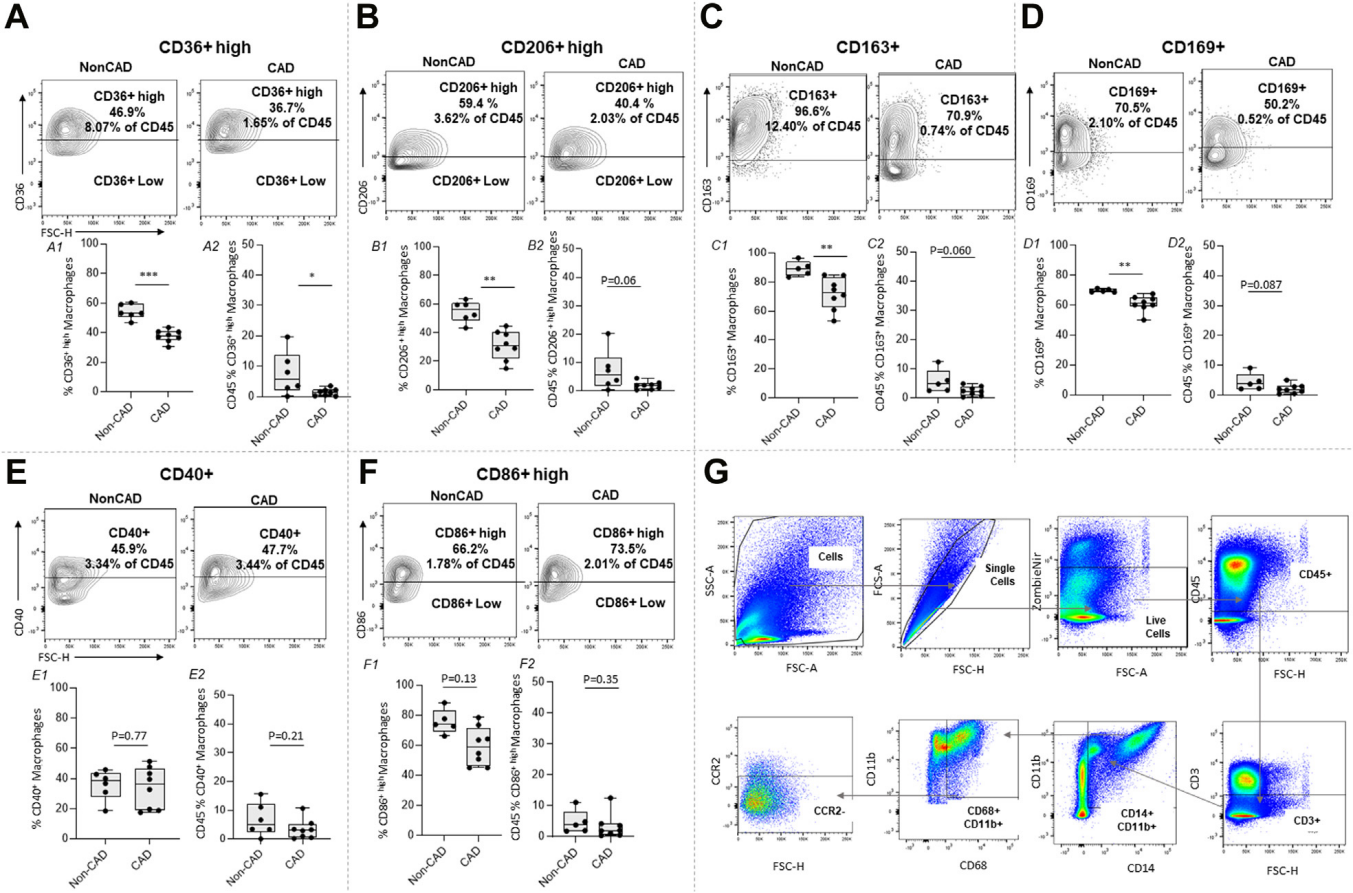
Pericardial Fluid Macrophages Surface Markers Pericardial fluid macrophages surface markers CD36^+/high^ (A), CD206^+/high^ (B), CD163^+^ (C), CD169^+^ (D), CD40^+^ (E) and CD86^+/high^ (F) expressed as Mϕ percentage. Percentage of total CD45% population is also plotted. n = 8. Representative cell population plot is shown for each cell marker. MFI data can be found in Supplemental Table 4. G shows the gating strategy. T-Test (*C1-F1; A2-E2*) or Mann-Whitney (*A1-B1; F2*) test was performed. Data reported as median (IQR). CAD = coronary artery disease; nonCAD = non-coronary artery disease. ∗*P* < 0.05, ∗∗*P* < 0.01, and ∗∗∗*P* < 0.001.

PF macrophages were identified by staining with CD45, CD3, CD14, CD11b, and CD68 (Supplemental Table 2). Profiling of human PF macrophages showed that CAD patients have a reduced population of anti-inflammatory CD36^+/high^ (Figure 1A), CD206^+/high^ (Figure 1B), CD163^+^ (Figure 1C), and CD169^+^ (Figure 1D) cells (CAD vs non-CAD, *P <* 0.05) (Figure 1). No differences were observed for the percentages of cells expressing in costimulatory markers CD86^+^ or CD40^+^ cells (CAD vs non-CAD, *P >* 0.05) (Figures 1E and 1F). No differences were found on median fluorescence intensity (MFI) (Supplemental Table 4A). Similarly, no differences were shown for CD45^+^ percentage macrophage markers except for CD36^+/high^ macrophages, strengthening our data. Despite not being statistically significant, in data expressed as percentage of total CD45, lower levels of anti-inflammatory CD206^+/high^, CD163^+^, and CD169^+^ were reported in CAD vs non-CAD patients.

### PF-sEV purity confirmation and quantification

To confirm the purity of isolated sEV samples, the sEVs were tested for ApoA1 and ApoB content, confirming negligible presence of lipoproteins in comparison to whole PF (Supplemental Figure 1). The sEV concentration was higher in the PF of CAD patients compared to control subjects (Table 1). Total protein concentration was similar in CAD (1.05 ± 0.16 μg/μL) and non-CAD (0.91 ± 0.19 μg/μL) sEV preparations.

**Table 1:** Small Extracellular Vesicle Quantification by Nanosight

	Concentration (particles/mL)	
	CAD (n = 8)	Non-CAD (n = 8)
Mean	72,175 × 10^11^	438,714 × 10^11^
SD	270,111 × 10^11^	136,605 × 10^11^
*P* value	<0.05	

Small extracellular vesicle quantification by Nanosight machine, which allows the particle quantification of the small extracellular vesicles. Groups compared using Student’s *t*-test (significance: *P* < 0.05).

CAD = coronary artery disease.

### PF- sEV profile

Membrane-bound tetraspanins (CD9, CD81, and CD63) are considered the gold-standard markers of extracellularly released sEV. The 3 tetraspanins showed comparable levels across the PF-sEV samples (Supplemental Figure 2). Levels of colocalization of individual tetraspanins on the same sEV were also comparable among CAD and non-CAD samples (CAD 6.4% vs non-CAD 5.8%, *P >* 0.05) (Supplemental Table 5).

When investigating the cell sources of PF-sEV, cell markers indicated a prevalence of immune and hematopoietic cell-derived CD45^+^ sEV (∼50%), including anti-inflammatory macrophage-derived CD206^+^ sEV (∼10%), and the presence of endothelial cell-derived CD31^+^ sEV (∼20%). Additional macrophages markers (CD163 and CD86) showed a limited presence on the sEV (∼5%) (Supplemental Figures 3C to 3E). No differences were detected when comparing the cell origin of PF-sEV across CAD and non-CAD.

### PF- CAD sEVs reduce the anti-inflammatory profile on in vitro Mφ

**Figure 2:**
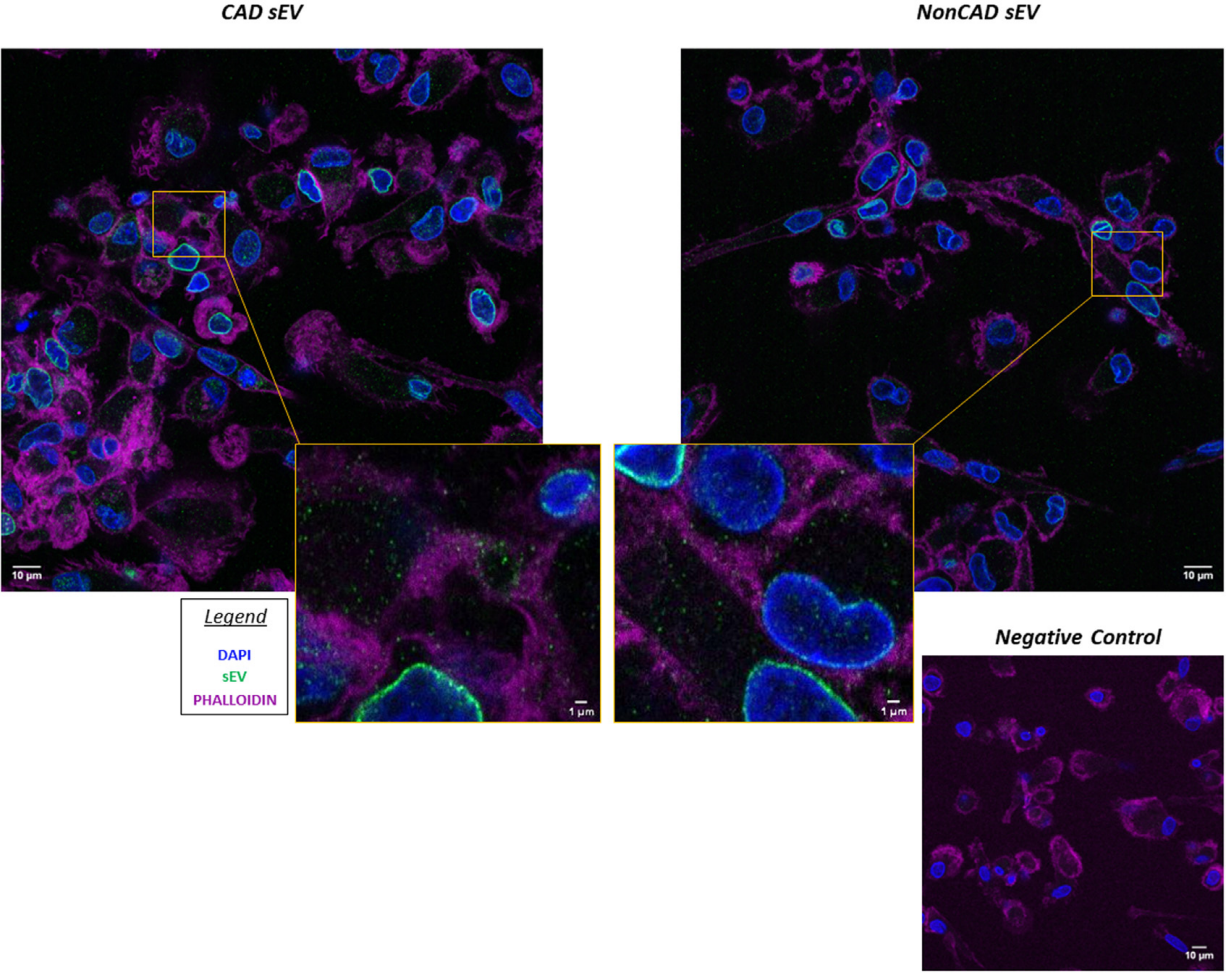
sEV Macrophage Internalization Images showing the sEV macrophage internalization by confocal microscopy. Scale bar: 10 micrometers. Color legend: blue = DAPI; green = sEV; purple = Phalloidin; DAPI = 4’6-diamidino-2=phenylindole; sEV = small extracellular vesicles.

**Figure 3:**
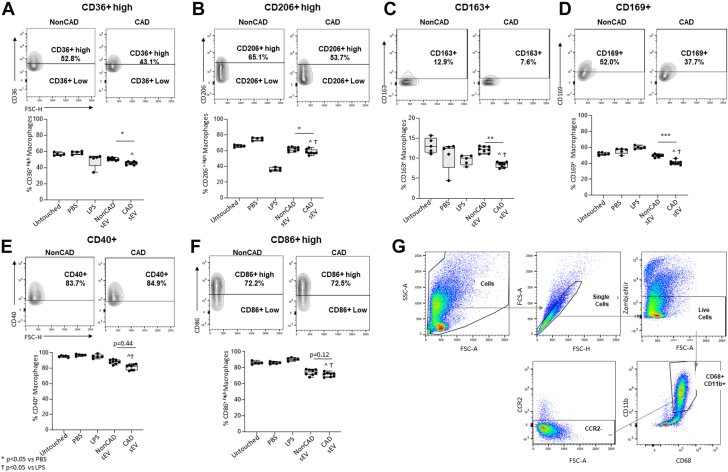
Macrophage Surface Markers Macrophage surface markers CD36^+/high^ (A), CD206^+/high^ (B), CD163^+^ (C), CD169^+^ (D), CD40^+^ (E), and CD86^+/high^ (F) expressed as Mϕ percentage. Percentage of total CD45% population is also plotted. n = 8. Representative cell population plot is shown for each cell marker. MFI data can be found in Supplemental Table 4. Panel G shows the gating strategy. ANOVA with Dunnett’s multiple comparisons (A, B, D, F) or Kruskal-Wallis (C, E) test with Dunn’s multiple comparisons was performed. ^*P* < 0.05 vs PBS; Ϯ*P* < 0.05 vs LPS. Data reported as median (IQR). CAD = coronary artery disease; non-CAD = non-coronary artery disease. ∗*P* < 0.05, ∗∗*P* < 0.01 and ∗∗∗*P* < 0.001.

**Figure 4:**
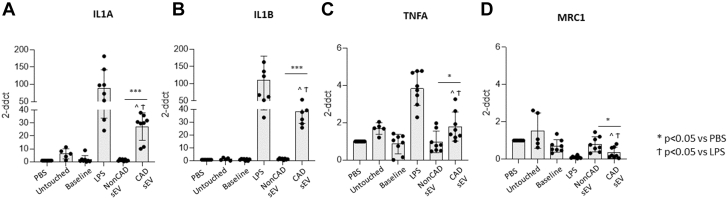
mRNA Levels mRNA levels of pro-inflammatory genes show higher levels in PF CAD-sEV treated macrophages compared to non-CAD and control groups. Opposite results are shown for the anti-inflammatory MRC1. n = 8. Students's *t*-test (B) or Mann-Whitney (A, C, D) test was performed. Data reported as mean ± SD. ∗*P* < 0.05, ∗∗*P* < 0.01, and ∗∗∗*P* < 0.001. CAD = coronary artery disease; non-CAD = noncoronary artery disease.

Confocal microscopy analyses confirmed the ability of macrophages to take up PF-CAD sEV (Figure 2, Video 1). Moreover, Mφs incubated with CAD PF-sEV showed significantly lower levels of anti-inflammatory CD206^+/high^, CD36^+/high^, CD163^+^, and CD169^+^ markers compared with control Mφs (Untouched and PBS) and Mφs incubated with non-CAD PF-sEV (Figures 3A to 3D, Supplemental Figure 4). No differences were observed in the percentage of cells expressing CD86^+^ or CD40^+^ costimulatory markers. However, MFI reported higher fluorescence of anti-inflammatory CD36 (MFI) in Mφs incubated with non-CAD vs CAD, supporting our downstream analyses (Supplemental Table 4B). The flow cytometry profile was in line with transcriptional data. As such, Mφs incubated with CAD PF-sEV expressed higher levels of IL1A, IL1B, and tumor necrosis factor alpha and lower levels of macrophage mannose receptor 1 content vs non-CAD, baseline, and control groups (untouched, PBS) (Figure 4).

### PF-CAD sEVs reduce the GATA6^+^ MΦ population

**Figure 5:**
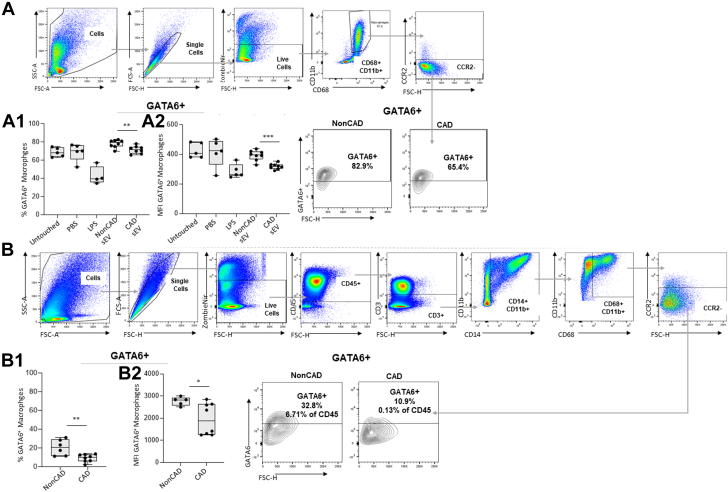
GATA6 Macrophage Population (A) GATA binding protein 6 (GATA6)+, CC-chemokine receptor 2 (CCR2) population is decreased in macrophages incubated with pericardial fluid CAD compared with pericardial fluid non-CAD-sEV and control subjects. (B) The GATA6+, CCR2-macrophage population is decreased in pericardial fluid macrophages from CAD patients compared with non-CAD. n = 8. Student’s *t*-test (A1, A2, B1) or Mann-Whitney *U* test (B2) was performed. Data reported as median (Q1, Q3). ∗*P* < 0.05, ∗∗*P* < 0.01 and ∗∗∗*P* < 0.001. Abbreviations as in Figure 2.

CAD PF-sEV reduced the expression of GATA-6 in CC-chemokine receptor 2 macrophages prepared from buffy coats (Figure 5A).^35^ In line with the in vitro analysis, a lower percentage of GATA6^+^ Mφs was found in the pericardial cells from CAD compared with non-CAD patients (Figure 5B). A deeper immunophenotyping of the GATA-6^+^ Mφs subpopulation revealed that incubation with CAD sEVs also decreased the percentage of the population of Mφs expressing anti-inflammatory CD206^+/high^ and CD36^+/high^ (Supplemental Figure 5). No difference was found for the proinflammatory CD40 and CD86^+high^ populations. Interestingly, our GATA-6^+^ population did not coexpress CD169 (Supplemental Figure 5). Analysis of patients’ pericardial fluid confirmed a lower percentage of CD36^+/high^ population in the GATA-6^+^ Mφs population from CAD patients vs non-CAD (Supplemental Figure 6). Expression levels (MFI) of anti-inflammatory surface marker CD36 was higher in Mφs from non-CAD vs CAD patients.

### Regulation of scavenger receptors and lipid uptake in cultured macrophages stimulated with PF-sEV

**Figure 6:**
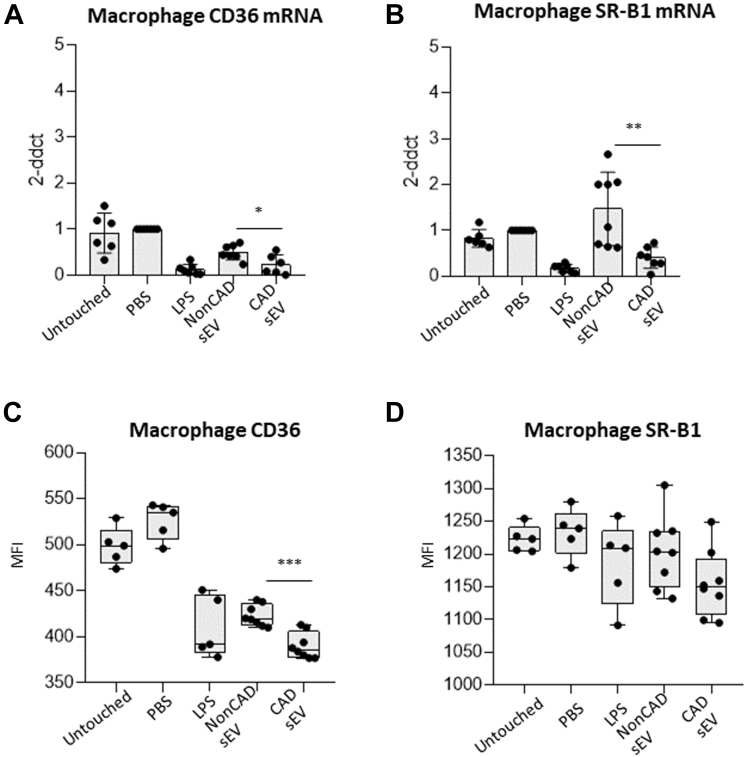
Scavenger Receptor CD36 and SR-B1 Levels (A) Scavenger receptor CD36 is down-regulated at the mRNA and surface protein levels in macrophages when exposed to CAD vs non-CAD sEVs. (B) Scavenger receptor SR-B1 is down-regulated at the mRNA but not at the surface protein levels by flow cytometry in macrophages when exposed to pericardial fluid CAD vs non-CAD sEV. n = 5-8. Student’s *t*-test (A to D) was performed. Data reported as median (Q1, Q3). ∗*P* < 0.05, ∗∗*P* < 0.01, and ∗∗∗*P* < 0.001. MFI = median fluorescence intensity; other abbreviations as in Figures 2 and 3.

To test whether PF-sEV possess the capacity of Mφ to control lipid metabolism, we analyzed the expression of scavenger receptors CD36 and SRB1 (SCRAB1 gene). The transcript levels of CD36 were significantly down-regulated in Mφ treated with CAD PF- sEV (*P <* 0.0001 vs PBS/untouched; *P <* 0.020 vs non-CAD PF-sEV) (Figure 6A). Similarly, scavenger receptor class B type 1 expression was decreased in Mφ incubated with CAD PF-sEV (*P <* 0.003 vs PBS; *P <* 0.004 vs non-CAD PF-sEV) (Figure 6B). LDL receptor transcript levels were increased in cells incubated with CAD PF-sEV when compared with PBS-treated (*P <* 0.04) and untouched (*P <* 0.002) cell controls, but not when compared with non-CAD PF-sEV (Supplemental Figure 7A). No differences were detected for ATP-binding cassette transporter A1 nor G1 (Supplemental Figures 7B and 7C). CD36 protein levels were also decreased in Mφ treated with CAD-PF-sEV when compared with all other groups (*P <* 0.04 vs non-CAD PF-sEV; *P <* 0.0001 vs PBS and untouched) (Figure 6C).

### miR6516-5p target genes on macrophages

**Figure 7:**
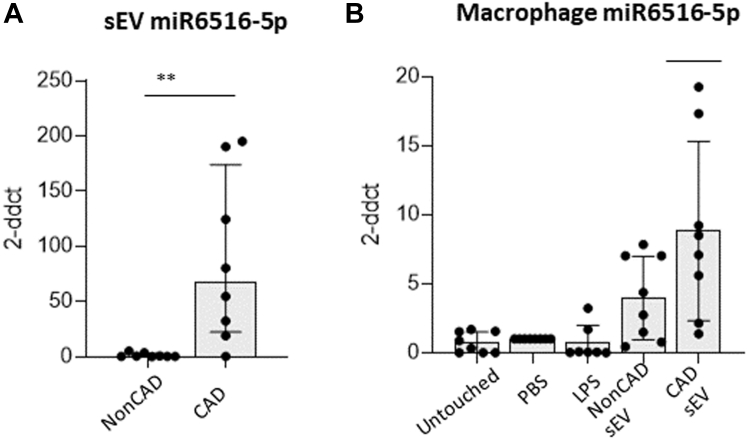
miR6516-5p Levels (A) sEV derived from CAD pericardial fluid show higher levels of miR-6516-5p compared with non-CAD. (B) Similarly, higher levels of miR-6516-5p are observed when macrophages are incubated with pericardial fluid CAD-sEV when compared with control and non-CAD control groups. n = 6-8. Student’s *t*-test (B) or Mann-Whitney *U* test (A) was performed. Data reported as mean ± SD. ∗*P* < 0.05, ∗∗*P* < 0.01, and ∗∗∗*P* < 0.001. Abbreviations as in Figures 2 and 3.

**Figure 8:**
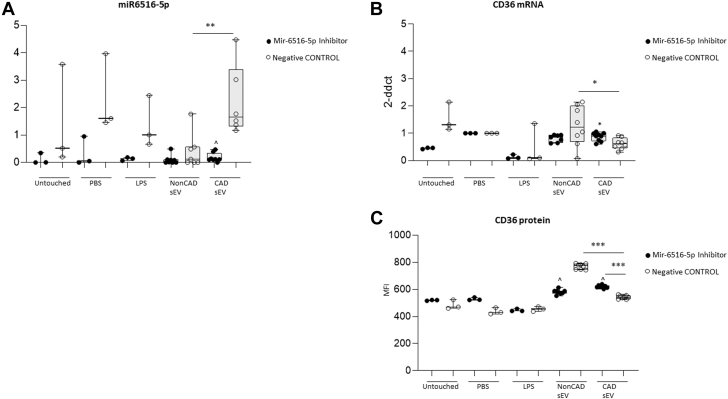
miR-6516-5p Inhibitor miR-6516-5p inhibitor approach. miR6516-5p expression levels by qPCR (A) and CD36-mRNA expression and protein levels (B and C). Student's *t*-test (B, C) or Mann-Whitney (A) test was performed. ^*P* < 0.05 vs negative control. n = 3-8. Data reported as median (IQR). ∗*P* < 0.05, ∗∗*P* < 0.01, and ∗∗∗*P* < 0.001. CAD = coronary artery disease; non-CAD = noncoronary artery disease.

**Figure 9:**
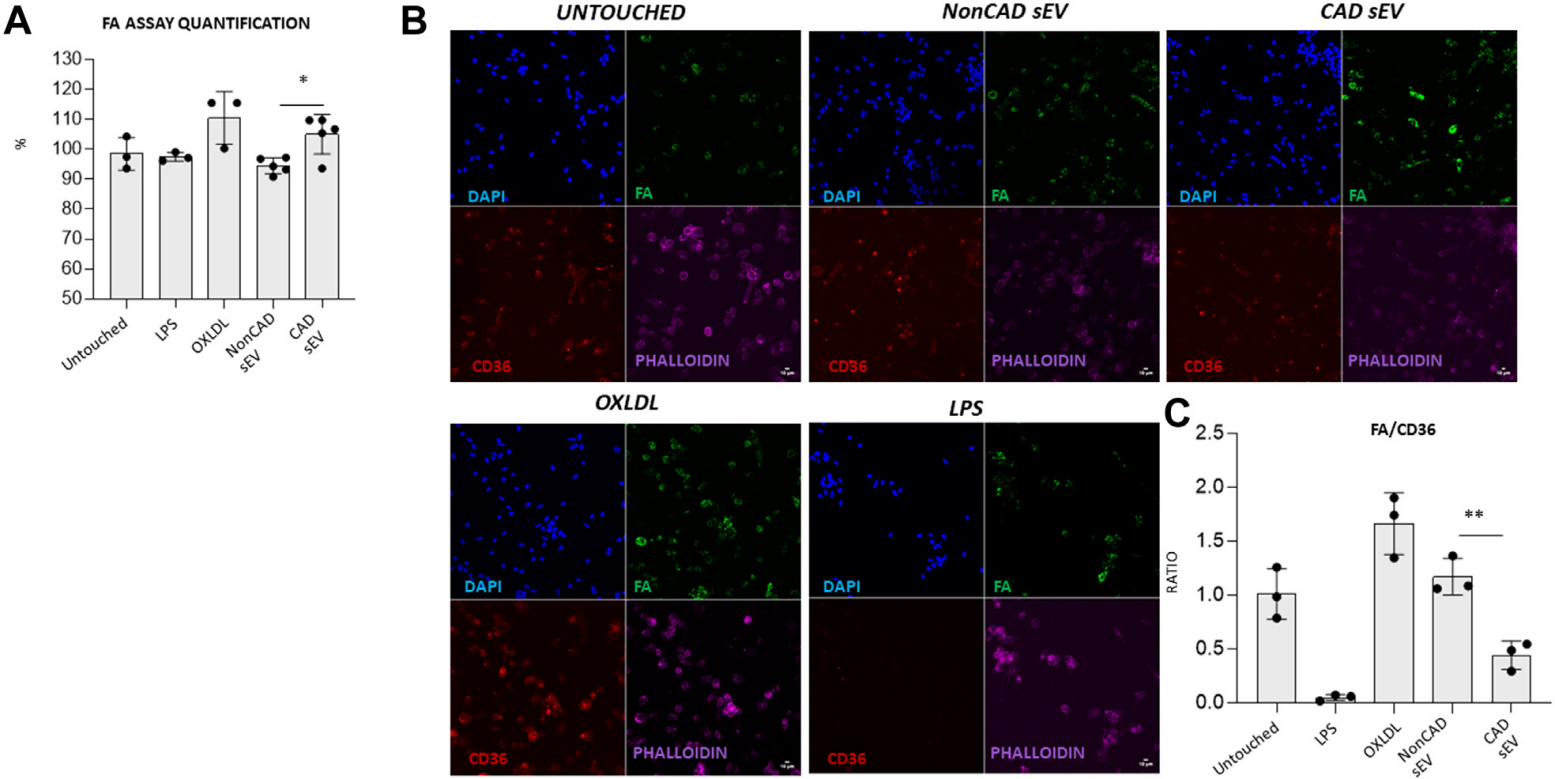
Fatty Acid Uptake Fatty acid (FA) uptake is increased in CAD pericardial fluid macrophages compared with non-CAD. n = 3. On the contrary, the FA uptake to CD36 was decreased. Student’s *t*-test (A and C) was performed. ^*P* < 0.05 vs negative control n = 3-5. Data reported as mean ± SD. ∗*P* < 0.05, ∗∗*P* < 0.01, and ∗∗∗*P* < 0.001. Abbreviations as in Figures 2 and 3.

To identify the miRNAs potentially responsible for the observed Mφ regulation, we analyzed the miRNA-data sets obtained from our performed small RNA-sequencing of PF-sEVs. A total of 1,344 miRNAs were detected, from which 223 reported log_2_ fold-change >1 (non-CAD vs CAD) (Supplemental Figure 8). Using bioinformatics prediction tools (mirWalk+miRDB), we next attributed CD36 as a potential target gene (in line with data in section 6), resulting in the prediction of 5 miRNAs able to target the CD36 messenger RNAs (mRNAs) for repression. Of the top emerging miRNA candidates (Supplemental Tables 6 and 7). we selected hsa-miR-6516-5p because of its high fold change between CAD and non-CAD sEVs and because it had not previously been implicated in lipid metabolism (hsa-miR-3934-3p was discarded because of lack of genomic validation and confidence in the literature). Using qPCR, we next confirmed higher levels of miR-6516-5p in CAD sEVs vs non-CAD sEVs (Figure 7A). Additionally, increased levels of miR-6516-5p were shown on CAD-sEV-treated Mφ when compared with non-CAD-sEV-treated cells (Figure 7B). MiR-6516-5p inhibitor and mimic transfection confirmed the functional capacity of endogenous hsa-miR-6516-5p to target CD36 (Figure 8, Supplemental Figure 9). MiR-6516-5p levels where nulled in the presence of the miRNA inhibitor or enhanced by mimic (Figure 8, Supplemental Figure 9). Cells transfected with the inhibitor or mimic did not show differences on CD36 transcript levels when comparing CAD sEV-treated vs non-CAD sEV-treated macrophages (Figure 8B). However, in both analyses, negative control transfection retrieved basal response, showing higher levels of CD36 transcript levels on non-CAD sEV-incubated cells (Figure 8 and Supplemental Figure 9). Next, flow cytometry confirmed that membrane changes in the CD36 protein levels followed with the transcriptional response to miR-6516-5p inhibition (Figure 8C). In line with the in vitro data, decreased CD36 levels were found in pericardial Mφs from CAD patients compared with non-CAD patients (Supplemental Figure 10). To further confirm the interaction between CD36 and miRA-6516-5p, luciferase assay was performed. Cells transfected with the CD36 plasmid plus mir6516-5p showed lower levels of the luciferase reported when compared with negative control groups (*P <* 0.05) (Supplemental Figure 11). At the functional level, Mφ FA uptake analysis showed that cells incubated with CAD-sEV vs non-CAD-sEV had higher FA uptake (Figures 9A and 9B). Mechanistically, the FA uptake ratio by CD36 (FA/CD36) was lower in CAD vs non-CAD. As expected, Mφ incubated with control oxidated LDL showed high levels of CD36 and associated FA uptake (Figure 9C).^3^ Overall, the data support an anti-inflammatory role of CD36 on Mφ other than the widely described proinflammatory character in the atherosclerotic plaque. Mφ phagocytosis levels remained at baseline regardless of the sEV stimuli received (Supplemental Figure 12).

## Discussion

This study is the first to report changes in pericardial macrophage phenotypes and sEVs in the setting of coronary disease. We have demonstrated the following: 1) the anti-inflammatory macrophage populations were depauperated in the pericardium of CAD patients; 2) PF-sEV formed in a CAD environment have detrimental effects on Mφ by reducing their anti-inflammatory Mφ population in vitro; and 3) the expression level of miR-6516-5p could be targeted therapeutically to regulate the expression of anti-inflammatory CD36 in pericardial macrophages.

These findings could inspire a new line of research and strategies for therapeutic targeting within the pericardial niche. New and better low-invasive therapies are needed to address CAD. At present, treatment efforts are focused on limiting the lipid levels in circulation; however, local inflammation remains crucial in plaque development and needs to be tackled.

### PF sEV Mφ immunomodulatory capacity

Besides its widely recognized properties of biomechanical protection and lubrication,^13^ we and others have reported that the pericardium and its fluid contain cytokines, growth factors, and sEV-contained miRNAs.^18^^,^^35^, ^36^, ^37^ Here, we have characterized the cellular origin of the sEVs present in the pericardial fluid and identified that sEVs are mostly secreted from CD45+ immune cells. However, no direct correlation for cell percentage and sEV marker levels was found. This fact is in line with the biogenesis of sEV and sEV cell-to-cell exchange.^38^ Of relevance, our findings provide evidence that the pericardium is an immune-surveillance reservoir, where macrophages are potentially modulated by the local sEV. In corroboration, we report that the CAD environment shapes Mφ toward a less anti-inflammatory profile, even if it does not increase the activation inflammatory markers. This was unexpected, considering that Mφ are cells commonly recognized for their early response during the inflammatory process and possess a crucial proatherogenic role in the early stages of plaque formation in the vascular intima.^1^^,^^39^^,^^40^ The deeper Mφs immunophenotyping analyzing the Mφ markers as percentage of total CD45^+^ further supported CD36 as the only marker with statistically significant changes in this subanalysis.

CD36 is a complex pleiotropic receptor, with the capacity to take on different functions, depending on its cellular location and post-translational chemical modifications.^3^ At the functional level in atherosclerosis, it is known that the interactions of CD36 with thrombospondins or with oxidated LDL trigger pro-inflammatory responses,^41^ promoting plaque development.^42^, ^43^, ^44^ As a pattern recognition receptor, CD36 is also involved in the clearance of cell debris and phagocytosis,^6^^,^^7^ which are essential functions exerted by macrophages for tissue repair and inflammation resolution, especially in a cavity environment such as the pericardium.^3^^,^^5^^,^^13^^,^^16^ Anti-inflammatory Mφs are characterized by higher clearance and inflammation resolution capacity, together with other immune markers assessed in this study, such as CD206, CD163,^6^ and CD169,^3^^,^^4^^,^^8^ all decreased in CAD patients’ pericardial Mφ and buffy coat-derived Mφ exposed to PF-CAD-sEV in vitro. Coexisting with the anti-inflammatory pericardial Mφ populations, we detected activated CD40^+^ or CD86^+high^ populations, which were comparable between the 2 groups of patients and in the cultured cells exposed in vitro to sEVs from either patients’ group. Additionally, the Mφ immunophenotype was reflected in their Mφ-cytokine mRNA levels, and cells exposed to PF-CAD-sEV released higher levels of pro-inflammatory cytokines compared with control subjects.

A subtype of Mφs present in the pericardium was described to exert anti-inflammatory and reparative function.^4^^,^^16^^,^^17^^,^^36^ We introduce here a plausible modulator to the findings of Deniset et al^17^ and Zindel et al,^45^ who recently showed that a population of cardioprotective Mφs in the pericardium expresses the transcription factor GATA6 and travel into the myocardium following cardiac injury to prevent adverse fibrosis. However, this concept is under debate and needs further evidence.^12^ In a parallel study, Buechler et al^46^ (2019) reveal a role for stromal cells in controlling the homeostasis of pericardial Mφ. Comparably, the present study confirms the importance of PF Mφs in cardiovascular diseases because the protective PF-GATA6^+^ Mφ subpopulation is decreased in inflammatory conditions such as CAD. We show that this decrease is modulated by the sEV found in the pericardial fluid. Unlike one of the findings of Fatehi Hassanabad et al,^47^ differences in the CD163^+^ Mφ population were observed in our study. It is important to note that the populations under investigation were distinct. Deniset et al focused on a cohort of patients experiencing acute coronary syndrome, whereas our study cohort exclusively comprised patients with chronic stable angina. Additionally, our control group consisted solely of non-CAD patients undergoing mitral valve replacement/repair procedures, whereas their control group encompassed a mixture of individuals with aortic stenosis and mitral regurgitation. These interesting differences highlight the importance of the field and open avenues toward advancement of personalized medicine in CAD. Additionally, and supporting our findings, few studies have reported a potential regulation of cardiovascular diseases from the surrounding tissues and spaces, including adipose tissue and pericardial cavity,^3^^,^^48^ and validated these findings with in vitro studies.^49^

### PF-sEV-miRNA functional cargo

We found that mRNA and surface protein levels of CD36 were decreased on CAD PF-sEV-exposed Mφ potentially regulated by sEV-miRNA cargo. We identified miR-6516-5p carried by PF-sEV as a modulator of the lipid metabolism profile by targeting CD36 in our Mφ exposed to PF-sEV. Little information exists regarding the role of miR-6516-5p in the human body; herein, we provide the first evidence of the implication of miR-6516-5p in cardiovascular medicine. A recent study demonstrated that the long-noncoding RNA-SNHG20 sponges miR-6516-5p, and subsequently increases tumor cell proliferation and invasion in prostate cancer.^50^ These data suggest a potential role for miR-6516-5p in disease development. Additionally, higher levels of miR-6516 were detected in old mouse brains compared with their young counterparts, supporting its potential role as an ageing biomarker.^51^ We have now demonstrated by RNAseq that miR-6516-5p levels are higher in PF-CAD-sEV, an observation further validated in human PF-sEV by qPCR. Moreover, miR6516-5p inhibitor and mimic transfection experiments proved specificity suppressing the scavenger receptor CD36 in Mφ. Additionally, specificity was confirmed by our luciferase assay results. Collectively, these findings highlight the significance of sEVs and their miR6516-5p cargo. Nevertheless, additional investigations are needed to elucidate the potential multifactorial stimuli influencing the observed anti-inflammatory profile in macrophages.

To better understand the functional implication of CD36 in Mφ, the distinct metabolic profiles of Mφ-subtypes have to be considered. Proinflammatory Mφs generate ATP primarily from enhanced glycolysis and pentose phosphate pathway, glutaminolysis, inducible nitric oxide synthase-mediated arginine metabolism, and lipogenesis.^52^^,^^53^ Collectively, this metabolic program supports the main microbicidal and tumoricidal roles of proinflammatory macrophages.^54^ However, such macrophages can also cause host tissue damage. In contrast, anti-inflammatory macrophages generate ATP primarily by enhanced oxidative phosphorylation and by β-oxidation of FAs, and biomaterials from proline and polyamine synthesis via arginase-dependent metabolism. We found that Mφ incubated with CAD-sEV take up higher levels of FAs; however, they also show lower levels of CD36/FA ratio, indicating that FA uptake can occur other than via CD36. In fact, extracellular FAs can be recognized and/or taken up by a range of cell membrane G protein-coupled receptors, including but not limited to the scavenger receptor CD36, FA-binding proteins, or members of the FA transport protein family. Of relevance for the disease pathophysiology, once inside the cell, FAs can become fuel for β-oxidation, substrates for complex lipid synthesis (eg, phospholipids for cell membranes), substrates for lipid mediator synthesis, or signals for nuclear receptors that control expression of genes related to metabolism and inflammation amongst other processes.^55^^,^^56^ Taken together, our results provide evidence of the presence of an anti-inflammatory Mφ reservoir in the pericardium, and its alteration in the setting of CAD, with potential consequences for the vasculature.^3^^,^^48^

Our findings have clinical implications because the intact pericardial cavity during MI is significant. However, cardiac surgeries often remove pericardial fluid and tissue without reclosure, potentially removing beneficial Mφ and hindering the contribution of PF-sEV to immune cell remodeling and function. More broadly, these data highlight that targeting the pericardial space is a potential immune-modulatory therapeutic avenue for many cardiac-related pathologies and interventions.

### Study limitations

This study was performed using human material and clinical samples with translational impact and new and relevant data; further studies need to be done at the mechanistic level.

## Conclusions

This study highlights the relevance of a miRNA-sEV mediated mechanism by which pericardial Mφs decrease their basal immuno-protective capacity in CAD patients. This study opens frontiers for further studies aiming at developing therapeutics against the immunological remodeling of PF Mφ in heart diseases.

Perspectives **COMPETENCY IN MEDICAL KNOWLEDGE:** This study provides the first evidence of a role for pericardial sEVs and immune cells in CAD. Clinical trials aimed at generating sEV-like molecules have had limited effects on resolving atherosclerosis so far.

**TRANSLATIONAL OUTLOOK:** This study has identified a role of pericardial fluid sEV as local anti-inflammatory regulators of immune cells. In CAD, the pericardial sEVs are dysfunctional and their microRNA cargo is altered, suggesting a therapeutic potential of intrapericardial delivery of lipidic nanoparticles and exogenous sEVs engineered to correct the microRNA defect to restore anti-inflammatory macrophage function. Moreover, this study advocates for the preservation and repair of the pericardial sac in cardiovascular surgery procedures whenever possible, and opens avenues for pericardial fluid therapeutics as well as bioengineering approaches to restore the cardioprotective action of the pericardial niche.

## Funding Support and Author Disclosures

This study was supported by the British Heart Foundation (BHF) Programme Grant-RG/15/5/31446 and Personal Chair Awards-CH/15/1/31199 (to Dr Emanueli); BHF Transitional Fellowship (Imperial College London BHF Centre of Research Excellence-RE/18/4/34215) and BHF Project Grant (BHF- PG/23/11336) (both to Dr Ben-Aicha); UKRI Postdoctoral Fellowship-EP/X023729/1 (to Dr Martino); a grant from The Leducq Foundation (to Dr Sattler); PLEC2021–007664-Unión Europea NextGeneration EU/PRTR, grant PID2021-128891OB-I00, and grant M-ERA-NET-3 / PCI2023-143431 - European Funds (all to Dr Vilahur). This study was also funded by MCIN/AEI/10.13039/501100011033 and Fondo Europeo de Desarrollo Regional, A way of making Europe. This manuscript was also supported by the UK National Institute of Heart Research (NIHR) for funding this research. The authors have reported that they have no relationships relevant to the contents of this paper to disclose.

## Appendix

Video 1 ▪▪▪
