# Supplementary material for: Small Extracellular Vesicles in the Pericardium Modulate Macrophage Immunophenotype in Coronary Artery Disease
Source: JACC Basic Transl Sci. 2024 Jul 3;9(9):1057–72. doi: 10.1016/j.jacbts.2024.05.003 (PMC11494395; doi:10.1016/j.jacbts.2024.05.003)
Supplement: Supplemental Material [file mmc2.pdf]

Supplemental Figure 1

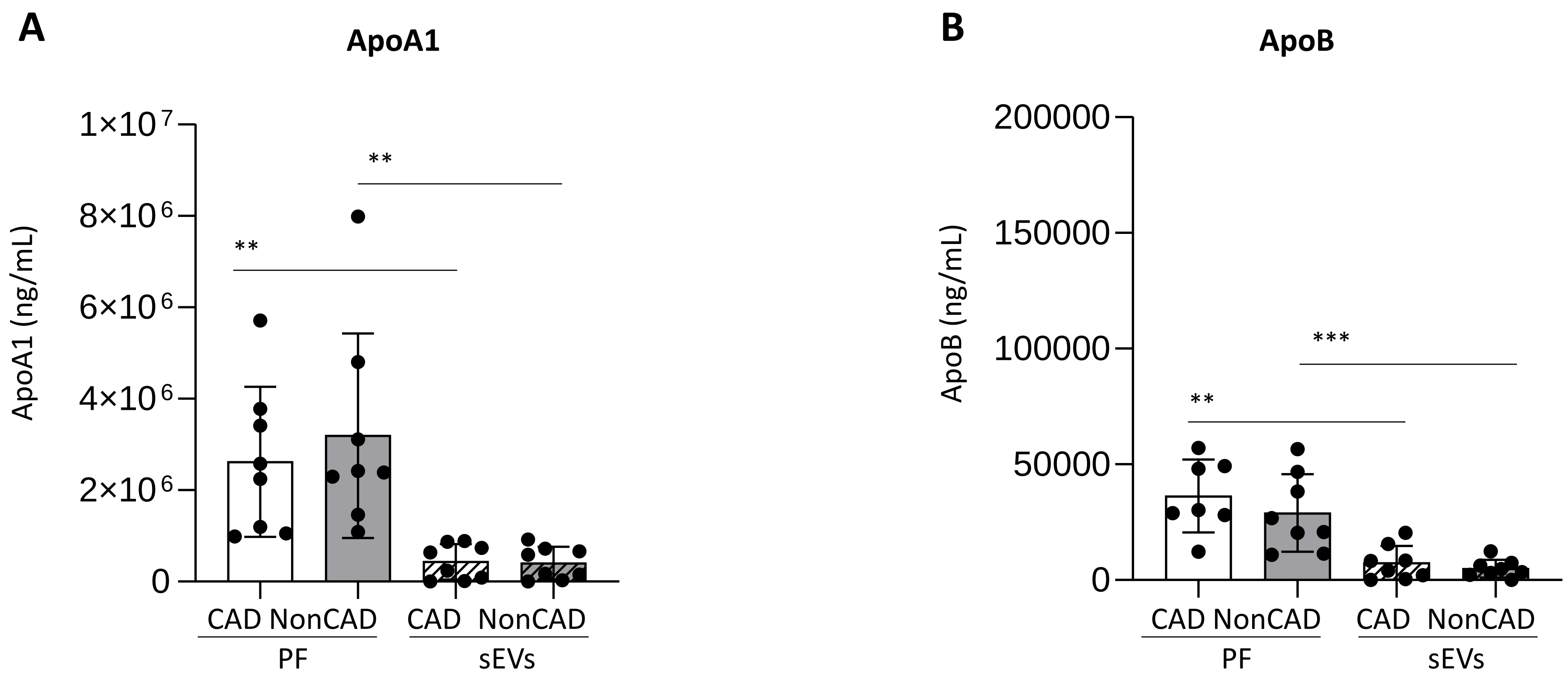

Supplemental Figure 1. Assessment of Small Extracellular Vesicle contamination by lipoproteins. To validate the level of purity of the pericardial sEV preparations, we compared the ApoA1 and ApoB presence in the sEV vs the lipoprotein level the whole pericardial fluid donated by CAD and NonCAD patients (n=8 per group). The detected levels were lower in sEV compared to full pericardial fluid. NO significant differences were observed between CAD and NonCAD groups in either pericardial fluid or small extracellular vesicle samples. Data reported as mean±SD. T-Test (A,B) was performed (significance: p-value< 0.05). *p<0.05, **p<0.01 and ***p<0.001

Supplemental Figure 2

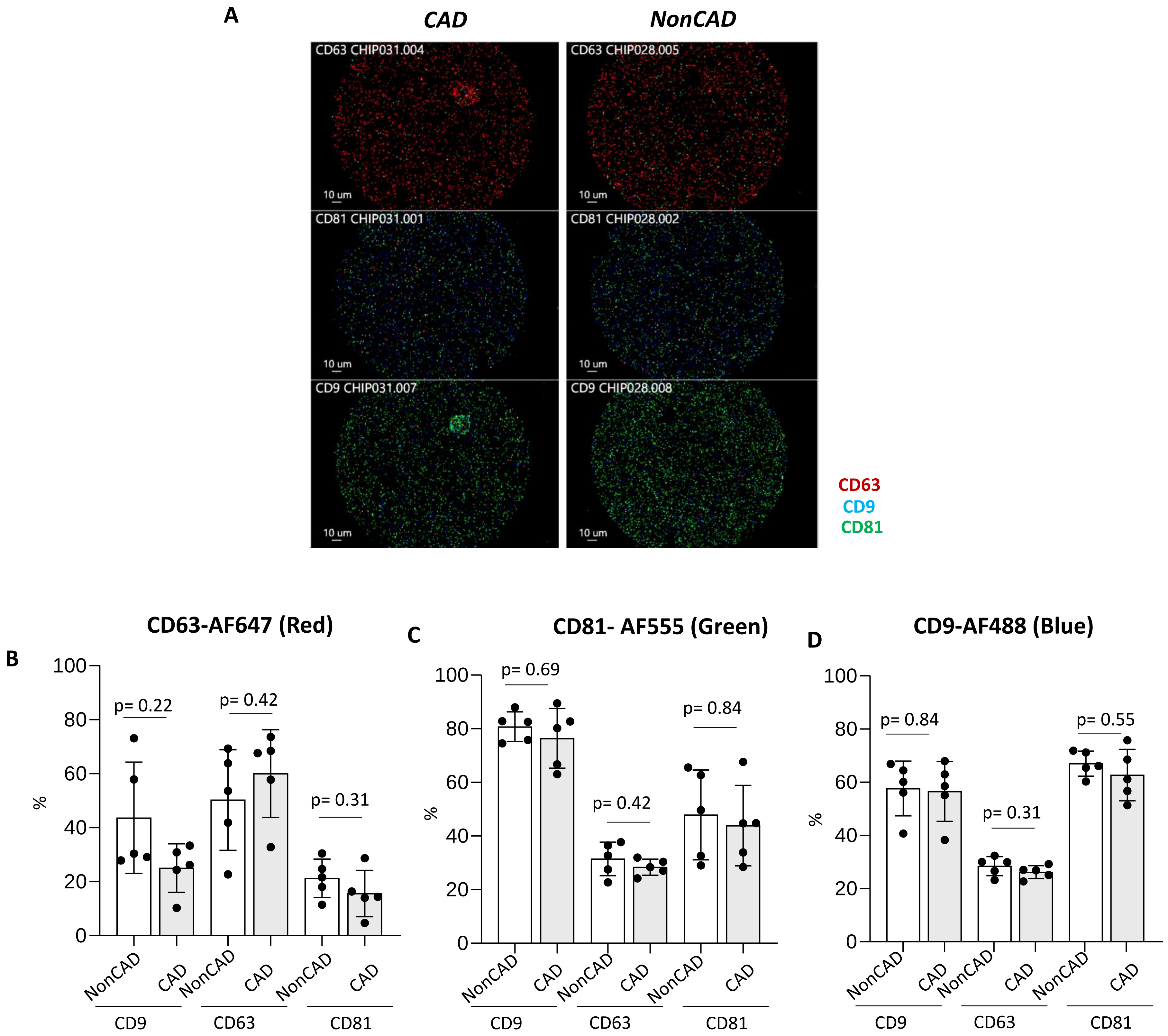

Supplemental Figure 2. Detailed characterization of the CAD and non-CAD pericardial fluid small extracellular vesicles (sEV) using ExoView R100 Platform. Tetraspanins levels (CD9, CD63, or CD81) were detected in sEV isolated from PF CAD and NonCAD patients (n=5 per group). With ExoView-R100, sEV analysis can be performed to determine the tetraspanins (CD9, CD63, CD81) profile of sEVs. An ExoView chip (CHIP) specifically captures sEVs via a microarray tetraspanin chip functionalized with antibodies. sEVs that carry specific proteins bind to the corresponding antibodies on the chip. ExoView analyzes sEVs using visible light interference for size measurements and fluorescence for protein profiling. A) ExoView fluorescence imaging data. Each fluorescent spot corresponds to a sEV. CD9 (blue), CD63 (red), CD81 (green). Size bar: 10 micrometer; B) CD63 coated CHIP sEV profile; C) CD81 coated CHIP sEV profile; D) CD9 coated CHIP sEV profile. Mann-Whitney test was performed ($p<0.005$) “CHIP” refers to arbitrary codes given to each chip from the supplier.

Supplemental Figure 3

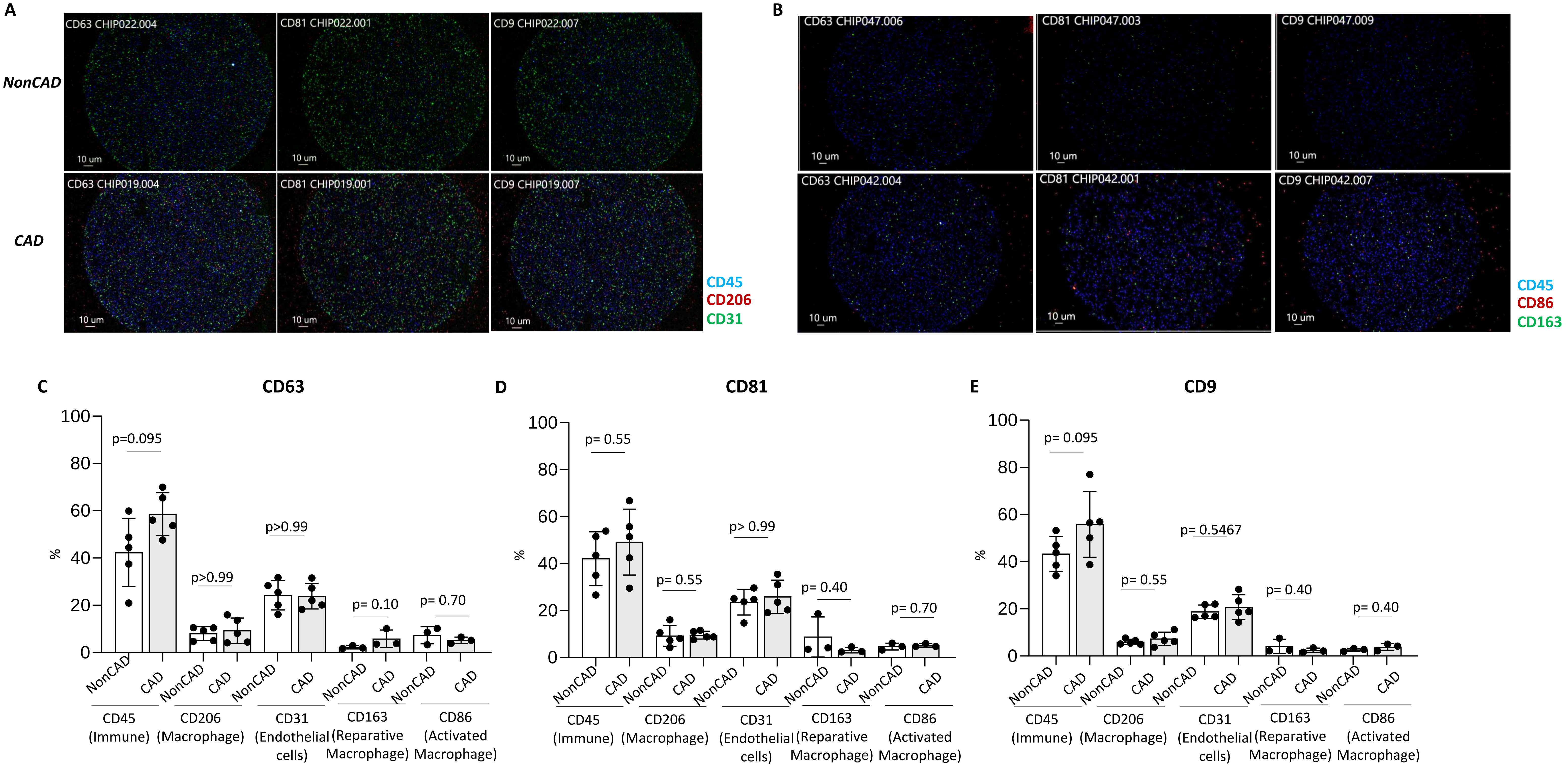

Supplemental Figure 3. Pericardial fluid small extracellular vesicles (cell markers) characterization in CAD and NonCAD patients. Cell markers (CD45, CD206, CD31, CD163, CD86) levels detected in sEV isolated from PF from patients undergoing CABG surgery (CAD; n=3-5) and MVR (NonCAD; n=5). A) CD45 is presented in blue fluorescence, CD206 in red and CD31 in green. B) CD45 is presented in blue fluorescence, CD86 in red and CD163 in green. Size bar: 10 micrometer; C) CD63 quantification; D) CD81 quantification; E) CD9 quantification) Mann-Whitney test was applied ($p<0.005$). “CHIP” refers to arbitrary codes given to each chip from the supplier.

Supplemental Figure 4

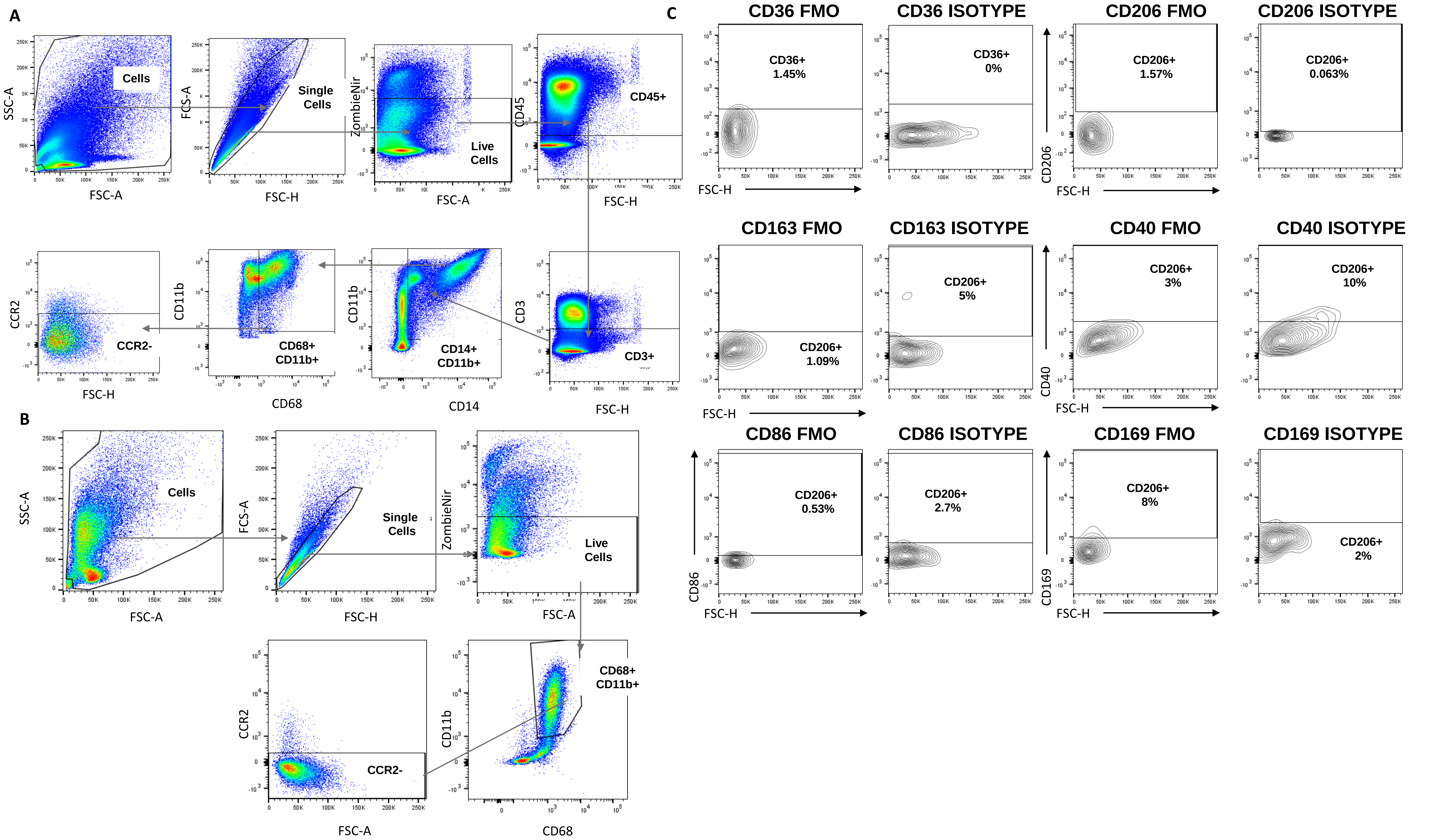

Supplemental Figure 4. Flow Cytometry analysis details A) Gating strategy for human pericardial fluid macrophages. Initial selection involved live cells expressing CD45 and lacking CD3. Subsequent gating focused on monocyte/macrophage identification (CD14+ CD11b), followed by a deeper selection for macrophages (CD68+ CD11b+ CCR2-). . B) Sequential gating of in vitro macrophages progressing from cells to singlets, followed by isolation of viable cells. Subsequent gating targeted the identification of macrophages (CD11b+ CD68+), with further selection based on CCR2-. Controls included Fluorescence Minus One (FMO) and Isotype controls (C).

Supplemental Figure 5

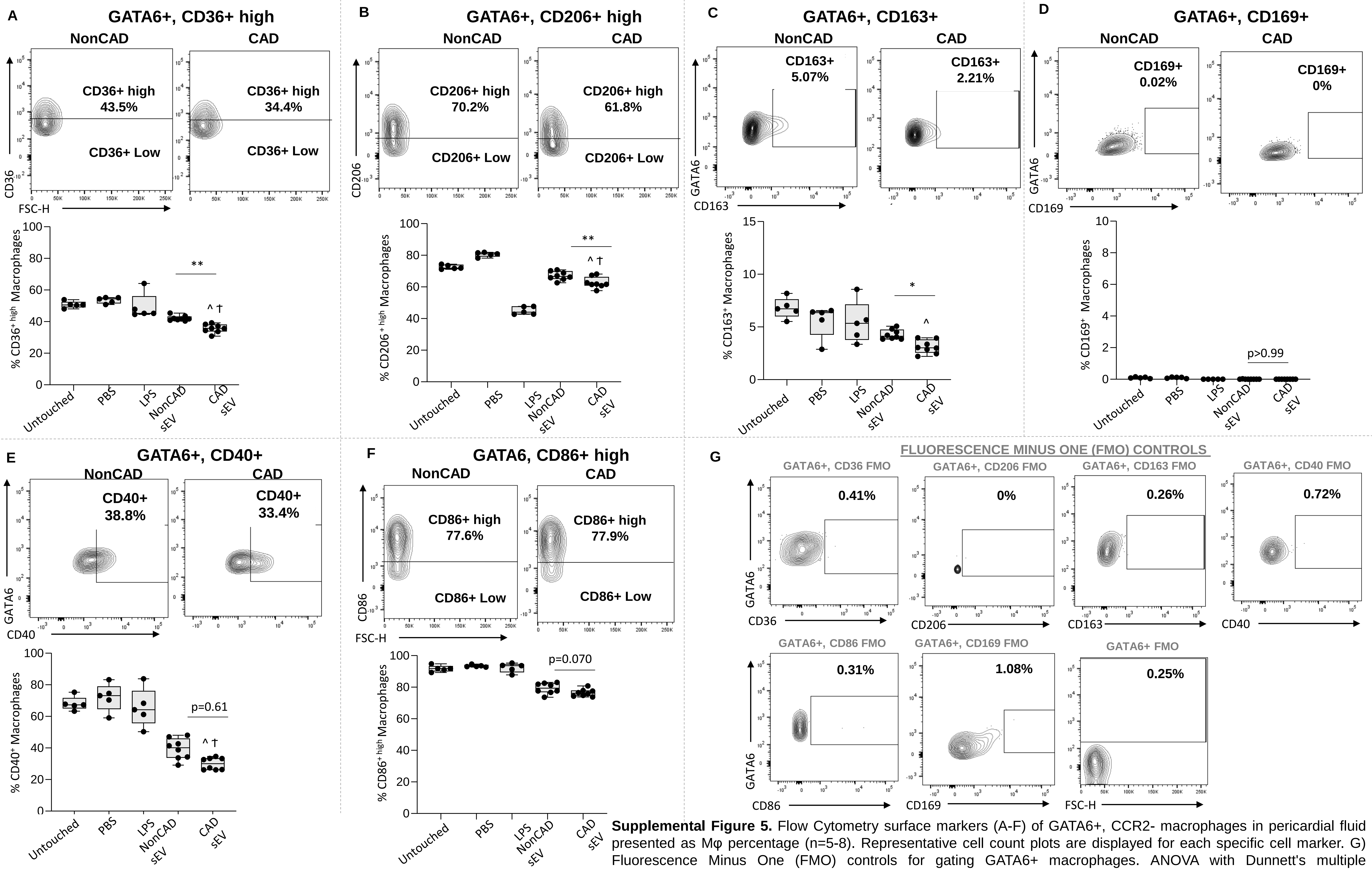

Supplemental Figure 5. Flow Cytometry surface markers (A-F) of GATA6+, CCR2- macrophages in pericardial fluid presented as Mφ percentage (n=5-8). Representative cell count plots are displayed for each specific cell marker. G) Fluorescence Minus One (FMO) controls for gating GATA6+ macrophages. ANOVA with Dunnett's multiple comparisons (*A-C,F*) or Kruskal-Wallis with Dunn's multiple comparisons (*D-E*) test with multiple comparisons were performed for normal or non-normal distributed data respectively. ^ p<0.05 vs PBS † p<0.05 vs LPS Data reported as median [IQR].CAD: Coronary artery disease; NonCAD: NonCoronary artery disease. *p<0.05, **p<0.01 and ***p<0.001

Supplemental Figure 6

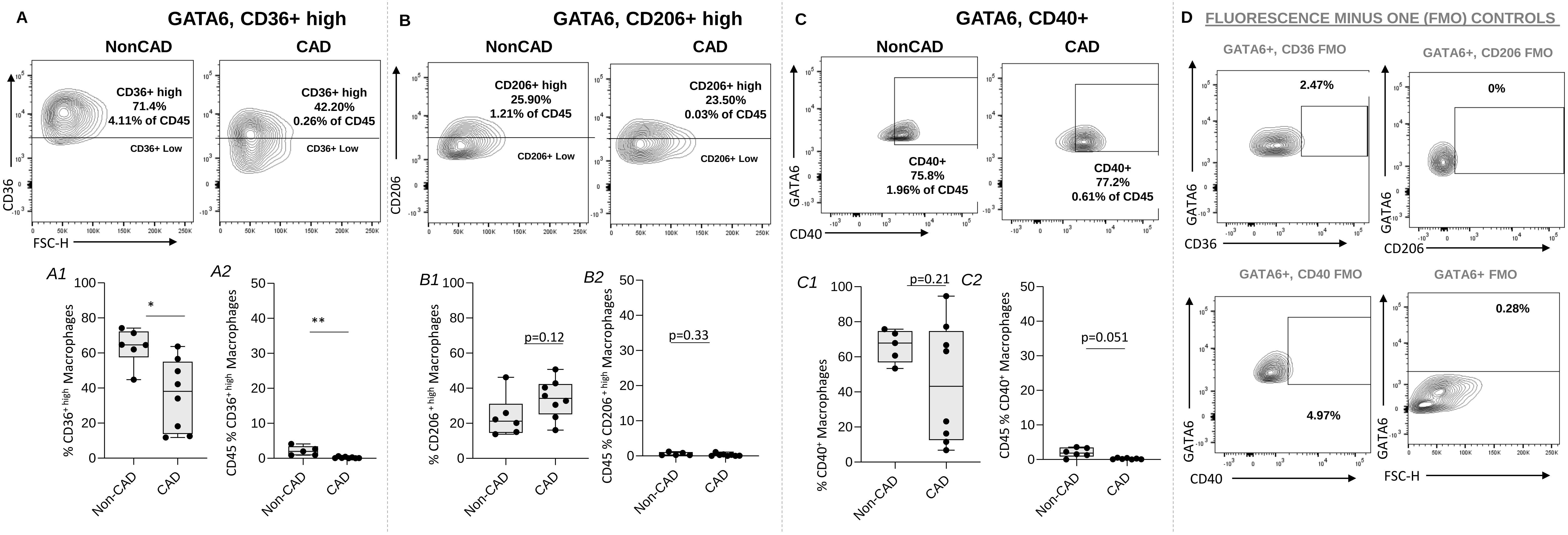

Supplemental Figure 6. Flow Cytometry surface markers CD36$^{+/high}$ (A), CD206$^{+/high}$ (B), and CD40^{+} (C) expression on GATA6^{+}, CCR2^{-} macrophages in pericardial fluid presented as Mφ percentage. Cells as percentage of CD45 is also depicted (n=7-8). Representative cell count plots are provided for each specific cell marker. D) Fluorescence Minus One (FMO) controls for gating GATA6^{+} macrophages. T-Test (*A1*) or Mann-Whitney (*B1-C1; A2-C2*) test were performed for normal or non-normal distributed data respectively. Data reported as median [IQR]. CAD: Coronary artery disease; NonCAD: NonCoronary artery disease. *p<0.05, **p<0.01 and ***p<0.001

Supplemental Figure 7

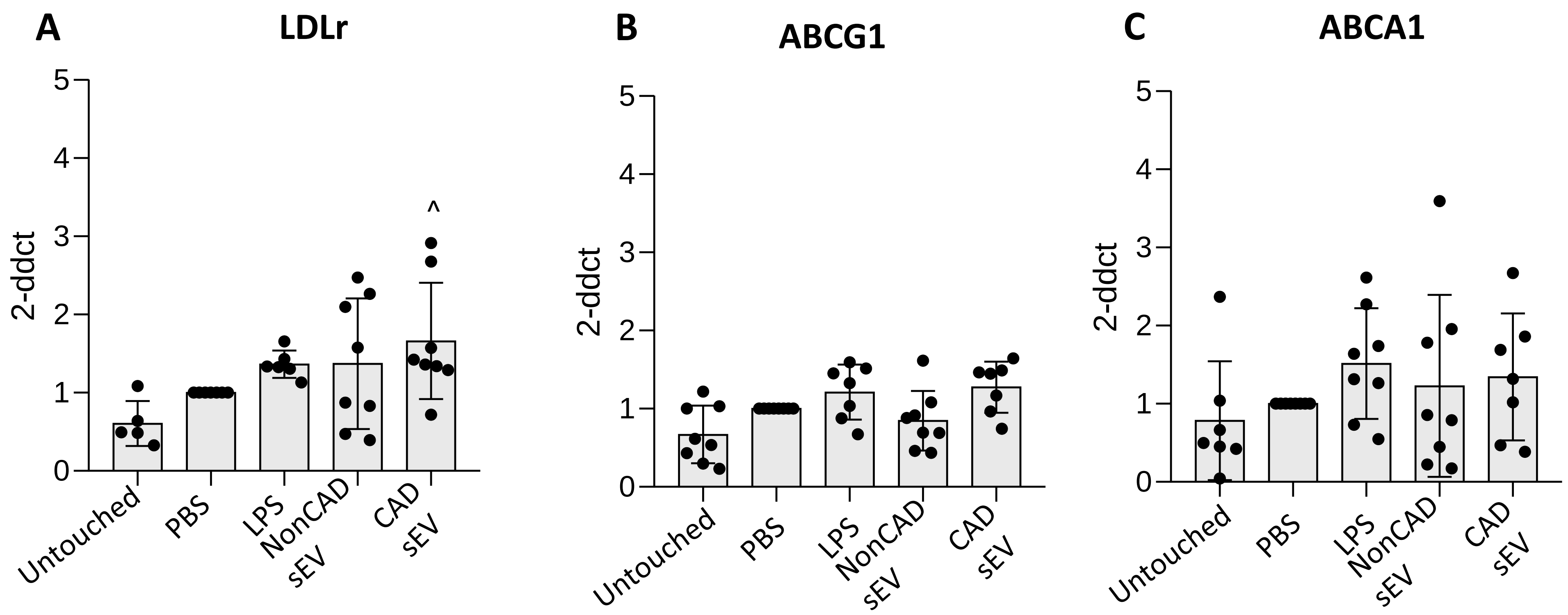

Supplemental Figure 7. Lipoprotein receptor mRNA expression in macrophages exposed to pericardial sEVs from CAD and Non-CAD patients. Lipoprotein receptors LDLr, ABCG1 and ABCA1 mRNA levels were measured qPCR and normalized to PBS. A) LDLr B) ABCG1, C) ABCA1. Data reported as mean ± SD. Kruskal-Wallis test with Dunn's multiple comparisons were performed(*A-C*). ^ $p<0.05$ vs PBS; ☨ $p<0.05$ vs LPS. CAD: Coronary artery disease; NonCAD; NonCoronary artery disease.

Supplemental Figure 8

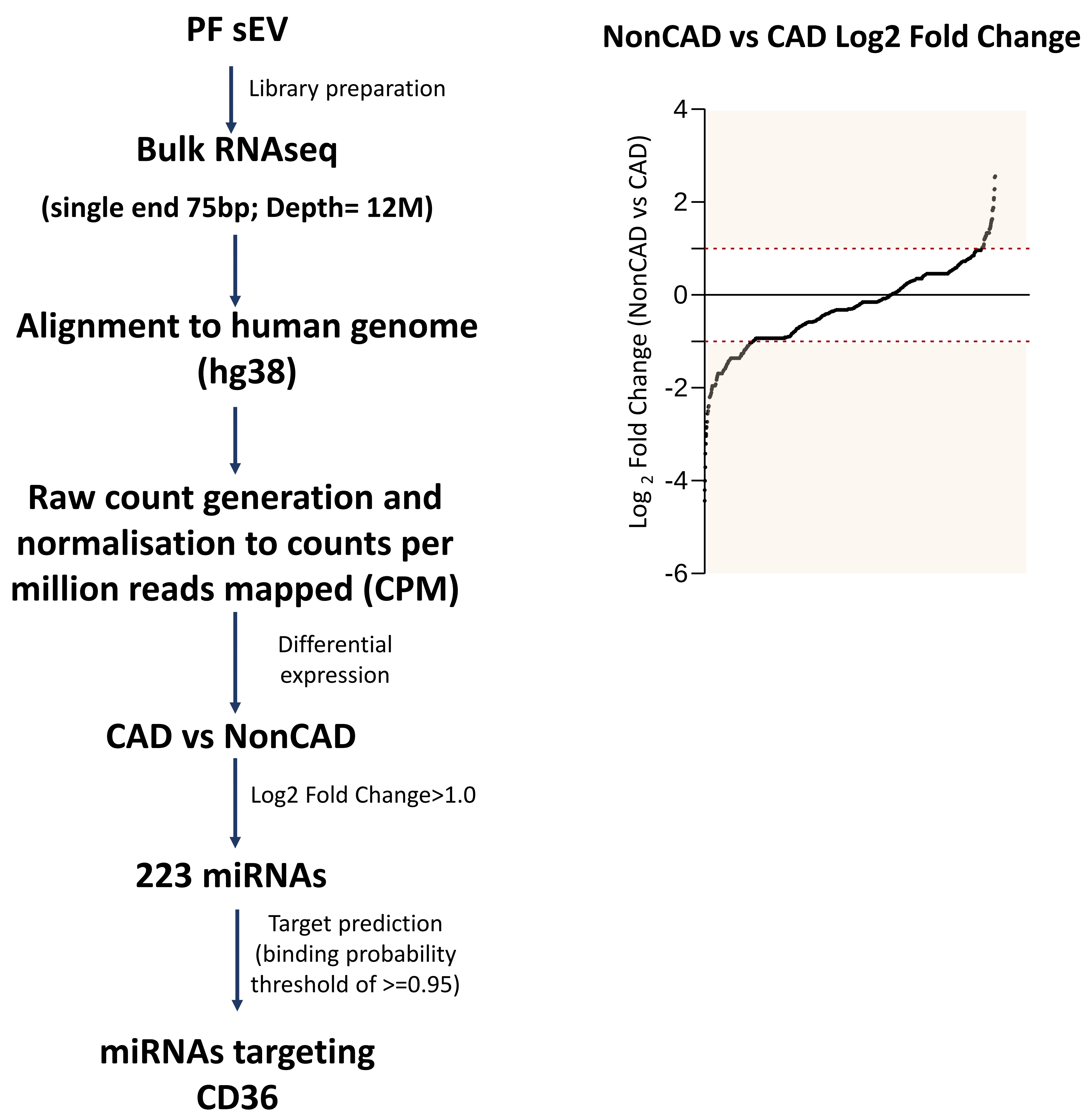

Supplemental Figure 8. Illustrates the flow followed for the small RNA-seq protocol. The samples underwent small RNA sequencing, and data was aligned and compared (CAD vs NonCAD). Further bioinformatics expanded in methods were employed to discern potential miRNAs that may target CD36. Representative graphic of the Log2Fold Change of the all detected miRNAs (specified in Supplemental Table 7). 12M= 12 Million reads.

Supplemental Figure 9

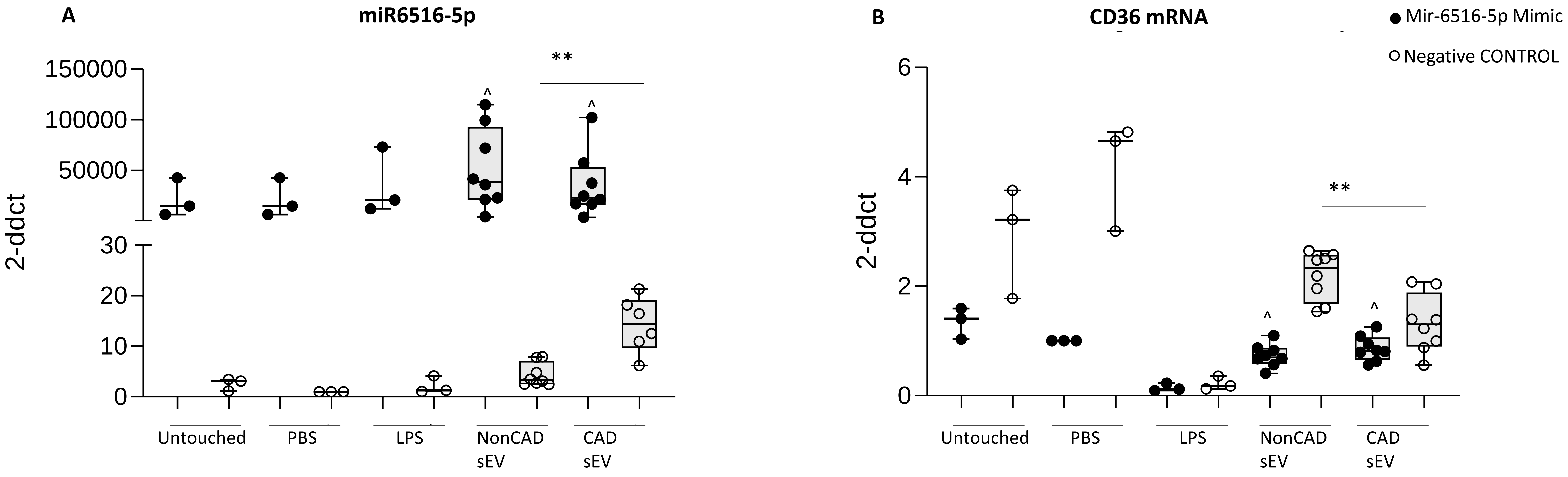

Supplemental Figure 9. Differential expression of CD36 mRNA induced by miR-6516-5p overexpression in buffy-coat derived macrophages. Macrophages were transfected with a miR-6516 mimic or negative control (10pmol) and then either left untouched or exposed to one of the following treatments: PBS, LPS (1mg/mL), pericardial fluid sEV from either NonCAD or CAD (5x107 particles/mL). The expression of miR-6516-5p and its predicted target gene CD36 were determined by qPCR after 24h from sEV treatment. Data were analysed using the 2ddct formula . Data are reported as median [IQR]. n=3-8 A) Normalized miR-6516-5p expression; B) Normalized CD36-mRNA expression. T-Test (*B*) or Mann Whitney (*A*) was performed. ^p<0.05 for comparison vs the negative control of miRNA mimic within any macrophage treatment group. *p<0.05, **p<0.01 and ***p<0.001

Supplemental Figure 10

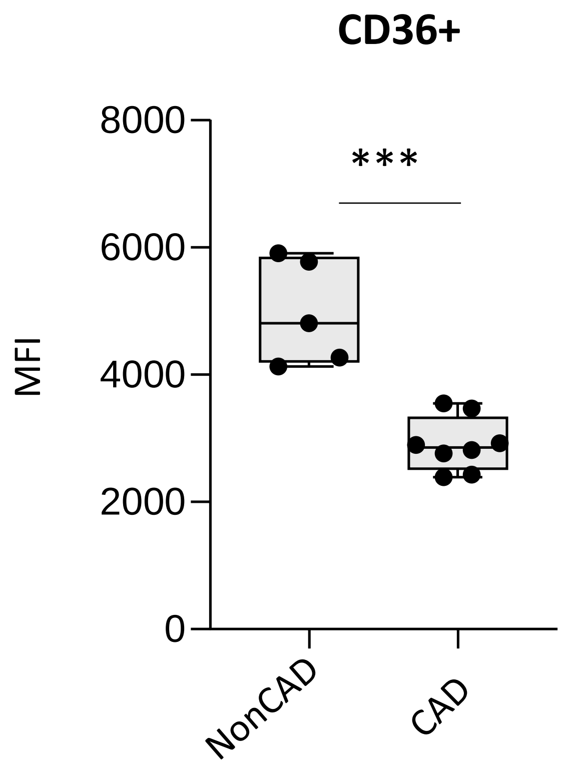

Supplemental Figure 10. CD36 levels in pericardial fluid macophages. CD36 surface levels are decreased in CAD PF macrophages compared to Non-CAD. n=5-8. T-test was performed. Data reported as median [IQR]. CAD: Coronary artery disease; NonCAD; NonCoronary artery disease; Mφ: Macrophage. *p<0.05, **p<0.01 and ***p<0.001

Supplemental Figure 11

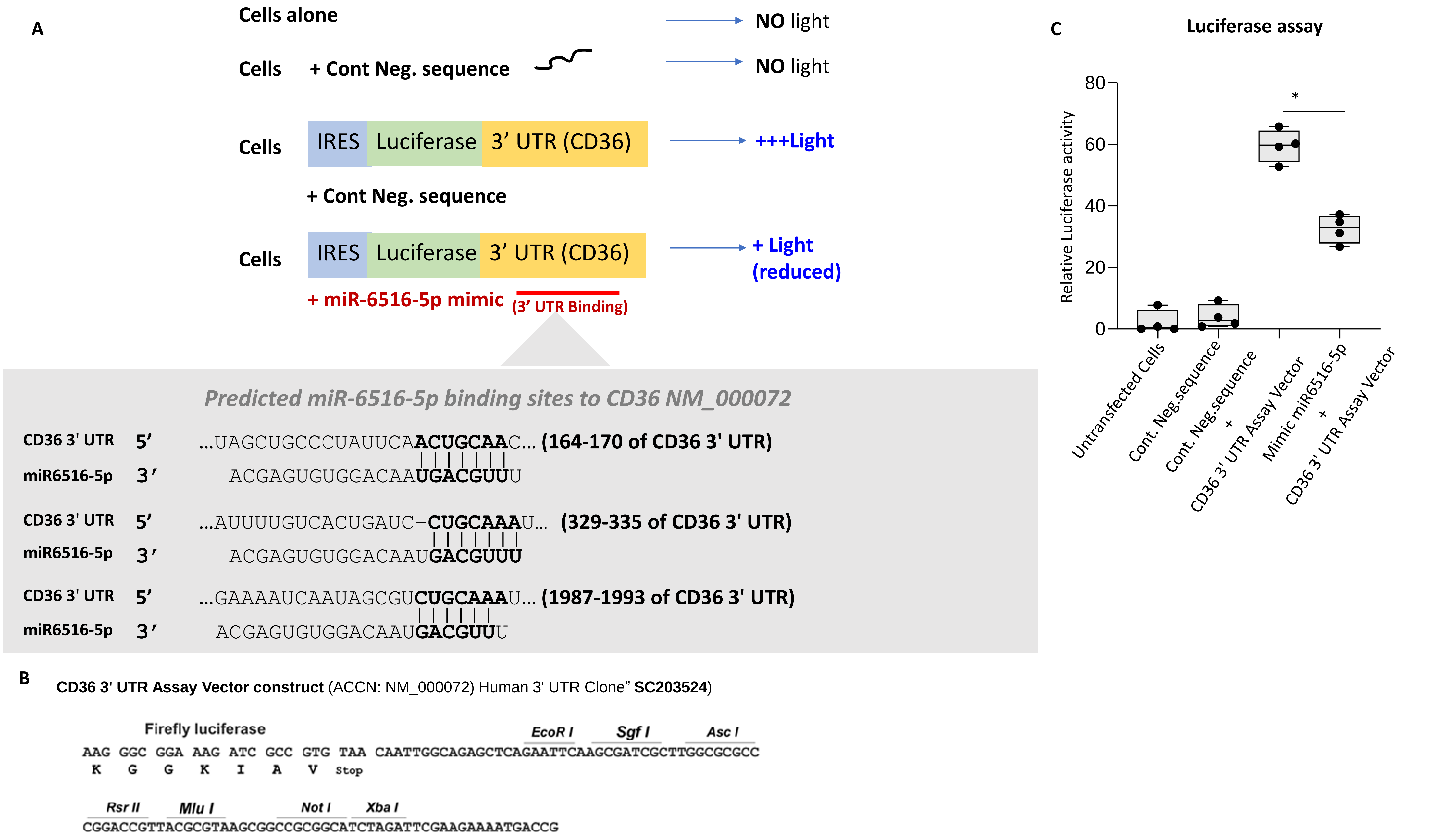

Supplemental Figure 11. Validation of miR-6516-5p direct binding to the predicted CD36 target gene using 3’UTR-luciferase assay A) Predicted miR-6516-5p binding sites to the 3’UTR of CD36 mRNA miR6516-5p mimic sequence (Chr. 17 - 77089417 – 77089497) by TargetScan. B) CD36 3' UTR Assay Vector construct (ACCN: NM_000072) Human 3' UTR Clone” SC203524 (Cont Neg Ref:4464058) C) ‘3-UTR luciferase assay results. Data presented as median [IQR] and analyzed by Mann-Whitney test. "Cont. Neg." refers to the control negative sequence. ACCN denotes the Accession number assigned by NCBI. *p<0.05, **p<0.01 and ***p<0.001

Supplemental Figure 12

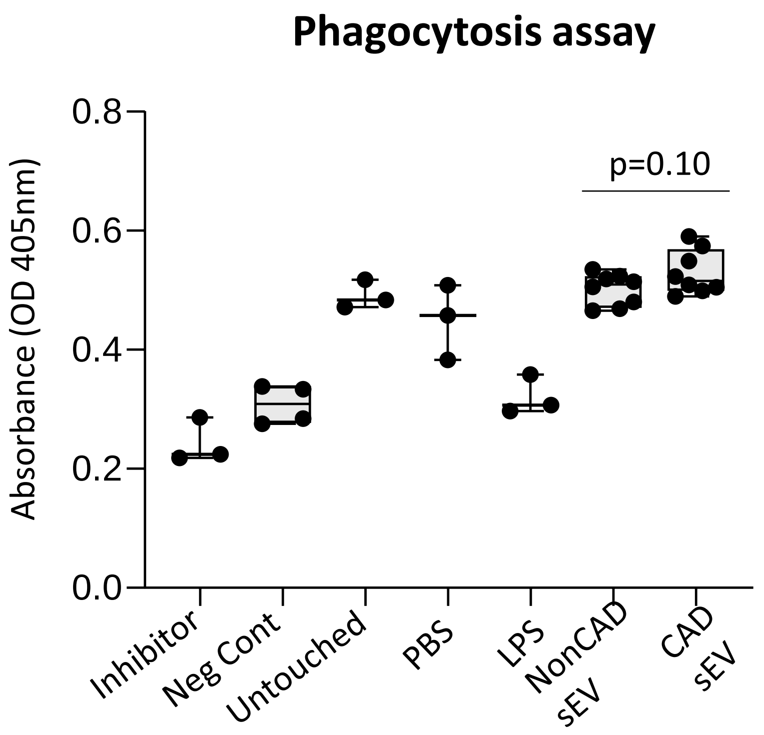

Supplemental Figure 12. Macrophages phagocytic capacity. Phagocytosis assay from *in vitro* macrophages following a commercial kit protocol (ab211156) which includes a phagocytosis inhibitor control. Negative control represents cells without phagocytosis kit substrate. Data presented as median [IQR] and analyzed by T-test. Coronary artery disease; NonCAD; NonCoronary artery disease *p<0.05, **p<0.01 and ***p<0.001

Supplemental Table 1: Patients characteristics		
	**CAD (CABG) (n=8)**	**NonCAD (MVR) (n=8)**
**Age (years)**	61 (± 10)	62(± 10)
**Sex ***	8 M	5M 3F
**Weight (Kg)**	84.1(± 9.0)	77.5(± 13.6)
**BMI**	28.4(± 3.4)	27.2 (± 4.7)
**Diabetes (%)**	0	0
**Heart Failure (NHYA %)**	I (50) II (50) III (0)VI (0)	I (25) II (25) III (37.5)VI (12.5)
**LV function (%)****	1 (75) 2(25) 3(0)	1 (100) 2(0) 3(0)
**Number of vessels affected (%)*****	0(0) 1(0) 2(12.5) 3(87.5)	0(100) 1(0) 2(0) 3(0)
**> 50% disease in the left main stem (%)**	37,5	0
**Previous MI (%)**	50	0
**Smoker (%)**	0 / Ex(50)	12.5 / Ex(25)
**CVD family history (%)**	100	37,5
**Hypertension (%)**	87,5	62,5
**Hypercholesterolemia (%)**	87,5	50
**Hypertyroidism (%)**	12,5	12,5
**Atrial fibrillation (%)**	12,5	12,5
**Preoperative medications (%)**		
Aspirin	75	12,5
Clopidogrel	62,5	0
Beta blockers	87,5	25
Calcium Antagonist	37,5	12,5
Oral Nitrates	50	0
Statin	87,5	50
ACE inhibitors	75	62,5
Angiotensin blockers	12,5	0
Diuretics	12,5	12,5
**Medications at discharge (%)**		
Aspirin	87,5	12,5
Clopidogrel	50	12,5
Beta blockers	100	75
Calcium Antagonist	12,5	12,5
Oral Nitrates	12,5	0
Statin	87,5	50
ACE inhibitors	50	75
Angiotensin blockers	0	0
Diuretics	75	62,5

Legend

*		
M		Male
F		Female
**		
	1	Good (>50%)
	2	Moderate (30-50%)
	3	Poor (<30%)
***		
	0	None
	1	Single
	2	Double
	3	Triple

Supplementel Table 1: Patient Demographics for Pericardial Fluid Collection in the ARCADIA Study (REC13/LO/1687 and 17/WA/0161) for sEV Sample Acquisition.

Supplemental Table 2: Antibody information				
**ANTIBODY NAME**	**COMPANY**	**REFERENCE**	**Fluorophore**	**Dilution**
**CD11b**	Biolegend	101251	Brilliant Violet 421	1:200
**CD68**	Biolegend	333811	Alexa Fluor® 488	1:200
**CD206**	Biolegend	321110	APC	1:100
**CD40**	Biolegend	334308	PE	1:100
**CD36**	Biolegend	336221	PE/Cyanine7	1:200
**CD163**	Biolegend	326510	APC	1:100
**CD169**	Biolegend	346008	PerCP/Cyanine5.5	1:100
**CD86**	Biolegend	305406	PE	1:100
**SRB1**	Biolegend	363206	PerCP/Cyanine5.5	1:100
**GATA6**	Cell Signaling Technology	26452S	PE	1:100
**CD45**	Biolegend	304022	Pacific Blue™	1:200
**CD3**	Biolegend	317330	Brilliant Violet 785	1:200

Supplemental Table 3: Reagdent information		
**DETECTION ASSAY**	**COMPANY**	**CODE**
**IL1A qPCR primer**	Life technologies	Hs00174092_m1
**IL1B qPCR primer**	Life technologies	Hs01555410_m1
**TNFA qPCR primer**	Life technologies	Hs00174128_m1
**MRC1 qPCR primer**	Life technologies	Hs00267207_m1
**ABCG1 qPCR primer**	Life technologies	Hs00245154_m1
**ABCA1 qPCR primer**	Life technologies	Hs01059101_m1
**CD36 qPCR primer**	Life technologies	Hs00354519_m1
**SCARB qPCR primer**	Life technologies	Hs00969821_m1
**LDLr qPCR primer**	Life technologies	Hs01092524_m1
**GAPDH qPCR primer**	Life technologies	Hs02786624_g1
**miR-6516-5p qPCR primer**	Qiagen	YP02117973
**Inhibitor miR-6516-5p**	Life technologies	ID: MH29784
**Mimic miR6516-5p**	Life technologies	ID: MC29784

Suplemental Table 4: Median fluorescence intensity (MFI) values

A. Pericardial Fluid cells Median fluorescence intensity (MFI) (Mean±SD)

	NONCAD (n=8)	CAD (n=6)	p-value
**CD36+ high**	8610 (± 1502)	8183 (±3392)	0.9497
**CD206+high**	2020 (±297.7)	2443 (±308.6)	0.0813
**CD163+**	1725 (±167.2)	2821 (±1271)	0.0859
**CD169+**	5104 (±1198)	5569 (±3808)	0.7987
**CD40+**	2496 (±662.2)	2805 (±266.2)	0.4908
**CD86+high**	2266 (±162.9)	2064 (±129.5)	0.0834

B. *In Vitro* Median fluorescence intensity (MFI) (Mean±SD)

	Untouched	PBS	LPS	NONCAD (n=8)	CAD (n=8)	p-value
**CD36+ high**	863.6 (±47.18)	914.2 (±55.87)	792.2 (±54.56)	769.8 (±26.47)	712.1 (±46.87)	**0.0174**
**CD206+high**	1669 (±63.84)	2020 (±103.5)	1181 (±24.29)	1519 (±49.52)	1480 (±64.94)	>0.9999
**CD163+**	811 (±33.26)	799.6 (±34.86)	637.3 (±31.72)	701.1 (±25.27)	709.6 (±54.39)	>0.9999
**CD169+**	1647 (±18.24)	1486 (±172.8)	1456 (±15.52)	1411 (±17.37)	1354 (±18.28)	0.1409
**CD40+**	2382 (±131.9)	2476 (±260.6)	2399 (±585)	1706 (±133.5)	1528 (±75.16)	0.711
**CD86+high**	6122 (±253.9)	5604 (±390)	9672 (±1924)	4956 (±192.8)	5021 (±204.9)	0.9994

C. Pericardial Fluid GATA6+ macrophages Median fluorescence intensity (MFI) (Mean±SD)

	NONCAD (n=8)	CAD (n=6)	p-value
**CD36+ high**	10265 (± 1505)	6705 (±2756)	**0,020**
**CD206+high**	4107 (±1038)	3527 (±1403)	0,85
**CD40+**	3127 (±557.4)	3859 (±767.1)	0,06

D. *In Vitro* GATA6+ macrophage Median fluorescence intensity (MFI) (Mean±SD)

	Untouched	PBS	LPS	NONCAD (n=8)	CAD (n=8)	p-value
**CD36+ high**	1075 (±55.37)	1162 (±71.83)	1084 (±196.6)	968.1 (±42.85)	926.5 (±31.57)	0,55
**CD206+high**	6909 (±251.5)	6248 (±335.2)	11375 (±2912)	5263 (±253.9)	5305 (±184.4)	>0.99
**CD163+**	1861 (±58.4)	1899 (±128.3)	1597 (±91.99)	1697 (±79.55)	1646 (±136.5)	0,76
**CD40+**	3705 (±177.9)	3600 (±236.1)	3745 (±369.7)	3119 (±152.4)	3165 (±87.93)	0,55
**CD86+high**	6909 (±251.5)	6248 (±335.2)	11375 (±2912)	5263 (±253.9)	5305 (±184.4)	>0,99

Supplemental Table 4 provides the Median Fluorescence Intensity Values of macrophage subtype markers derived from both pericardial fluid (PF) (A and C) macrophages and *in vitro* macrophages obtained from buffy coats (B and D). Tables A and B show the MFI values for the macrophages immunophenotyping. Tables C and D show the MFI values for the GATA6+ macrophage suppopulation analysis. A and C) P-values are presented for T-tests or Mann-Whitney tests, comparing CAD vs. Non-CAD groups for normal or non-normal distributed data respectively.
B and D) ANOVA or Kruskal-Wallis test with multiple comparisons were performed for normal or non-normal distributed data respectively. Data

**Supplemental Table 5: sEV Colocalisation counts in total sEV percentege**																					
**Average Colocalization Counts (%)**																					
**CAD**	**CD63**					**CD81**					**CD9**					**IgG**					
	**CHIP031 (%)**	**CHIP047 (%)**	**CHIP068 (%)**	**MEAN**	**SD**	**CHIP031 (%)**	**CHIP047 (%)**	**CHIP068 (%)**	**MEAN**	**SD**	**CHIP031 (%)**	**CHIP047 (%)**	**CHIP068 (%)**	**MEAN**	**SD**	**CHIP031 (%)**	**CHIP047 (%)**	**CHIP068 (%)**	**MEAN**	**SD**	**Total Colocalization mean**
CD63	59,07	60,66	45,97	55,23	6,58	4,84	5,00	9,38	6,41	2,10	5,07	6,88	11,31	7,76	2,62	12,71	10,68	35,84	19,74	11,41	
CD81	5,15	3,56	6,97	5,23	1,39	7,77	2,96	6,40	5,71	2,02	28,10	28,71	31,65	29,49	1,55	3,01	2,08	7,08	4,06	2,17	
CD9	20,94	20,42	27,02	22,79	2,99	40,32	52,33	37,58	43,41	6,40	26,51	19,29	15,15	20,32	4,69	79,60	87,24	49,12	71,98	16,47	
CD63/CD81	2,50	1,85	4,28	2,88	1,03	3,62	2,00	5,30	3,64	1,35	6,19	8,60	8,48	7,76	1,11	0,00	0,00	0,44	0,15	0,21	
CD63/CD9	3,26	2,40	2,20	2,62	0,46	8,23	13,96	10,49	10,89	2,35	3,75	4,50	2,36	3,54	0,89	0,00	0,00	3,10	1,03	1,46	
CD81/CD9	4,21	7,88	8,92	7,00	2,02	22,56	14,15	20,47	19,06	3,58	18,59	22,44	21,41	20,82	1,63	0,67	0,00	2,21	0,96	0,93	
CD63/CD81/CD9	1,65	3,15	4,52	3,11	1,17	9,49	9,61	10,32	9,81	0,37	6,53	9,54	9,56	8,55	1,42	0,33	0,00	1,77	0,70	0,77	
Total				3,9					10,85					10,16					0,71		6,41
**NonCAD**	**CHIP028 (%)**	**CHIP048 (%)**	**CHIP050 (%)**			**CHIP028 (%)**	**CHIP048 (%)**	**CHIP050 (%)**			**CHIP028 (%)**	**CHIP048 (%)**	**CHIP050 (%)**			**CHIP028 (%)**	**CHIP048 (%)**	**CHIP050 (%)**			
CD63	58,48	33,50	19,65	37,21	16,07	4,01	8,26	5,08	5,78	1,80	3,81	8,09	10,45	7,45	2,75	10,36	10,09	8,72	9,72	0,72	
CD81	5,50	5,22	4,19	4,97	0,56	8,15	4,75	4,42	5,77	1,69	27,66	25,43	37,32	30,14	5,16	2,14	1,83	0,82	1,60	0,57	
CD9	17,49	42,86	67,66	42,67	20,48	36,28	43,43	57,42	45,71	8,78	24,63	28,62	19,68	24,31	3,66	73,93	88,07	89,92	83,97	7,14	
CD63/CD81	2,94	2,36	1,66	2,32	0,52	3,92	4,10	2,24	3,42	0,84	6,85	10,07	9,13	8,68	1,35	0,00	0,00	0,00	0,00	0,00	
CD63/CD9	3,32	2,27	1,10	2,23	0,90	8,15	15,63	8,31	10,69	3,49	3,73	2,81	1,52	2,69	0,91	0,36	0,00	0,54	0,30	0,23	
CD81/CD9	6,54	10,44	4,97	7,32	2,30	25,64	14,14	15,36	18,38	5,16	21,32	16,07	15,72	17,70	2,56	1,07	0,00	0,00	0,36	0,51	
CD63/CD81/CD9	2,32	3,35	0,88	2,19	1,01	10,39	9,69	7,19	9,09	1,38	7,79	8,86	6,19	7,61	1,10	0,36	0,00	0,00	0,12	0,17	
Total				3,51					10,40					9,17					0,19		5,82

Supplemental Table 5: sEV Colocalisation counts in %: Percentages of the colocalization in sEV tetraspanins.“CHIP” refers to arbitrary codes given to each chip from the supplier.

Supplemental Table 6				
Predicted sEVs-miRNA targeting CD36				
**Name**	**Identifier**	**Log_2 fold change(NonCAD-CAD)**	**Fold change**	**Target of interest**
hsa-miR-3934-3p	URS000075B5A4_9606	-1,55953012	-2,947578263	CD36
hsa-miR-6516-5p	URS000075A0B9_9606	-1,098410002	-2,141185817	CD36
hsa-miR-7151-3p	URS000075CA04_9606	-0,933161949	-1,909456361	CD36
hsa-miR-532-3p	URS00004B4B85_9606	-0,685515732	-1,608276803	CD36
hsa-miR-619-5p	URS000075B584_9606	-0,12274674	-1,088805862	CD36

Supplemental Table 7. All sequencing detected mRNAs list					
Name	Identifier	Log_2 fold change(nonCAD minus CAD)	Fold change	P-value	FDR p-value
hsa-miR-206	URS000034B6F5_9606	-4,203973863	-18,42986839	6,485E-07	0,000
hsa-miR-9-5p	URS00004208C5_9606	-4,000785109	-16,00870951	5,589E-04	0,098
hsa-miR-199a-3p	URS00003F2D94_9606	-1,386774226	-2,614933442	2,285E-03	0,27
hsa-miR-184	URS0000543D82_9606	-2,834302174	-7,131977609	4,644E-03	0,41
hsa-miR-375-3p	URS00000ED600_9606	-2,103015427	-4,296063824	6,986E-03	NA
hsa-miR-137-3p	URS00001E3523_9606	-4,433006522	-21,60070541	8,083E-03	NA
hsa-miR-513a-3p	URS00004BCE9B_9606	-3,712667694	-13,11065351	9,767E-03	NA
hsa-miR-509-3p	URS00002E177F_9606	-2,040260598	-4,113198219	1,015E-02	0,62
hsa-miR-2355-3p	URS00003804D8_9606	-1,477811645	-2,785259301	1,068E-02	0,62
hsa-miR-873-3p	URS000026C425_9606	-2,147147001	-4,429509651	1,082E-02	NA
hsa-miR-136-5p	URS00004EAB18_9606	-1,82979221	-3,554858686	1,392E-02	NA
hsa-miR-3193	URS000075B0E9_9606	-2,415598543	-5,335407794	1,715E-02	NA
hsa-miR-548a-3p	URS000038037E_9606	-1,40725388	-2,652318224	1,920E-02	NA
hsa-miR-4516	URS00000BF7F9_9606	1,534504117	2,896888419	1,959E-02	0,70
hsa-miR-941	URS000050E4BA_9606	1,489530211	2,807975233	1,962E-02	0,70
hsa-miR-513b-3p	URS000075EDE8_9606	-3,205617334	-9,225437526	2,134E-02	NA
hsa-miR-34b-5p	URS0000432971_9606	-2,994296154	-7,968433601	2,134E-02	NA
hsa-miR-509-5p	URS000024AD66_9606	-2,528651746	-5,770321676	2,172E-02	NA
hsa-miR-548ay-3p	URS000075D0C5_9606	2,280417906	4,858186607	2,237E-02	NA
hsa-miR-345-5p	URS000005D4F5_9606	1,225371554	2,338156592	2,390E-02	0,70
hsa-miR-34c-3p	URS00002C7C59_9606	-1,584534613	-2,999110365	2,484E-02	0,70
hsa-miR-21-5p	URS000039ED8D_9606	-1,027620386	-2,038658866	2,710E-02	0,70
hsa-miR-193b-5p	URS00000E1DC5_9606	-1,167370102	-2,246018953	2,740E-02	0,70
hsa-miR-508-3p	URS000044FE6A_9606	-2,196941833	-4,585063877	2,862E-02	0,70
hsa-miR-6500-3p	URS000075BB5D_9606	-2,845532119	-7,187709583	2,879E-02	NA
hsa-miR-483-3p	URS00000EA063_9606	-2,861044334	-7,265410605	2,903E-02	NA
hsa-miR-146a-5p	URS000050B527_9606	-1,262536501	-2,399171851	2,918E-02	0,70
hsa-miR-509-3-5p	URS0000465F56_9606	-1,960601338	-3,8922418	3,386E-02	NA
hsa-miR-1285-3p	URS0000399545_9606	-2,010143612	-4,028223168	3,433E-02	NA
hsa-miR-1180-5p	URS00007769A4_9606	-3,394437885	-10,51544417	3,468E-02	NA
hsa-miR-514a-3p	URS00001AADF2_9606	-1,710916521	-3,273687294	3,488E-02	0,70
hsa-miR-513c-5p	URS00002A7CF3_9606	-1,945864708	-3,852686266	3,516E-02	0,70
hsa-miR-6827-3p	URS000075ACD6_9606	-2,50438194	-5,674062097	3,546E-02	NA
hsa-miR-200c-3p	URS0000192F9C_9606	1,289650805	2,444688763	3,575E-02	0,70
hsa-miR-377-3p	URS000007F792_9606	-1,892803944	-3,713562718	3,575E-02	NA

hsa-miR-191-5p	URS00005C2E31_9606	1,094324162	2,135130369	3,652E-02	0,70
hsa-miR-9-3p	URS00003496BE_9606	-2,144576746	-4,421625217	3,653E-02	NA
hsa-miR-12136	URS0000D53EF0_9606	-1,677523442	-3,1987837	3,740E-02	NA
hsa-miR-135b-3p	URS0000488C83_9606	-1,26172747	-2,397826828	3,920E-02	NA
hsa-miR-1246	URS000028C188_9606	2,052384718	4,147910364	3,923E-02	0,70
hsa-miR-6891-5p	URS000075BD73_9606	-2,189948594	-4,562892276	3,996E-02	NA
hsa-miR-1273h-3p	URS000075C34D_9606	-3,419830624	-10,70216391	4,026E-02	NA
hsa-miR-30c-5p	URS000019907A_9606	1,210743888	2,314569508	4,090E-02	0,70
hsa-miR-423-5p	URS00001C8A86_9606	-0,969251642	-1,957824765	4,185E-02	0,70
hsa-miR-4488	URS0000419B5A_9606	2,53578678	5,798930226	4,449E-02	NA
hsa-miR-513c-3p	URS00001A4ABF_9606	-2,413649846	-5,328205943	4,779E-02	NA
hsa-miR-5698	URS000075D0C4_9606	2,542129641	5,82448156	4,854E-02	NA
hsa-miR-491-5p	URS00001919B0_9606	1,420592923	2,676955067	4,892E-02	0,71
hsa-miR-3195	URS000004DB7E_9606	2,554665218	5,875310991	5,041E-02	0,71
hsa-miR-362-5p	URS0000085F64_9606	1,43241809	2,698987116	5,124E-02	0,71
hsa-let-7e-3p	URS0000409B45_9606	1,263599314	2,400939939	5,135E-02	NA
hsa-miR-935	URS000033EBB8_9606	2,271089182	4,826874048	5,190E-02	NA
hsa-miR-205-3p	URS000038727B_9606	-2,553426209	-5,870267349	5,269E-02	NA
hsa-miR-148a-3p	URS00003BBF48_9606	-0,789141523	-1,728045881	5,325E-02	0,71
hsa-miR-187-3p	URS00001EB9FC_9606	-1,447876667	-2,728062444	5,344E-02	NA
hsa-miR-15a-3p	URS00001C94E0_9606	-1,819528288	-3,529657719	5,382E-02	NA
hsa-miR-576-3p	URS000020CEC2_9606	-1,483485954	-2,796235663	5,396E-02	NA
hsa-miR-34c-5p	URS00002C7B2B_9606	-1,951603152	-3,868041175	5,709E-02	0,71
hsa-miR-181a-2-3p	URS0000241987_9606	0,943895774	1,923715928	5,714E-02	0,71
hsa-miR-4443	URS00004D84DB_9606	-0,999807006	-1,999732472	6,029E-02	0,71
hsa-miR-214-5p	URS00004DAA89_9606	-1,765069133	-3,398902866	6,139E-02	0,71
hsa-miR-510-3p	URS000075B179_9606	-3,037138586	-8,208613647	6,150E-02	NA
hsa-miR-141-5p	URS000055E199_9606	-1,377921246	-2,59893625	6,252E-02	0,71
hsa-miR-507	URS00005E6F45_9606	-2,556075938	-5,881058894	6,279E-02	NA
hsa-miR-486-5p	URS00004BF1DC_9606	-1,203916043	-2,303641222	6,281E-02	0,71
hsa-miR-1267	URS000075AEB2_9606	1,85498036	3,617468265	6,289E-02	NA
hsa-miR-202-3p	URS00003E24B2_9606	-1,173292563	-2,25525812	6,549E-02	NA
hsa-miR-148b-3p	URS0000521626_9606	-0,813541431	-1,757520395	6,660E-02	0,73
hsa-miR-4732-3p	URS00001A122A_9606	-2,834088212	-7,130919964	6,671E-02	NA
hsa-miR-3177-3p	URS00003677B2_9606	-1,734718989	-3,328146614	6,849E-02	NA
hsa-miR-383-5p	URS00000C5969_9606	-1,925092334	-3,797611528	7,201E-02	NA
hsa-miR-192-3p	URS00000B59A2_9606	1,462840378	2,756505305	7,278E-02	NA
hsa-miR-876-5p	URS0000470305_9606	-2,380455933	-5,207012727	7,331E-02	NA
hsa-miR-3616-3p	URS000075D1FC_9606	-2,380455933	-5,207012727	7,331E-02	NA

hsa-miR-182-5p	URS00001CC379_9606	1,284417595	2,435836999	7,436E-02	0,77
hsa-miR-124-3p	URS00000ADB79_9606	-1,927635729	-3,80431242	7,545E-02	NA
hsa-miR-15a-5p	URS00003D1AE3_9606	-0,971023555	-1,960230835	7,621E-02	0,77
hsa-miR-4742-3p	URS00000C48FA_9606	2,088304946	4,252481459	7,638E-02	NA
hsa-miR-5010-3p	URS000025C0E1_9606	1,599636645	3,030669736	7,824E-02	NA
hsa-miR-4749-5p	URS00000CDD4F_9606	-1,561222815	-2,951038647	8,314E-02	NA
hsa-miR-6784-3p	URS000075C32F_9606	-2,389624117	-5,240208142	8,386E-02	NA
hsa-miR-4640-5p	URS0000335C38_9606	2,087599445	4,250402436	8,413E-02	NA
hsa-miR-365a-3p	URS00003E7283_9606	0,79407987	1,73397112	8,519E-02	0,77
hsa-miR-4639-5p	URS00003F2CCA_9606	-2,730597146	-6,637303047	8,583E-02	NA
hsa-miR-4777-3p	URS00005E66C8_9606	-1,686949471	-3,21975178	8,667E-02	NA
hsa-miR-514a-5p	URS000042B7FA_9606	-1,619365917	-3,072399706	8,759E-02	NA
hsa-miR-549a-5p	URS0000D563D6_9606	-1,674190107	-3,19140147	9,101E-02	NA
hsa-miR-6716-3p	URS000075ED55_9606	1,823143165	3,538512867	9,120E-02	NA
hsa-miR-514b-5p	URS00003AA851_9606	-2,136899448	-4,398158047	9,278E-02	NA
hsa-miR-3934-5p	URS00003ACE11_9606	1,216089013	2,323160796	9,322E-02	NA
hsa-miR-376b-3p	URS00003AD231_9606	-1,430520817	-2,695440039	9,437E-02	NA
hsa-miR-99b-5p	URS00002C10B3_9606	0,747308481	1,678658171	9,507E-02	0,77
hsa-miR-582-5p	URS00000B0E50_9606	-1,637450827	-3,111156195	9,597E-02	NA
hsa-miR-1290	URS000043F369_9606	1,823483232	3,539347051	9,918E-02	NA
hsa-miR-192-5p	URS0000155642_9606	0,933540335	1,909957234	9,953E-02	0,77
hsa-miR-219a-1-3p	URS00000BD1DE_9606	1,193932889	2,287755523	1,004E-01	NA
hsa-miR-1291	URS000047E28E_9606	-1,544572562	-2,917176266	1,015E-01	NA
hsa-miR-3194-5p	URS000050F244_9606	-2,185200086	-4,547898594	1,038E-01	NA
hsa-miR-3085-3p	URS00003A42F5_9606	-2,185200086	-4,547898594	1,038E-01	NA
hsa-miR-1185-5p	URS00002C28B2_9606	-2,185200086	-4,547898594	1,038E-01	NA
hsa-miR-145-3p	URS000052F380_9606	-1,494533323	-2,817729895	1,040E-01	0,77
hsa-miR-627-5p	URS000040FA88_9606	-1,24396486	-2,368485533	1,052E-01	NA
hsa-miR-133a-3p	URS00004C9052_9606	-1,466809659	-2,764099709	1,062E-01	0,77
hsa-miR-3129-5p	URS00000F7766_9606	-0,988688413	-1,984380126	1,063E-01	NA
hsa-miR-223-3p	URS00000B7E30_9606	0,952588398	1,93534182	1,067E-01	0,77
hsa-miR-597-5p	URS000075BC35_9606	-1,31412485	-2,486514509	1,071E-01	NA
hsa-miR-514b-3p	URS000002569A_9606	-1,7048709	-3,259997594	1,086E-01	NA
hsa-miR-23a-5p	URS00005070A9_9606	-1,315255361	-2,488463732	0,11	NA
hsa-miR-203a-3p	URS00004DA9DB_9606	0,762487073	1,696412564	0,11	0,77
hsa-miR-411-5p	URS00000C5BAA_9606	-1,093606945	-2,13406918	0,11	0,77
hsa-miR-873-5p	URS00004996E9_9606	-1,793468192	-3,466472202	0,11	0,77
hsa-miR-23b-5p	URS000055BB1F_9606	-0,958543931	-1,943347542	0,12	0,77
hsa-miR-548i	URS00004C1D89_9606	-1,344747591	-2,539857561	0,12	NA

hsa-miR-3180	URS00005FE063_9606	1,877984498	3,675612039	0,12	NA
hsa-miR-10b-5p	URS000058760A_9606	-0,960965966	-1,946612826	0,12	0,77
hsa-miR-1288-3p	URS000075CAED_9606	-1,421590369	-2,678806491	0,12	NA
hsa-miR-513a-5p	URS0000357286_9606	-2,202781649	-4,60366115	0,12	NA
hsa-miR-188-3p	URS000061AB33_9606	-1,541403895	-2,910776149	0,12	NA
hsa-miR-335-3p	URS00005092C2_9606	0,724132156	1,651906643	0,13	0,77
hsa-miR-190a-3p	URS0000759F1D_9606	1,612979976	3,058830101	0,13	NA
hsa-miR-125a-5p	URS00005A4DCF_9606	0,931608636	1,907401605	0,13	0,77
hsa-miR-10b-3p	URS00004AC389_9606	-1,043790041	-2,061636581	0,13	0,77
hsa-miR-382-3p	URS000013E79D_9606	-1,114709002	-2,165513254	0,13	NA
hsa-miR-143-3p	URS00005C2A6D_9606	-0,899476142	-1,865388518	0,13	0,77
hsa-miR-151b	URS00003E6479_9606	-1,601103486	-3,033752698	0,13	NA
hsa-miR-548f-3p	URS00000B7F84_9606	-1,600316101	-3,032097406	0,13	NA
hsa-miR-1287-3p	URS0000759A79_9606	-1,600184232	-3,031820271	0,13	NA
hsa-miR-4757-3p	URS00000F339A_9606	-1,600189758	-3,031831884	0,13	NA
hsa-miR-1270	URS00002E0524_9606	1,247490339	2,374280421	0,13	0,77
hsa-miR-486-3p	URS0000377E71_9606	1,425611188	2,68628279	0,13	0,77
hsa-miR-200c-5p	URS0000346F1C_9606	-0,971032637	-1,960243176	0,14	NA
hsa-miR-4775	URS000005542C_9606	-1,431591923	-2,69744197	0,14	NA
hsa-miR-513b-5p	URS0000284586_9606	-1,906952068	-3,750159789	0,14	NA
hsa-miR-143-5p	URS00003E93AB_9606	-1,644186763	-3,125716131	0,14	0,77
hsa-miR-6515-3p	URS000075BDA4_9606	1,878116084	3,675947302	0,14	NA
hsa-miR-339-5p	URS000003FD55_9606	1,032962128	2,046221224	0,14	0,77
hsa-miR-155-5p	URS0000D54CAD_9606	-1,082575966	-2,117814114	0,14	0,77
hsa-miR-146a-3p	URS00001EFBE3_9606	1,878558474	3,677074672	0,15	NA
hsa-miR-183-5p	URS0000528CBC_9606	0,987019616	1,982086077	0,15	0,77
hsa-miR-146b-5p	URS000075DF95_9606	0,737929485	1,667780569	0,15	0,77
hsa-miR-497-3p	URS000007D64A_9606	-0,876626903	-1,836077433	0,15	NA
hsa-miR-30d-5p	URS000005CF5F_9606	0,759529685	1,692938641	0,15	0,77
hsa-miR-450a-1-3p	URS000040BEF4_9606	1,477012894	2,783717663	0,15	NA
hsa-miR-26a-5p	URS000019B0F7_9606	0,663959519	1,584425164	0,15	0,77
hsa-miR-299-3p	URS00003B1F5C_9606	-1,600316101	-3,032097406	0,15	NA
hsa-miR-129-5p	URS00004E1410_9606	-1,018380586	-2,025643914	0,15	NA
hsa-miR-4665-5p	URS00000E9F44_9606	-1,187617466	-2,27776272	0,15	NA
hsa-miR-224-3p	URS00001E8884_9606	-1,960313417	-3,891465095	0,15	NA
hsa-miR-561-5p	URS000040828B_9606	-1,187485732	-2,277554745	0,15	NA
hsa-miR-4296	URS000075CE36_9606	-1,960159651	-3,891050356	0,15	NA
hsa-miR-944	URS000004203C_9606	-1,960159651	-3,891050356	0,15	NA
hsa-miR-6884-5p	URS0000759FCF_9606	-1,960159651	-3,891050356	0,15	NA

hsa-miR-5000-3p	URS000075BECA_9606	-1,960159651	-3,891050356	0,15	NA
hsa-miR-495-5p	URS00004FFC5A_9606	-1,959245693	-3,888586128	0,15	NA
hsa-miR-301b-3p	URS0000251D0B_9606	-1,959245693	-3,888586128	0,15	NA
hsa-miR-603	URS000075A6F1_9606	-1,959245693	-3,888586128	0,15	NA
hsa-miR-6828-5p	URS000075BAA7_9606	-1,959245693	-3,888586128	0,15	NA
hsa-miR-122b-5p	URS000052829C_9606	-1,959245693	-3,888586128	0,15	NA
hsa-miR-4667-5p	URS00003CEC17_9606	-1,959245693	-3,888586128	0,15	NA
hsa-miR-636	URS000075A79D_9606	-1,961228053	-3,89393298	0,15	NA
hsa-miR-32-5p	URS00004C47FB_9606	-0,701656569	-1,626371195	0,15	0,77
hsa-miR-17-3p	URS00004636A3_9606	0,857377167	1,811741551	0,15	NA
hsa-miR-506-3p	URS00004AE8D3_9606	-1,573298801	-2,975843797	0,15	0,77
hsa-miR-186-5p	URS000040DCFF_9606	0,685099303	1,607812646	0,15	0,77
hsa-miR-4710	URS000023F008_9606	-0,84430929	-1,795404966	0,15	NA
hsa-miR-574-3p	URS00001CF056_9606	0,922825969	1,895825224	0,16	0,77
hsa-miR-452-5p	URS0000550C66_9606	-0,877558621	-1,837263587	0,16	0,77
hsa-miR-1180-3p	URS0000079D48_9606	-0,793455793	-1,733221206	0,16	0,77
hsa-miR-205-5p	URS0000446722_9606	-1,05337053	-2,075372827	0,17	0,77
hsa-miR-508-5p	URS000021202F_9606	-1,13196715	-2,19157363	0,17	0,77
hsa-miR-328-3p	URS00005FDE70_9606	-1,042473131	-2,059755552	0,17	0,77
hsa-miR-23b-3p	URS00004E57E7_9606	0,87785282	1,837638287	0,17	0,77
hsa-miR-4466	URS00001DC1D3_9606	-1,962242177	-3,896671132	0,17	NA
hsa-miR-6511b-5p	URS0000759A05_9606	-1,267137724	-2,406835805	0,17	NA
hsa-miR-744-3p	URS00005FAA14_9606	-1,267137724	-2,406835805	0,17	NA
hsa-miR-30b-5p	URS00005165DA_9606	0,755183888	1,687846714	0,17	0,77
hsa-miR-4705	URS00004FE139_9606	-1,26805048	-2,40835903	0,17	NA
hsa-miR-500a-5p	URS000039A052_9606	0,825100658	1,771658644	0,17	0,77
hsa-miR-324-5p	URS000075BEBE_9606	0,838518782	1,788213237	0,18	0,77
hsa-miR-126-5p	URS00001D69F6_9606	-0,824528916	-1,770956673	0,18	0,77
hsa-miR-103a-2-5p	URS000060A0B0_9606	-0,996711683	-1,995446616	0,18	NA
hsa-miR-130a-3p	URS0000315338_9606	-0,629005364	-1,546498425	0,18	0,77
hsa-miR-4670-3p	URS00005CC60E_9606	1,608390929	3,049115772	0,18	NA
hsa-miR-4660	URS00002768C0_9606	-1,133344496	-2,193666933	0,18	NA
hsa-miR-199a-5p	URS0000554A4F_9606	-1,006842403	-2,009508114	0,18	0,77
hsa-let-7e-5p	URS000000B1C9_9606	0,629696522	1,54723949	0,18	0,77
hsa-miR-152-3p	URS00003AFD9B_9606	-0,567078052	-1,481519948	0,18	0,77
hsa-miR-4762-3p	URS0000437512_9606	1,633320056	3,102260974	0,18	NA
hsa-miR-4697-3p	URS00003685CF_9606	1,632745987	3,101026785	0,18	NA
hsa-miR-103a-3p	URS0000476BE1_9606	0,687413987	1,610394318	0,18	0,77
hsa-miR-585-3p	URS000054D341_9606	1,333009831	2,519277127	0,18	0,77

hsa-miR-361-3p	URS000031E6A1_9606	0,931428284	1,907163175	0,18	0,77
hsa-miR-374b-5p	URS000033F45D_9606	0,729455492	1,658013199	0,18	0,77
hsa-miR-454-3p	URS00004F77ED_9606	0,914255349	1,884596082	0,18	0,77
hsa-miR-484	URS0000597BED_9606	-1,020458111	-2,028563003	0,18	0,77
hsa-miR-181b-3p	URS0000229622_9606	-1,150254616	-2,219530626	0,18	NA
hsa-miR-664a-5p	URS0000259AE4_9606	-0,708847218	-1,634497554	0,18	0,77
hsa-miR-193a-5p	URS0000367985_9606	-0,781490483	-1,718905799	0,19	0,77
hsa-miR-3934-3p	URS000075B5A4_9606	-1,55953012	-2,947578263	0,19	NA
hsa-miR-2355-5p	URS0000609C67_9606	-1,558022403	-2,944499449	0,19	NA
hsa-miR-891b	URS000075BCBB_9606	-1,135193625	-2,196480398	0,19	NA
hsa-miR-660-5p	URS0000116A70_9606	-0,715589657	-1,642154261	0,19	0,77
hsa-miR-4668-5p	URS00000A17E7_9606	-1,267476382	-2,407400852	0,19	NA
hsa-miR-548e-3p	URS00000D2680_9606	-0,954063312	-1,937321396	0,20	NA
hsa-miR-584-5p	URS0000576F83_9606	-0,781931697	-1,719431566	0,20	0,79
hsa-miR-6871-5p	URS000075E950_9606	-1,404934312	-2,648057248	0,20	NA
hsa-miR-6729-5p	URS000075DD20_9606	-1,404802443	-2,647815214	0,20	NA
hsa-miR-1286	URS00002EF20A_9606	-1,404020756	-2,646380953	0,20	NA
hsa-miR-374a-5p	URS000029E173_9606	0,664670378	1,58520605	0,20	0,81
hsa-miR-510-5p	URS00001CC4BD_9606	-1,420546201	-2,676868375	0,20	NA
hsa-miR-212-5p	URS00001AFC71_9606	1,208699677	2,311292225	0,20	NA
hsa-miR-548az-5p	URS000075B410_9606	-0,997354846	-1,996336398	0,21	NA
hsa-miR-215-5p	URS0000315B13_9606	0,969698427	1,958431172	0,21	NA
hsa-miR-30a-5p	URS000043D1A9_9606	0,552742338	1,466871341	0,21	0,81
hsa-miR-30c-2-3p	URS0000527228_9606	0,82787846	1,775073127	0,21	0,81
hsa-miR-1-3p	URS00001DC04F_9606	-1,133489726	-2,19388777	0,21	0,81
hsa-miR-365a-5p	URS000031B6ED_9606	-1,051004702	-2,07197228	0,21	NA
hsa-miR-431-3p	URS0000086A4D_9606	-1,313344993	-2,485170774	0,21	NA
hsa-miR-615-3p	URS00003D5391_9606	-1,629953987	-3,095031273	0,21	NA
hsa-miR-3200-5p	URS00001938D9_9606	1,266692222	2,406092692	0,22	NA
hsa-miR-146b-3p	URS0000D52743_9606	0,715611581	1,642179215	0,22	0,82
hsa-miR-3661	URS00002CCA6E_9606	1,266129738	2,405154778	0,22	NA
hsa-miR-101-5p	URS000017D10B_9606	-0,760739368	-1,694358746	0,22	NA
hsa-miR-150-5p	URS000016FD1A_9606	-1,083583675	-2,119293905	0,22	0,82
hsa-miR-296-3p	URS00001F4670_9606	-0,692847079	-1,616470388	0,22	0,82
hsa-miR-3685	URS0000759D24_9606	-1,693230459	-3,233800007	0,22	NA
hsa-miR-6869-5p	URS000075C3FC_9606	-1,692950296	-3,233172084	0,22	NA
hsa-miR-1234-3p	URS000075E194_9606	-1,692950296	-3,233172084	0,22	NA
hsa-miR-6885-3p	URS000075B6DE_9606	-1,692189072	-3,231466582	0,22	NA
hsa-miR-6815-3p	URS000075B15F_9606	-1,692189072	-3,231466582	0,22	NA

hsa-miR-6894-5p	URS0000759A75_9606	-1,69216159	-3,231405027	0,22	NA
hsa-miR-548ae-3p	URS00003257B1_9606	-1,692035352	-3,231122286	0,22	NA
hsa-miR-11401	URS0000D56AF1_9606	-1,692035352	-3,231122286	0,22	NA
hsa-miR-655-3p	URS00005E1A16_9606	-1,691120992	-3,229075093	0,22	NA
hsa-miR-6777-5p	URS000075B8A2_9606	-1,691120992	-3,229075093	0,22	NA
hsa-miR-6889-5p	URS000075C76B_9606	-1,691120992	-3,229075093	0,22	NA
hsa-miR-758-3p	URS000024B619_9606	-1,691120992	-3,229075093	0,22	NA
hsa-miR-6748-5p	URS000075ECB7_9606	-1,691120992	-3,229075093	0,22	NA
hsa-miR-3116	URS000060E0A6_9606	-1,691120992	-3,229075093	0,22	NA
hsa-miR-4733-5p	URS0000589066_9606	-1,691120992	-3,229075093	0,22	NA
hsa-miR-3186-3p	URS00004CFC54_9606	-1,691120992	-3,229075093	0,22	NA
hsa-miR-7851-3p	URS000075DAFC_9606	-1,691120992	-3,229075093	0,22	NA
hsa-miR-1236-3p	URS000075D8A7_9606	-1,691120992	-3,229075093	0,22	NA
hsa-miR-888-3p	URS0000759E8F_9606	-1,691120992	-3,229075093	0,22	NA
hsa-miR-585-5p	URS000077ACC0_9606	-0,807608785	-1,750307965	0,22	NA
hsa-miR-151a-3p	URS000016C318_9606	0,539030935	1,452996205	0,23	0,82
hsa-let-7i-5p	URS00004023EA_9606	-0,529340436	-1,443269218	0,23	0,82
hsa-miR-6875-5p	URS000075DAC6_9606	-1,268642635	-2,409347745	0,23	NA
hsa-miR-138-1-3p	URS00005517FD_9606	1,063568975	2,090095662	0,23	NA
hsa-miR-147b-5p	URS0000D535C2_9606	-1,692950296	-3,233172084	0,23	NA
hsa-miR-29c-3p	URS0000272A3D_9606	-0,634374235	-1,552264314	0,23	0,82
hsa-miR-153-3p	URS0000068B85_9606	-1,299897062	-2,462113146	0,23	NA
hsa-miR-33a-5p	URS0000483184_9606	-0,801400909	-1,74279262	0,23	NA
hsa-miR-1297	URS00004AF715_9606	-0,883954107	-1,84542628	0,23	NA
hsa-miR-549a-3p	URS00004C689A_9606	-1,141381626	-2,205921766	0,23	0,82
hsa-miR-6805-5p	URS000075CC09_9606	-1,013584906	-2,018921625	0,23	NA
hsa-miR-214-3p	URS00002C11C3_9606	-0,994995363	-1,99307412	0,24	0,82
hsa-miR-532-3p	URS00004B4B85_9606	-0,685515732	-1,608276803	0,24	0,82
hsa-miR-676-3p	URS00004407DC_9606	-0,665675711	-1,586311076	0,24	0,82
hsa-miR-29a-3p	URS00002F4D78_9606	-0,643865244	-1,562509806	0,24	0,82
hsa-miR-324-3p	URS0000D50706_9606	-0,570071667	-1,484597317	0,24	0,82
hsa-miR-6842-5p	URS000075A33B_9606	-1,095102299	-2,136282291	0,24	NA
hsa-miR-1296-5p	URS000002103A_9606	0,808278379	1,751120519	0,25	NA
hsa-miR-125b-5p	URS0000209905_9606	0,577722242	1,49249101	0,25	0,82
hsa-miR-1247-5p	URS000057DF36_9606	-1,450007663	-2,732095025	0,25	NA
hsa-miR-455-5p	URS00000AD002_9606	0,705453561	1,630657237	0,25	0,82
hsa-miR-320d	URS0000010C72_9606	-0,58057855	-1,495448834	0,25	0,82
hsa-let-7c-5p	URS000050DE77_9606	0,539246432	1,453213257	0,25	0,82
hsa-miR-382-5p	URS000035E174_9606	-1,078679838	-2,112102482	0,25	0,82

hsa-miR-542-5p	URS000050C722_9606	0,789145524	1,728050674	0,25	NA
hsa-miR-27b-5p	URS0000330617_9606	0,708889683	1,634545665	0,25	NA
hsa-miR-204-5p	URS000029D9F1_9606	0,989321883	1,985251635	0,26	0,82
hsa-miR-551a	URS00002E99CB_9606	0,658835298	1,578807523	0,26	0,82
hsa-miR-320c	URS0000010D30_9606	-0,687004569	-1,609937374	0,26	0,82
hsa-miR-494-3p	URS0000535FDD_9606	-1,023006492	-2,032149431	0,26	NA
hsa-miR-425-5p	URS000048BA36_9606	0,662759532	1,583107838	0,27	0,83
hsa-miR-582-3p	URS00002573C3_9606	-0,974135937	-1,964464282	0,27	0,84
hsa-miR-497-5p	URS00001BC212_9606	-0,640757348	-1,559147425	0,27	0,84
hsa-miR-3198	URS000012568D_9606	-1,098255895	-2,140957111	0,27	NA
hsa-miR-4999-5p	URS000075CA38_9606	0,771655769	1,707228031	0,27	NA
hsa-miR-323b-3p	URS00003538C1_9606	1,061906643	2,087688756	0,27	NA
hsa-miR-4492	URS000045ED38_9606	-1,269903279	-2,411453981	0,27	NA
hsa-miR-5699-5p	URS000075E5A8_9606	-1,069949089	-2,099359282	0,27	NA
hsa-miR-101-3p	URS00001230A0_9606	-0,454356722	-1,370171728	0,28	0,84
hsa-miR-31-5p	URS00005416E3_9606	0,669879612	1,590940203	0,28	0,84
hsa-miR-2116-5p	URS00000BA274_9606	-0,772616217	-1,708364965	0,28	NA
hsa-miR-3059-3p	URS00000AEAF5_9606	-0,819115591	-1,764324085	0,28	NA
hsa-miR-181a-3p	URS000003F252_9606	0,571294487	1,485856187	0,28	0,84
hsa-miR-99a-3p	URS00005C62FC_9606	0,560993269	1,475284574	0,28	0,84
hsa-miR-4497	URS00000A2C49_9606	-0,734868007	-1,6642452	0,28	NA
hsa-miR-3667-5p	URS000075E8D3_9606	-1,365172743	-2,576071691	0,29	NA
hsa-miR-186-3p	URS000021D7C8_9606	-0,705719232	-1,630957549	0,29	NA
hsa-miR-3141	URS00002F72ED_9606	1,337444894	2,527033682	0,29	NA
hsa-miR-5187-3p	URS000075CB70_9606	1,337433356	2,527013473	0,29	NA
hsa-miR-3150a-5p	URS00004D5A7A_9606	1,337301571	2,52678265	0,29	NA
hsa-miR-6810-5p	URS000075C62C_9606	1,336870784	2,526028268	0,29	NA
hsa-miR-6852-5p	URS000075E060_9606	1,336870784	2,526028268	0,29	NA
hsa-miR-450a-5p	URS00003E5ECC_9606	1,336870784	2,526028268	0,29	NA
hsa-miR-7111-3p	URS000075B456_9606	1,33673905	2,525797625	0,29	NA
hsa-miR-6511a-3p	URS000075E460_9606	1,33673905	2,525797625	0,29	NA
hsa-miR-12135	URS0000D52303_9606	1,33673905	2,525797625	0,29	NA
hsa-miR-4469	URS00002CEB74_9606	1,336607329	2,525567024	0,29	NA
hsa-miR-3194-3p	URS000048D44E_9606	1,336607329	2,525567024	0,29	NA
hsa-miR-6882-5p	URS000075C680_9606	-1,178848452	-2,263959973	0,29	NA
hsa-miR-5588-5p	URS00000EBD4B_9606	-1,178066363	-2,262733007	0,29	NA
hsa-miR-6779-5p	URS000075A236_9606	-1,178066363	-2,262733007	0,29	NA
hsa-miR-6807-5p	URS000075EA7D_9606	-1,178066363	-2,262733007	0,29	NA
hsa-miR-12113	URS0000D54614_9606	1,337002531	2,526258955	0,29	NA

hsa-miR-144-5p	URS00002E92A8_9606	0,785728402	1,72396251	0,29	0,84
hsa-miR-33b-5p	URS00004C8DD5_9606	-0,894313165	-1,858724777	0,29	NA
hsa-miR-200b-5p	URS000012A1DD_9606	-0,596359926	-1,511897072	0,29	0,84
hsa-miR-21-3p	URS000009262D_9606	-0,849304654	-1,80163237	0,29	0,84
hsa-miR-6763-5p	URS0000759FD0_9606	-0,895357655	-1,860070953	0,29	NA
hsa-miR-1468-5p	URS00002ECEE4_9606	-1,098002899	-2,140581698	0,29	NA
hsa-miR-365b-5p	URS00003E2232_9606	0,784740438	1,722782338	0,30	0,84
hsa-miR-30e-3p	URS00004DC6A5_9606	0,564376128	1,478747909	0,30	0,84
hsa-miR-409-5p	URS0000081E1F_9606	-0,923103243	-1,896189621	0,30	NA
hsa-miR-6868-3p	URS000075DF84_9606	-1,227491224	-2,341594438	0,30	NA
hsa-miR-376c-3p	URS00005E651E_9606	-1,067659799	-2,096030629	0,30	0,84
hsa-let-7c-3p	URS000060A34C_9606	0,987371159	1,982569112	0,30	NA
hsa-miR-6516-5p	URS000075A0B9_9606	-1,098410002	-2,141185817	0,30	NA
hsa-miR-378e	URS00000B1B47_9606	0,796196338	1,736516764	0,30	NA
hsa-miR-6788-3p	URS00007E488C_9606	-1,504311342	-2,836892224	0,30	NA
hsa-miR-651-5p	URS000049FAE8_9606	-0,635685178	-1,55367546	0,30	0,84
hsa-let-7g-3p	URS000029979D_9606	-0,724122124	-1,651895157	0,30	NA
hsa-miR-190a-5p	URS0000520927_9606	0,697378544	1,621555659	0,30	0,84
hsa-miR-26b-5p	URS0000316FA5_9606	0,555351679	1,469526809	0,30	0,84
hsa-miR-193a-3p	URS00005DBAF3_9606	-1,035082179	-2,049230371	0,31	0,86
hsa-miR-885-3p	URS00003F4546_9606	-1,691120992	-3,229075093	0,31	NA
hsa-miR-31-3p	URS00002A291B_9606	-0,579272374	-1,494095509	0,31	0,86
hsa-miR-1179	URS000048B5E9_9606	-0,649260788	-1,568364388	0,32	NA
hsa-miR-891a-5p	URS000075B0C2_9606	-0,880256321	-1,840702306	0,32	NA
hsa-miR-98-3p	URS0000122ED0_9606	0,761272438	1,69498492	0,32	NA
hsa-miR-376a-3p	URS000041E11D_9606	-0,826636817	-1,773546083	0,32	0,87
hsa-miR-487b-3p	URS000015B295_9606	-0,577965929	-1,49274313	0,32	NA
hsa-miR-6764-5p	URS000075AAD9_9606	0,794953907	1,73502194	0,32	NA
hsa-miR-874-5p	URS000073891E_9606	0,641433975	1,559878839	0,32	NA
hsa-miR-25-5p	URS00001A9746_9606	0,595200805	1,510682839	0,33	0,87
hsa-miR-1303	URS000032FC1A_9606	-0,753833238	-1,686267293	0,33	NA
hsa-miR-499a-5p	URS00000C7662_9606	-0,667213135	-1,588002446	0,33	0,88
hsa-miR-337-5p	URS0000306C70_9606	-1,157846139	-2,231240673	0,33	NA
hsa-miR-659-5p	URS000054DF42_9606	1,021465434	2,029979888	0,33	NA
hsa-miR-4709-5p	URS00005452E1_9606	1,020418541	2,028507366	0,33	NA
hsa-miR-6786-3p	URS000075E834_9606	1,020627981	2,028801871	0,33	NA
hsa-miR-4800-5p	URS0000539562_9606	1,020280831	2,028313747	0,33	NA
hsa-miR-1343-5p	URS0000759B67_9606	-1,363368405	-2,572851883	0,34	NA
hsa-miR-4482-5p	URS000075A962_9606	-1,363368404	-2,572851882	0,34	NA

hsa-miR-6750-5p	URS000075DA77_9606	-1,362480432	-2,57126879	0,34	NA
hsa-miR-6791-5p	URS0000759CCF_9606	-1,36245296	-2,571219829	0,34	NA
hsa-miR-6754-5p	URS000075A250_9606	-1,36245296	-2,571219829	0,34	NA
hsa-miR-6855-3p	URS000075C1AC_9606	-1,362326757	-2,570994915	0,34	NA
hsa-miR-4804-3p	URS00000D41D9_9606	-1,362326757	-2,570994915	0,34	NA
hsa-miR-504-3p	URS000075E5C3_9606	-1,362326757	-2,570994915	0,34	NA
hsa-miR-6726-3p	URS000075C89D_9606	-1,362326757	-2,570994915	0,34	NA
hsa-miR-532-5p	URS00004E8341_9606	0,535181119	1,44912407	0,34	0,88
hsa-miR-3691-5p	URS00000393C7_9606	-1,361411992	-2,56936525	0,34	NA
hsa-miR-6879-3p	URS000075E732_9606	-1,361411992	-2,56936525	0,34	NA
hsa-miR-543	URS000019F055_9606	-1,361411992	-2,56936525	0,34	NA
hsa-miR-4753-5p	URS00004B338E_9606	-1,361411992	-2,56936525	0,34	NA
hsa-miR-495-3p	URS000005A8ED_9606	-1,361411992	-2,56936525	0,34	NA
hsa-miR-6733-5p	URS000075EA52_9606	-1,361411992	-2,56936525	0,34	NA
hsa-miR-5702	URS000075EAC4_9606	-1,361411992	-2,56936525	0,34	NA
hsa-miR-4778-3p	URS00000DEF85_9606	-1,361411992	-2,56936525	0,34	NA
hsa-miR-4489	URS00003EB4CF_9606	-1,361411992	-2,56936525	0,34	NA
hsa-miR-3192-5p	URS00000AB32B_9606	-1,361411992	-2,56936525	0,34	NA
hsa-miR-10397-5p	URS0000D51422_9606	-1,361411992	-2,56936525	0,34	NA
hsa-miR-380-5p	URS000075BE5F_9606	-1,361411992	-2,56936525	0,34	NA
hsa-miR-885-5p	URS0000246356_9606	-1,361411992	-2,56936525	0,34	NA
hsa-miR-7109-3p	URS000075A43F_9606	-1,361411992	-2,56936525	0,34	NA
hsa-miR-5697	URS000075CD80_9606	-1,361411992	-2,56936525	0,34	NA
hsa-miR-517a-3p	URS00000D4AB5_9606	-1,361411992	-2,56936525	0,34	NA
hsa-miR-4684-3p	URS000052AB63_9606	-1,361411992	-2,56936525	0,34	NA
hsa-miR-6815-5p	URS000075A1BA_9606	-1,361411992	-2,56936525	0,34	NA
hsa-miR-892c-3p	URS000075EC6F_9606	-1,361411992	-2,56936525	0,34	NA
hsa-miR-6854-5p	URS000075ADDF_9606	-1,361411992	-2,56936525	0,34	NA
hsa-miR-6865-5p	URS000075A293_9606	-1,361411992	-2,56936525	0,34	NA
hsa-miR-6510-5p	URS000075A608_9606	-1,361411992	-2,56936525	0,34	NA
hsa-miR-6822-5p	URS00007599EF_9606	-1,361411992	-2,56936525	0,34	NA
hsa-miR-490-5p	URS00004556E5_9606	-1,361411992	-2,56936525	0,34	NA
hsa-miR-7156-3p	URS000075BA51_9606	-1,361411992	-2,56936525	0,34	NA
hsa-miR-1273h-5p	URS000075DA61_9606	-1,361411992	-2,56936525	0,34	NA
hsa-miR-758-5p	URS0000457089_9606	-1,361411992	-2,56936525	0,34	NA
hsa-miR-3130-3p	URS00002CA53E_9606	-1,361411992	-2,56936525	0,34	NA
hsa-miR-342-3p	URS0000148B91_9606	0,49427633	1,408614003	0,34	0,88
hsa-miR-19b-3p	URS000013D17D_9606	-0,653463354	-1,572939688	0,34	0,88
hsa-let-7f-5p	URS00003B7674_9606	0,496849533	1,411128661	0,34	0,88

hsa-miR-551b-3p	URS000008C563_9606	-0,882813193	-1,843967455	0,34	NA
hsa-miR-152-5p	URS000000C73C_9606	-1,040433595	-2,056845736	0,34	NA
hsa-miR-6761-5p	URS0000759C5F_9606	-0,901177421	-1,86758955	0,35	NA
hsa-miR-548c-3p	URS0000614A9B_9606	-0,90102361	-1,86739045	0,35	NA
hsa-miR-6735-5p	URS000075B9AD_9606	-0,900760008	-1,867049281	0,35	NA
hsa-miR-3136-5p	URS00000AF45F_9606	-0,900110055	-1,86620834	0,35	NA
hsa-miR-18a-5p	URS000035CC3E_9606	0,813721774	1,757740106	0,35	NA
hsa-miR-188-5p	URS0000083D87_9606	-0,555110782	-1,469281453	0,35	0,88
hsa-miR-1268b	URS000033B91D_9606	-0,90032925	-1,866491903	0,35	NA
hsa-miR-6842-3p	URS000075B6FC_9606	-0,481751928	-1,396438394	0,35	0,88
hsa-miR-130a-5p	URS0000229ECD_9606	0,901143792	1,867546017	0,35	NA
hsa-miR-93-5p	URS0000149452_9606	0,463566992	1,378946985	0,35	0,88
hsa-miR-19a-3p	URS000006FDD4_9606	-0,686034512	-1,608855228	0,36	0,88
hsa-miR-548l	URS0000260A3A_9606	-0,556351122	-1,470545193	0,36	NA
hsa-miR-337-3p	URS0000564D66_9606	-0,902064131	-1,868737762	0,36	NA
hsa-miR-485-3p	URS000006372A_9606	1,417879103	2,671924242	0,36	NA
hsa-miR-30e-5p	URS00001DE669_9606	0,476238954	1,391112363	0,36	0,88
hsa-miR-1271-5p	URS00001F61BA_9606	0,645863943	1,564675996	0,36	0,88
hsa-miR-3913-5p	URS000042A173_9606	-0,535994942	-1,449941749	0,36	NA
hsa-miR-1538	URS00005235AA_9606	-1,363242113	-2,572626668	0,36	NA
hsa-miR-4649-3p	URS00001C0099_9606	-1,363242113	-2,572626668	0,36	NA
hsa-miR-548j-5p	URS000008AA34_9606	0,606134881	1,522175686	0,36	NA
hsa-miR-502-5p	URS0000544FF1_9606	0,71540359	1,641942483	0,36	NA
hsa-miR-889-3p	URS000045B99E_9606	-1,259238933	-2,393694328	0,37	NA
hsa-miR-3921	URS000075F10E_9606	0,838011293	1,787584317	0,37	NA
hsa-miR-3157-5p	URS00000D5FFC_9606	0,838011293	1,787584317	0,37	NA
hsa-miR-222-5p	URS0000153377_9606	0,837879695	1,787421267	0,37	NA
hsa-miR-4520-2-3p	URS0000486E88_9606	0,837748108	1,787258246	0,37	NA
hsa-miR-4735-5p	URS000034435A_9606	0,83696493	1,786288282	0,37	NA
hsa-miR-9899	URS0000D4F559_9606	-1,363494681	-2,57307709	0,37	NA
hsa-miR-4750-5p	URS0000210075_9606	-0,754056527	-1,6865283	0,37	NA
hsa-miR-384	URS000075DD0E_9606	-1,363549706	-2,57317523	0,37	NA
hsa-miR-135b-5p	URS000001C659_9606	0,547282391	1,46133039	0,37	0,89
hsa-miR-6734-3p	URS000075EB5C_9606	-1,363549706	-2,57317523	0,37	NA
hsa-miR-369-3p	URS0000442B0D_9606	-0,930845769	-1,906393276	0,37	NA
hsa-miR-6820-3p	URS000075B26F_9606	-1,363549706	-2,57317523	0,37	NA
hsa-miR-542-3p	URS00004F859B_9606	0,588056606	1,503220458	0,37	0,89
hsa-miR-1237-3p	URS000075A763_9606	-1,363522177	-2,573126129	0,37	NA
hsa-miR-576-5p	URS000035E9A9_9606	0,65323852	1,572694576	0,38	0,89

hsa-miR-130b-3p	URS00002C0FCB_9606	-0,846228081	-1,797794451	0,38	0,89
hsa-miR-199b-5p	URS0000029EBD_9606	-0,833918667	-1,782520498	0,38	0,89
hsa-miR-675-3p	URS000027464B_9606	-0,819257057	-1,764497097	0,38	NA
hsa-miR-1299	URS00002F74F1_9606	0,84852721	1,800661762	0,38	0,89
hsa-miR-4448	URS00005F305A_9606	0,79446009	1,734428166	0,38	NA
hsa-miR-3684	URS0000759FC1_9606	1,045268673	2,063750655	0,38	NA
hsa-miR-485-5p	URS00001935FA_9606	-0,937983239	-1,915848178	0,38	0,89
hsa-miR-16-5p	URS00004BCD9C_9606	-0,45189593	-1,36783663	0,38	0,89
hsa-miR-548q	URS0000258D51_9606	-0,605982352	-1,522014763	0,38	NA
hsa-miR-877-3p	URS0000070B1D_9606	-1,365119899	-2,575977334	0,38	NA
hsa-let-7a-5p	URS0000416056_9606	0,421753922	1,339555097	0,39	0,89
hsa-miR-4787-3p	URS00002CBC6D_9606	0,759644311	1,693073155	0,39	NA
hsa-miR-125b-1-3p	URS00002DABEA_9606	-0,434201626	-1,351162905	0,39	0,89
hsa-miR-4433b-5p	URS000075A912_9606	1,568061061	2,965059511	0,39	NA
hsa-miR-548au-5p	URS000075CAF3_9606	-0,558226725	-1,472458246	0,39	NA
hsa-miR-93-3p	URS00000FB1B1_9606	0,520645438	1,434596919	0,39	0,89
hsa-miR-378a-5p	URS00004BAA8A_9606	0,47380627	1,388768635	0,39	0,89
hsa-miR-7704	URS000028F729_9606	0,752857424	1,685127116	0,40	NA
hsa-miR-92a-3p	URS00003768C5_9606	-0,468529199	-1,383698094	0,40	0,89
hsa-miR-548h-3p	URS000015188E_9606	-0,559276421	-1,473529988	0,40	0,89
hsa-miR-6862-5p	URS000075DA38_9606	-0,753666668	-1,686072611	0,40	NA
hsa-miR-6724-5p	URS00007777B8_9606	-0,491764229	-1,40616338	0,40	0,89
hsa-miR-1273c	URS000058878F_9606	0,713838435	1,640162133	0,40	NA
hsa-miR-3622a-3p	URS000048D51C_9606	-1,030868636	-2,043254109	0,40	NA
hsa-miR-4725-3p	URS000015A0D9_9606	0,759639771	1,693067827	0,40	NA
hsa-miR-135a-5p	URS00000DF6D0_9606	0,739122896	1,669160743	0,40	NA
hsa-miR-197-5p	URS000020E2DD_9606	-0,72424552	-1,652036452	0,40	NA
hsa-miR-320a-3p	URS00003CF1AD_9606	-0,347262149	-1,272144144	0,40	0,89
hsa-miR-1307-5p	URS00000EEF5F_9606	-0,612322002	-1,528717684	0,41	0,89
hsa-miR-548o-3p	URS0000080D0A_9606	0,837879695	1,787421267	0,41	NA
hsa-miR-500b-5p	URS00000CC005_9606	0,822759672	1,768786197	0,41	NA
hsa-miR-3688-3p	URS000032B492_9606	-0,67940239	-1,601476236	0,41	NA
hsa-miR-29a-5p	URS0000076995_9606	0,475074345	1,389989847	0,41	0,89
hsa-miR-335-5p	URS0000237AF9_9606	-0,397754827	-1,317456043	0,41	0,89
hsa-miR-3140-3p	URS000006B702_9606	-0,629429274	-1,546952903	0,41	NA
hsa-miR-1284	URS00004959F4_9606	-0,996104213	-1,994606578	0,41	NA
hsa-miR-136-3p	URS0000204177_9606	-0,987876928	-1,983264269	0,41	0,89
hsa-miR-4726-5p	URS0000026924_9606	0,838322085	1,787969448	0,42	NA
hsa-miR-3127-5p	URS0000170D8B_9606	-0,597191373	-1,512768653	0,42	NA

hsa-miR-194-5p	URS000029C2DC_9606	0,58229446	1,497228546	0,42	0,89
hsa-miR-7158-3p	URS00007E354A_9606	-0,568012081	-1,482479424	0,42	NA
hsa-miR-744-5p	URS00002ED61F_9606	0,424110351	1,341744851	0,42	0,89
hsa-miR-877-5p	URS00005240FD_9606	-0,49224617	-1,406633196	0,42	NA
hsa-miR-208b-3p	URS000034979B_9606	-1,487275862	-2,80359093	0,42	NA
hsa-miR-1343-3p	URS00000DA3DF_9606	-0,911765419	-1,881346285	0,42	NA
hsa-miR-652-3p	URS0000013DD8_9606	-0,587574136	-1,502717831	0,42	0,89
hsa-miR-134-5p	URS0000272A92_9606	-0,554648145	-1,468810365	0,42	0,89
hsa-miR-6859-5p	URS0000759D8E_9606	-0,910856022	-1,880160759	0,42	NA
hsa-miR-3150b-3p	URS0000383DF4_9606	-0,910724153	-1,879988912	0,42	NA
hsa-miR-6741-5p	URS000075A522_9606	-0,909941661	-1,878969516	0,43	NA
hsa-miR-3126-5p	URS0000022AAA_9606	-0,909941661	-1,878969516	0,43	NA
hsa-miR-3197	URS000060FA45_9606	-0,909941661	-1,878969516	0,43	NA
hsa-miR-548d-5p	URS00005F2D64_9606	-0,909941661	-1,878969516	0,43	NA
hsa-miR-6872-5p	URS000075B683_9606	-0,909809792	-1,878797778	0,43	NA
hsa-miR-2114-3p	URS000075BFBB_9606	-0,909809792	-1,878797778	0,43	NA
hsa-miR-27a-5p	URS00001B341F_9606	-0,559105584	-1,473355509	0,43	NA
hsa-miR-219b-5p	URS0000527B00_9606	-0,911770966	-1,881353518	0,43	NA
hsa-miR-4440	URS00003BFB30_9606	-0,911639097	-1,881181562	0,43	NA
hsa-miR-4662a-5p	URS00005140D0_9606	0,563906199	1,478266315	0,43	NA
hsa-miR-589-5p	URS00004214BB_9606	-0,520631973	-1,43458353	0,43	0,89
hsa-miR-127-5p	URS00000A33DB_9606	-0,784283246	-1,722236472	0,43	NA
hsa-miR-4429	URS0000004CA3_9606	0,792713794	1,732330014	0,43	NA
hsa-miR-3679-5p	URS00000D0BB4_9606	-0,615133879	-1,531700129	0,43	0,89
hsa-miR-6866-5p	URS000075CA93_9606	-0,56286988	-1,477204826	0,43	NA
hsa-miR-665	URS0000355E82_9606	-0,722673002	-1,650236735	0,44	NA
hsa-miR-4640-3p	URS000002EB45_9606	-0,911064957	-1,88043307	0,44	NA
hsa-miR-642a-3p	URS00000E7139_9606	-0,629908936	-1,547467313	0,44	0,89
hsa-miR-548aw	URS0000195F5B_9606	-0,545882146	-1,459912745	0,44	NA
hsa-miR-4454	URS00005D12AC_9606	0,506185401	1,420289869	0,45	0,89
hsa-miR-424-3p	URS00002BCF86_9606	0,753736328	1,686154025	0,45	NA
hsa-miR-1908-5p	URS00002373FD_9606	-0,900886316	-1,867212748	0,45	NA
hsa-miR-4798-5p	URS000057ED07_9606	-0,722277801	-1,649784745	0,45	NA
hsa-miR-202-5p	URS00003D8730_9606	-0,632687123	-1,550450131	0,45	0,89
hsa-miR-20a-3p	URS0000042E1F_9606	0,517820167	1,431790256	0,45	NA
hsa-miR-22-5p	URS0000142DC3_9606	-0,487776956	-1,402282438	0,45	0,89
hsa-miR-100-3p	URS00001A405B_9606	-0,452236174	-1,368159257	0,46	NA
hsa-miR-340-5p	URS0000007FBA_9606	-0,419387193	-1,33735937	0,46	0,89
hsa-miR-148b-5p	URS00005A7A84_9606	0,521583394	1,435529914	0,46	0,89

hsa-miR-6721-5p	URS000075E1DC_9606	0,965315034	1,95248983	0,46	NA
hsa-miR-4658	URS00002947E6_9606	0,964740649	1,951712633	0,46	NA
hsa-miR-4435	URS00002BBA1C_9606	0,964178032	1,950951661	0,46	NA
hsa-miR-4719	URS0000088093_9606	0,964178032	1,950951661	0,46	NA
hsa-miR-216a-3p	URS000075D097_9606	0,964178032	1,950951661	0,46	NA
hsa-miR-5581-3p	URS00001E2F7A_9606	0,964178032	1,950951661	0,46	NA
hsa-miR-135a-2-3p	URS000075AB6F_9606	0,964178031	1,95095166	0,46	NA
hsa-miR-6792-5p	URS000075D9C4_9606	0,964046224	1,950773425	0,46	NA
hsa-miR-548u	URS0000358C16_9606	0,964046224	1,950773425	0,46	NA
hsa-miR-6833-3p	URS000075AD96_9606	0,964046224	1,950773425	0,46	NA
hsa-miR-548av-3p	URS000075B010_9606	0,964046224	1,950773425	0,46	NA
hsa-miR-4799-5p	URS00001B09C2_9606	0,964046224	1,950773425	0,46	NA
hsa-miR-548m	URS000075ECFB_9606	0,964046224	1,950773425	0,46	NA
hsa-miR-3159	URS00002FB065_9606	0,964046224	1,950773425	0,46	NA
hsa-miR-1305	URS000040EC3B_9606	0,964046224	1,950773425	0,46	NA
hsa-miR-3657	URS000075C59B_9606	0,963914429	1,950595224	0,46	NA
hsa-miR-1298-5p	URS00003DF3AE_9606	0,963914429	1,950595224	0,46	NA
hsa-miR-6881-5p	URS000075DCF1_9606	0,963914429	1,950595224	0,46	NA
hsa-miR-4758-3p	URS0000031E74_9606	0,963914429	1,950595224	0,46	NA
hsa-miR-6840-3p	URS000075C594_9606	0,963914429	1,950595224	0,46	NA
hsa-miR-616-5p	URS00005D986D_9606	0,963914429	1,950595224	0,46	NA
hsa-miR-4326	URS0000555DA6_9606	0,963914429	1,950595224	0,46	NA
hsa-miR-1277-5p	URS0000427B68_9606	0,963914429	1,950595224	0,46	NA
hsa-miR-6752-3p	URS000075BA76_9606	0,963914429	1,950595224	0,46	NA
hsa-miR-1226-3p	URS00002971AB_9606	0,773012541	1,708834337	0,46	NA
hsa-miR-552-5p	URS000075AF8C_9606	0,615637826	1,53223526	0,47	NA
hsa-miR-374b-3p	URS00001EFEC1_9606	-0,564848931	-1,479232606	0,47	NA
hsa-miR-221-3p	URS0000170CF4_9606	-0,291755446	-1,224128871	0,47	0,89
hsa-miR-5010-5p	URS00000ECE9C_9606	-0,576970212	-1,491713227	0,48	NA
hsa-miR-503-5p	URS00000F6E49_9606	-0,553530588	-1,467673019	0,48	0,89
hsa-miR-511-5p	URS000028DE3F_9606	-0,911639092	-1,881181555	0,48	NA
hsa-miR-150-3p	URS00005EAAAD_9606	0,525631929	1,439564	0,48	NA
hsa-miR-29c-5p	URS0000497496_9606	0,507081542	1,421172366	0,48	0,89
hsa-miR-26b-3p	URS000021C6A8_9606	0,494130381	1,408471509	0,48	NA
hsa-miR-431-5p	URS000043908D_9606	0,582621585	1,497568075	0,48	NA
hsa-miR-22-3p	URS0000096022_9606	-0,353883102	-1,277995806	0,48	0,89
hsa-miR-4714-3p	URS0000439509_9606	-0,680703725	-1,602921445	0,49	NA
hsa-miR-7976	URS000075B11C_9606	0,850475973	1,803095704	0,49	NA
hsa-miR-6873-3p	URS0000759B8E_9606	-0,766373941	-1,700989152	0,49	NA

hsa-miR-148a-5p	URS00003E16E5_9606	-0,401455264	-1,320839586	0,49	0,89
hsa-miR-6502-5p	URS000075A771_9606	0,796440807	1,736811046	0,49	NA
hsa-miR-642a-5p	URS00000F2C33_9606	-0,662521978	-1,582847185	0,49	NA
hsa-miR-378b	URS0000328C0D_9606	0,791706568	1,731121	0,49	NA
hsa-miR-3200-3p	URS0000381B86_9606	0,591886454	1,507216281	0,49	0,89
hsa-miR-196a-5p	URS00000DA6A7_9606	0,684810135	1,607490415	0,49	NA
hsa-miR-1307-3p	URS00004B1671_9606	0,374061206	1,295995946	0,49	0,89
hsa-miR-574-5p	URS000057466C_9606	0,478230685	1,393034207	0,49	0,89
hsa-miR-3190-3p	URS00005ACA7C_9606	-0,674023867	-1,595516874	0,49	NA
hsa-miR-590-3p	URS0000272039_9606	-0,390343378	-1,31070533	0,50	0,89
hsa-miR-127-3p	URS00001E3DAA_9606	-0,472151339	-1,387176477	0,50	0,89
hsa-miR-6734-5p	URS000075E8C3_9606	-0,630970958	-1,548606882	0,50	0,89
hsa-miR-624-5p	URS000031BC7D_9606	-0,541739938	-1,455727114	0,50	NA
hsa-miR-664a-3p	URS000029AE45_9606	0,454107292	1,369934857	0,50	0,89
hsa-miR-1277-3p	URS000044878A_9606	0,631370153	1,549035442	0,50	NA
hsa-miR-488-3p	URS00001BCAC5_9606	-0,351947536	-1,276282355	0,51	0,89
hsa-miR-6847-5p	URS000075DD4B_9606	0,725326761	1,65327505	0,51	NA
hsa-miR-329-3p	URS00004C46CF_9606	-0,795072493	-1,735164562	0,51	NA
hsa-miR-345-3p	URS0000384659_9606	0,724884437	1,652768241	0,51	NA
hsa-miR-19b-1-5p	URS00001B9622_9606	0,724884437	1,652768241	0,51	NA
hsa-miR-4781-3p	URS0000128A0A_9606	0,724884437	1,652768241	0,51	NA
hsa-miR-548ab	URS0000496C56_9606	0,724752704	1,652617333	0,51	NA
hsa-miR-550a-5p	URS00003FFA6C_9606	0,724752704	1,652617333	0,51	NA
hsa-miR-1911-5p	URS00007455B8_9606	0,724752704	1,652617333	0,51	NA
hsa-miR-1266-5p	URS00005FDCD1_9606	0,724752704	1,652617333	0,51	NA
hsa-miR-105-5p	URS0000495F5B_9606	0,724400056	1,652213421	0,51	NA
hsa-miR-320b	URS000058BF17_9606	-0,321599665	-1,249715469	0,51	0,89
hsa-miR-3133	URS00002CF3F0_9606	0,723579645	1,651274131	0,51	NA
hsa-miR-4669	URS00001062A2_9606	-0,890961889	-1,854412106	0,51	NA
hsa-miR-34a-5p	URS000030BD69_9606	-0,353096512	-1,277299204	0,51	0,89
hsa-miR-99b-3p	URS00001C308D_9606	0,304013001	1,234573732	0,51	0,89
hsa-miR-6833-5p	URS000075D442_9606	-0,833318797	-1,781779483	0,51	NA
hsa-miR-449a	URS00001F5B39_9606	-0,674023867	-1,595516874	0,51	NA
hsa-miR-154-5p	URS000008931A_9606	-0,712713376	-1,638883581	0,51	0,89
hsa-miR-1225-5p	URS000075D0F5_9606	-0,674806017	-1,596382111	0,52	NA
hsa-miR-342-5p	URS00005A8080_9606	-0,538421504	-1,452382552	0,52	NA
hsa-miR-505-3p	URS00004A5A07_9606	0,412310772	1,330815682	0,52	0,89
hsa-miR-381-3p	URS00001FFA8C_9606	-0,488756279	-1,403234652	0,52	0,89
hsa-miR-140-5p	URS000026261D_9606	0,39919333	1,318770326	0,52	0,89

hsa-miR-122-5p	URS00003380CC_9606	0,447661017	1,363827345	0,53	0,89
hsa-miR-144-3p	URS000037C5A8_9606	0,506481232	1,420581135	0,53	0,89
hsa-miR-10527-5p	URS0000D5310C_9606	-0,393789365	-1,313839791	0,53	0,89
hsa-miR-224-5p	URS0000D55DFB_9606	0,408449344	1,327258466	0,53	0,89
hsa-miR-140-3p	URS00000821E0_9606	-0,382509535	-1,303607479	0,53	0,89
hsa-miR-500b-3p	URS000075B475_9606	-0,598007144	-1,513624289	0,53	NA
hsa-miR-378g	URS000018E6CD_9606	-0,372226991	-1,294349289	0,53	0,89
hsa-miR-3605-5p	URS000018C677_9606	-0,51145272	-1,425484863	0,53	NA
hsa-miR-1587	URS00000ECE90_9606	-0,934230748	-1,910871477	0,53	NA
hsa-miR-10398-5p	URS0000D532D3_9606	-0,934230748	-1,910871477	0,53	NA
hsa-miR-4653-5p	URS00002D0CF3_9606	-0,934230748	-1,910871477	0,53	NA
hsa-miR-4667-3p	URS00001CE046_9606	-0,934230748	-1,910871477	0,53	NA
hsa-miR-449c-5p	URS00002198F3_9606	-0,934230748	-1,910871477	0,53	NA
hsa-miR-3121-3p	URS000052EA98_9606	-0,934230748	-1,910871477	0,53	NA
hsa-miR-124-5p	URS00003AA409_9606	-0,934230748	-1,910871477	0,53	NA
hsa-miR-4795-5p	URS000045C62E_9606	-0,934230748	-1,910871477	0,53	NA
hsa-miR-219a-2-3p	URS00004ACAAA_9606	-0,934230748	-1,910871477	0,53	NA
hsa-miR-5090	URS000055929F_9606	-0,934230748	-1,910871477	0,53	NA
hsa-miR-890	URS0000759F06_9606	-0,669882121	-1,59094297	0,53	NA
hsa-miR-3667-3p	URS000075E2C3_9606	-0,934203287	-1,910835105	0,53	NA
hsa-miR-6730-5p	URS000075EC2A_9606	-0,934203287	-1,910835105	0,53	NA
hsa-miR-4324	URS000030F801_9606	-0,934203287	-1,910835105	0,53	NA
hsa-miR-6837-3p	URS000075E6F0_9606	-0,934203287	-1,910835105	0,53	NA
hsa-miR-3183	URS000047944F_9606	-0,934203287	-1,910835105	0,53	NA
hsa-miR-6809-5p	URS0000759948_9606	-0,934203287	-1,910835105	0,53	NA
hsa-miR-6751-5p	URS000075A393_9606	-0,934203287	-1,910835105	0,53	NA
hsa-miR-6737-5p	URS000075C8FF_9606	-0,934203287	-1,910835105	0,53	NA
hsa-miR-6894-3p	URS000075D303_9606	-0,934203287	-1,910835105	0,53	NA
hsa-miR-6867-5p	URS0000759AB5_9606	-0,934077118	-1,910668003	0,53	NA
hsa-miR-10394-3p	URS0000D5480A_9606	-0,934077118	-1,910668003	0,53	NA
hsa-miR-4635	URS000035709C_9606	-0,934077118	-1,910668003	0,53	NA
hsa-miR-4680-3p	URS00005CFD69_9606	-0,934077118	-1,910668003	0,53	NA
hsa-miR-6728-3p	URS000075B93D_9606	-0,934077118	-1,910668003	0,53	NA
hsa-miR-4803	URS0000461D80_9606	-0,934077118	-1,910668003	0,53	NA
hsa-miR-7850-5p	URS0000759D67_9606	-0,934077118	-1,910668003	0,53	NA
hsa-miR-328-5p	URS000075EEF2_9606	-0,934077118	-1,910668003	0,53	NA
hsa-miR-6507-5p	URS000075EF6B_9606	-0,934077118	-1,910668003	0,53	NA
hsa-miR-6769b-3p	URS000075CAD0_9606	-0,934077118	-1,910668003	0,53	NA
hsa-miR-5089-3p	URS000075D5F3_9606	-0,934077118	-1,910668003	0,53	NA

hsa-miR-4650-3p	URS00003151AF_9606	-0,934077118	-1,910668003	0,53	NA
hsa-miR-3648	URS0000454FAB_9606	-0,934077118	-1,910668003	0,53	NA
hsa-miR-4708-5p	URS0000122F23_9606	-0,934077118	-1,910668003	0,53	NA
hsa-miR-6758-3p	URS000075B07E_9606	-0,934077118	-1,910668003	0,53	NA
hsa-miR-6783-3p	URS000075A2EF_9606	-0,934077118	-1,910668003	0,53	NA
hsa-miR-3909	URS000022B729_9606	-0,934077118	-1,910668003	0,53	NA
hsa-miR-584-3p	URS000006BE7D_9606	-0,934077118	-1,910668003	0,53	NA
hsa-miR-1827	URS000056215C_9606	-0,934077118	-1,910668003	0,53	NA
hsa-miR-5583-3p	URS000075CAB4_9606	-0,934077118	-1,910668003	0,53	NA
hsa-miR-6831-5p	URS000075E08F_9606	-0,933161949	-1,909456361	0,53	NA
hsa-miR-4529-3p	URS000075E7E0_9606	-0,933161949	-1,909456361	0,53	NA
hsa-miR-5001-5p	URS00001F373E_9606	-0,933161949	-1,909456361	0,53	NA
hsa-miR-7703	URS00003F7D60_9606	-0,933161949	-1,909456361	0,53	NA
hsa-miR-106a-3p	URS00005F5B9E_9606	-0,933161949	-1,909456361	0,53	NA
hsa-miR-4446-3p	URS000000EF0B_9606	-0,933161949	-1,909456361	0,53	NA
hsa-miR-4645-5p	URS000010BD8D_9606	-0,933161949	-1,909456361	0,53	NA
hsa-miR-2115-5p	URS000075D549_9606	-0,933161949	-1,909456361	0,53	NA
hsa-miR-767-5p	URS000045147B_9606	-0,933161949	-1,909456361	0,53	NA
hsa-miR-3940-3p	URS000008B558_9606	-0,933161949	-1,909456361	0,53	NA
hsa-miR-6767-5p	URS000075A5E4_9606	-0,933161949	-1,909456361	0,53	NA
hsa-miR-4470	URS00002AAD6B_9606	-0,933161949	-1,909456361	0,53	NA
hsa-miR-656-3p	URS0000202460_9606	-0,933161949	-1,909456361	0,53	NA
hsa-miR-6851-3p	URS000075C5AE_9606	-0,933161949	-1,909456361	0,53	NA
hsa-miR-6737-3p	URS000075E2AA_9606	-0,933161949	-1,909456361	0,53	NA
hsa-miR-6799-5p	URS000075ACE6_9606	-0,933161949	-1,909456361	0,53	NA
hsa-miR-10401-5p	URS0000D51CA4_9606	-0,933161949	-1,909456361	0,53	NA
hsa-miR-3196	URS000033B548_9606	-0,933161949	-1,909456361	0,53	NA
hsa-miR-4661-5p	URS000011C106_9606	-0,933161949	-1,909456361	0,53	NA
hsa-miR-3606-3p	URS000075D027_9606	-0,933161949	-1,909456361	0,53	NA
hsa-miR-3173-3p	URS000017CCCB_9606	-0,933161949	-1,909456361	0,53	NA
hsa-miR-216b-5p	URS000046CDA6_9606	-0,933161949	-1,909456361	0,53	NA
hsa-miR-4670-5p	URS000015D252_9606	-0,933161949	-1,909456361	0,53	NA
hsa-miR-3914	URS00005547BA_9606	-0,933161949	-1,909456361	0,53	NA
hsa-miR-7845-5p	URS000075ED16_9606	-0,933161949	-1,909456361	0,53	NA
hsa-miR-410-3p	URS000047E765_9606	-0,933161949	-1,909456361	0,53	NA
hsa-miR-487a-3p	URS000016FD1B_9606	-0,933161949	-1,909456361	0,53	NA
hsa-miR-4485-5p	URS00007649C8_9606	-0,933161949	-1,909456361	0,53	NA
hsa-miR-3140-5p	URS0000366959_9606	-0,933161949	-1,909456361	0,53	NA
hsa-miR-6742-3p	URS000075BC3E_9606	-0,933161949	-1,909456361	0,53	NA

hsa-miR-1251-5p	URS00002B4E74_9606	-0,933161949	-1,909456361	0,53	NA
hsa-miR-6876-3p	URS000075C81D_9606	-0,933161949	-1,909456361	0,53	NA
hsa-miR-6851-5p	URS000075ACF5_9606	-0,933161949	-1,909456361	0,53	NA
hsa-miR-4430	URS0000221168_9606	-0,933161949	-1,909456361	0,53	NA
hsa-miR-466	URS00002F5FEE_9606	-0,933161949	-1,909456361	0,53	NA
hsa-miR-4460	URS000075E77E_9606	-0,933161949	-1,909456361	0,53	NA
hsa-miR-218-1-3p	URS00001C4762_9606	-0,933161949	-1,909456361	0,53	NA
hsa-miR-544a	URS00001934CD_9606	-0,933161949	-1,909456361	0,53	NA
hsa-miR-548am-3p	URS0000450D02_9606	-0,933161949	-1,909456361	0,53	NA
hsa-miR-548j-3p	URS0000759BFD_9606	-0,933161949	-1,909456361	0,53	NA
hsa-miR-561-3p	URS000075D1DD_9606	-0,933161949	-1,909456361	0,53	NA
hsa-miR-624-3p	URS00004E6C7F_9606	-0,933161949	-1,909456361	0,53	NA
hsa-miR-3120-3p	URS0000136174_9606	-0,933161949	-1,909456361	0,53	NA
hsa-miR-4773	URS0000410073_9606	-0,933161949	-1,909456361	0,53	NA
hsa-miR-5088-5p	URS00002F0130_9606	-0,933161949	-1,909456361	0,53	NA
hsa-miR-3617-3p	URS000075EDB5_9606	-0,933161949	-1,909456361	0,53	NA
hsa-miR-6890-5p	URS000075AA8B_9606	-0,933161949	-1,909456361	0,53	NA
hsa-miR-4762-5p	URS00002CAB63_9606	-0,933161949	-1,909456361	0,53	NA
hsa-miR-3155a	URS00002D349B_9606	-0,933161949	-1,909456361	0,53	NA
hsa-miR-4749-3p	URS0000077DDE_9606	-0,933161949	-1,909456361	0,53	NA
hsa-miR-363-5p	URS00000B989F_9606	-0,933161949	-1,909456361	0,53	NA
hsa-miR-7151-3p	URS000075CA04_9606	-0,933161949	-1,909456361	0,53	NA
hsa-miR-6802-5p	URS000075CF39_9606	-0,933161949	-1,909456361	0,53	NA
hsa-miR-6769a-3p	URS000075B844_9606	-0,933161949	-1,909456361	0,53	NA
hsa-miR-4761-3p	URS000046C832_9606	-0,933161949	-1,909456361	0,53	NA
hsa-miR-556-5p	URS000033CC69_9606	-0,933161949	-1,909456361	0,53	NA
hsa-miR-4271	URS00002476CC_9606	-0,933161949	-1,909456361	0,53	NA
hsa-miR-376c-5p	URS00001B9F58_9606	-0,933161949	-1,909456361	0,53	NA
hsa-miR-1229-5p	URS00008E395F_9606	-0,933161949	-1,909456361	0,53	NA
hsa-miR-6848-3p	URS000075A993_9606	-0,933161949	-1,909456361	0,53	NA
hsa-miR-653-5p	URS000061E50F_9606	-0,933161949	-1,909456361	0,53	NA
hsa-miR-1912-3p	URS00004A9320_9606	-0,933161949	-1,909456361	0,53	NA
hsa-miR-1197	URS00003E5E03_9606	-0,933161949	-1,909456361	0,53	NA
hsa-miR-1245b-5p	URS00003B1644_9606	-0,933161949	-1,909456361	0,53	NA
hsa-miR-4688	URS000032D69B_9606	-0,933161949	-1,909456361	0,53	NA
hsa-miR-135a-3p	URS0000169868_9606	-0,933161949	-1,909456361	0,53	NA
hsa-miR-3675-5p	URS000075A0D7_9606	-0,933161949	-1,909456361	0,53	NA
hsa-miR-4501	URS000075AD69_9606	-0,933161949	-1,909456361	0,53	NA
hsa-miR-4457	URS000075AB35_9606	-0,933161949	-1,909456361	0,53	NA

hsa-miR-5690	URS000075B186_9606	-0,933161949	-1,909456361	0,53	NA
hsa-miR-5196-3p	URS000075E072_9606	-0,933161949	-1,909456361	0,53	NA
hsa-miR-4716-5p	URS00002DA7E9_9606	-0,933161949	-1,909456361	0,53	NA
hsa-miR-4744	URS00004B9C33_9606	-0,933161949	-1,909456361	0,53	NA
hsa-miR-3664-3p	URS000043C1C3_9606	-0,933161949	-1,909456361	0,53	NA
hsa-miR-6824-3p	URS000075A22A_9606	-0,933161949	-1,909456361	0,53	NA
hsa-miR-7157-3p	URS000075BDE7_9606	-0,933161949	-1,909456361	0,53	NA
hsa-miR-3928-5p	URS0000759D35_9606	-0,933161949	-1,909456361	0,53	NA
hsa-miR-5585-5p	URS000075B824_9606	-0,933161949	-1,909456361	0,53	NA
hsa-miR-2467-5p	URS00000C4DB5_9606	-0,933161949	-1,909456361	0,53	NA
hsa-miR-24-2-5p	URS00001DEE11_9606	-0,933161949	-1,909456361	0,53	NA
hsa-miR-5580-5p	URS000075B5F4_9606	-0,933161949	-1,909456361	0,53	NA
hsa-miR-4687-3p	URS000047456A_9606	-0,933161949	-1,909456361	0,53	NA
hsa-miR-3180-3p	URS00002C4233_9606	-0,933161949	-1,909456361	0,53	NA
hsa-miR-4664-5p	URS00005A06DD_9606	-0,933161949	-1,909456361	0,53	NA
hsa-miR-6745	URS000075AE63_9606	-0,933161949	-1,909456361	0,53	NA
hsa-miR-4672	URS000028BB78_9606	-0,933161949	-1,909456361	0,53	NA
hsa-miR-4788	URS0000249A2E_9606	-0,933161949	-1,909456361	0,53	NA
hsa-miR-516a-5p	URS000056DB1D_9606	-0,933161949	-1,909456361	0,53	NA
hsa-miR-448	URS0000347D0F_9606	-0,933161949	-1,909456361	0,53	NA
hsa-miR-6819-5p	URS000075DD69_9606	-0,933161949	-1,909456361	0,53	NA
hsa-miR-592	URS00004F507C_9606	-0,933161949	-1,909456361	0,53	NA
hsa-miR-4503	URS000075A348_9606	-0,933161949	-1,909456361	0,53	NA
hsa-miR-493-5p	URS0000077AED_9606	-0,436648026	-1,353456037	0,53	NA
hsa-miR-1261	URS00003975B5_9606	-0,437105617	-1,353885391	0,53	NA
hsa-miR-1304-3p	URS000050FB05_9606	-0,420162232	-1,338078014	0,53	0,886065224
hsa-miR-376b-5p	URS000047736C_9606	0,723121925	1,65075032	0,53	NA
hsa-miR-432-5p	URS00001C406A_9606	-0,653103452	-1,572547343	0,54	0,886065224
hsa-miR-5008-3p	URS000075D2C7_9606	-0,934230747	-1,910871477	0,54	NA
hsa-miR-23a-3p	URS00005540D2_9606	0,322948127	1,250884103	0,54	0,886065224
hsa-miR-183-3p	URS0000345DEB_9606	0,592377937	1,507729832	0,54	NA
hsa-miR-433-3p	URS000024DFCE_9606	0,592377937	1,507729832	0,54	NA
hsa-miR-3173-5p	URS00004216E6_9606	0,59133697	1,506642331	0,54	NA
hsa-miR-769-5p	URS00004E008F_9606	0,32348555	1,251350161	0,54	0,886065224
hsa-miR-548ac	URS00002CE0C6_9606	0,723579645	1,651274131	0,54	NA
hsa-miR-628-3p	URS000061BE3B_9606	0,370495056	1,292796373	0,54	0,886065224
hsa-miR-501-3p	URS00000EEE35_9606	-0,336920135	-1,26305734	0,54	0,886065224
hsa-miR-147b-3p	URS00007E4A5A_9606	-0,40263818	-1,321923032	0,54	NA
hsa-miR-338-3p	URS00000254A6_9606	-0,398784836	-1,318396974	0,54	0,886065224

hsa-miR-892b	URS000075A42A_9606	-0,557923906	-1,472149212	0,54	NA
hsa-miR-361-5p	URS00000CF1D2_9606	0,317300658	1,245997055	0,55	0,886065224
hsa-miR-548n	URS00000AD02D_9606	-0,640964711	-1,559371542	0,55	NA
hsa-miR-548c-5p	URS00002C02E4_9606	-0,357635484	-1,28132414	0,55	NA
hsa-miR-30a-3p	URS0000065D58_9606	0,315617871	1,244544547	0,55	0,886065224
hsa-miR-548aq-5p	URS000075D1B2_9606	0,723683905	1,651393469	0,55	NA
hsa-miR-548t-3p	URS000012930C_9606	-0,528067256	-1,441996093	0,55	NA
hsa-miR-6827-5p	URS000075D5D0_9606	-0,527285434	-1,441214861	0,55	NA
hsa-miR-363-3p	URS000038B599_9606	-0,401472234	-1,320855123	0,55	0,886065224
hsa-miR-892a	URS000075AA5C_9606	-0,388979072	-1,309466428	0,55	0,886065224
hsa-miR-30d-3p	URS00004B2A47_9606	-0,352037069	-1,276361563	0,56	0,886065224
hsa-miR-760	URS0000512C88_9606	-0,34337985	-1,268725401	0,56	0,886065224
hsa-miR-454-5p	URS000031602A_9606	0,374449216	1,296344549	0,56	0,886065224
hsa-miR-1275	URS000009EA8F_9606	-0,402077977	-1,321409825	0,56	NA
hsa-miR-422a	URS00003CC245_9606	-0,373401161	-1,295403153	0,56	NA
hsa-miR-653-3p	URS000075C1A7_9606	0,508170695	1,422245676	0,56	NA
hsa-let-7a-3p	URS000004F5D8_9606	0,312293136	1,241679758	0,56	0,886065224
hsa-miR-193b-3p	URS00000AA464_9606	-0,37380597	-1,295766685	0,56	0,886065224
hsa-miR-3115	URS00000BB229_9606	-0,528193564	-1,442122346	0,56	NA
hsa-miR-100-5p	URS000040D674_9606	0,271384262	1,206965353	0,56	0,886065224
hsa-miR-3117-3p	URS0000507F3E_9606	-0,527417155	-1,441346454	0,57	NA
hsa-miR-139-5p	URS000025D232_9606	0,432258258	1,34934406	0,57	0,886065224
hsa-miR-548ay-5p	URS000075EF99_9606	-0,442447374	-1,358907611	0,57	NA
hsa-miR-4732-5p	URS0000240326_9606	-0,416505551	-1,334690792	0,57	0,886065224
hsa-miR-4766-3p	URS00000B6FA1_9606	-0,675984156	-1,597686286	0,57	NA
hsa-miR-4306	URS00006097A6_9606	-0,431130393	-1,348289587	0,57	0,886065224
hsa-let-7i-3p	URS0000237CBD_9606	-0,445737735	-1,362010415	0,57	0,886065224
hsa-miR-200b-3p	URS000014D9C1_9606	0,275616171	1,210510985	0,57	0,886065224
hsa-miR-3615	URS000011166D_9606	-0,499512644	-1,413735908	0,57	0,886065224
hsa-miR-32-3p	URS00002FC5B0_9606	0,687341126	1,610312989	0,57	NA
hsa-miR-641	URS000039D790_9606	0,568823491	1,483313444	0,57	NA
hsa-miR-6826-5p	URS000075BCB6_9606	0,590811149	1,506093303	0,57	NA
hsa-miR-766-3p	URS00001012BC_9606	0,448878434	1,364978696	0,58	NA
hsa-let-7a-2-3p	URS00000157F5_9606	0,508039109	1,422115961	0,58	NA
hsa-miR-671-5p	URS00002FB368_9606	-0,281771991	-1,215687137	0,58	0,886065224
hsa-miR-483-5p	URS000003575B_9606	0,407624171	1,326499536	0,58	0,886065224
hsa-miR-25-3p	URS00004F9744_9606	-0,247292928	-1,18697778	0,58	0,886065224
hsa-miR-421	URS00004505E4_9606	-0,304108657	-1,234655592	0,58	0,886065224
hsa-miR-3158-3p	URS000040D577_9606	0,50699851	1,421090576	0,58	NA

hsa-miR-629-5p	URS00002F3336_9606	-0,398920419	-1,318520881	0,58	0,886065224
hsa-miR-20a-5p	URS0000574A2C_9606	0,344829788	1,270001136	0,58	0,886065224
hsa-miR-320e	URS00003B6B84_9606	-0,463531457	-1,37891302	0,58	NA
hsa-miR-504-5p	URS00005CC20C_9606	0,401998034	1,321336605	0,58	0,887594893
hsa-miR-548bc	URS0000D531E8_9606	-0,715877685	-1,642482142	0,59	NA
hsa-miR-195-5p	URS00005B3525_9606	-0,301008531	-1,232005359	0,59	0,887594893
hsa-miR-1306-5p	URS0000500449_9606	-0,713195628	-1,639431505	0,59	NA
hsa-miR-2278	URS000059EB31_9606	0,559285291	1,473539047	0,59	NA
hsa-miR-1260a	URS00000D0874_9606	0,295599543	1,227394942	0,59	0,887594893
hsa-miR-6503-5p	URS000075B0F8_9606	-0,641704586	-1,560171459	0,59	NA
hsa-miR-374a-3p	URS0000285DCB_9606	0,438420099	1,355119518	0,59	0,887594893
hsa-miR-3129-3p	URS000033351F_9606	0,591205322	1,506504855	0,59	NA
hsa-miR-200a-3p	URS000008DA94_9606	-0,297098576	-1,22867093	0,59	0,887594893
hsa-miR-4645-3p	URS0000212118_9606	-0,335166875	-1,26152332	0,60	NA
hsa-miR-132-3p	URS00006054DA_9606	0,260124412	1,197581975	0,60	0,887594893
hsa-miR-570-3p	URS0000250A40_9606	-0,441826316	-1,358322748	0,60	NA
hsa-miR-219a-5p	URS0000565C8D_9606	0,343693378	1,269001151	0,60	NA
hsa-miR-210-5p	URS000075D16F_9606	-0,66118891	-1,58138529	0,60	NA
hsa-miR-10526-3p	URS0000D52683_9606	-0,374838505	-1,296694395	0,60	NA
hsa-miR-27a-3p	URS00003B95DA_9606	-0,305585229	-1,235919886	0,60	0,887594893
hsa-miR-765	URS0000235862_9606	-0,442583511	-1,359035847	0,61	NA
hsa-miR-196b-5p	URS0000611746_9606	-0,436397832	-1,353221338	0,61	0,887594893
hsa-miR-1537-3p	URS000010F787_9606	-0,528221067	-1,442149838	0,61	NA
hsa-miR-125a-3p	URS00001F0C23_9606	-0,273835861	-1,209018115	0,61	0,887594893
hsa-miR-1976	URS000075BD0D_9606	0,429569203	1,346831346	0,61	NA
hsa-miR-450b-5p	URS0000422A99_9606	-0,37273566	-1,294805734	0,61	0,887594893
hsa-miR-3064-5p	URS000021ECD0_9606	0,70702013	1,632428869	0,61	NA
hsa-miR-10a-3p	URS00002F4762_9606	-0,293760532	-1,225831372	0,61	0,887594893
hsa-miR-874-3p	URS00005609ED_9606	-0,36336567	-1,286423503	0,61	0,887594893
hsa-miR-628-5p	URS00004B83FE_9606	-0,341761295	-1,267302819	0,61	0,887594893
hsa-miR-346	URS0000134ADC_9606	-0,593308076	-1,508702214	0,61	NA
hsa-miR-130b-5p	URS000032A4F7_9606	-0,740905139	-1,671224027	0,62	NA
hsa-miR-1287-5p	URS00004C5774_9606	-0,288970198	-1,221767863	0,62	0,887594893
hsa-miR-107	URS00005743AE_9606	-0,253604027	-1,192181612	0,62	0,887594893
hsa-miR-3154	URS0000519450_9606	-0,525884933	-1,439816476	0,62	NA
hsa-miR-6503-3p	URS000075BDA3_9606	-0,336538039	-1,262722864	0,62	NA
hsa-miR-218-5p	URS000020D84A_9606	-0,252159637	-1,190988628	0,62	0,891504172
hsa-miR-5189-3p	URS000075B757_9606	0,590811149	1,506093304	0,62	NA
hsa-miR-3126-3p	URS0000052E4D_9606	-0,582216565	-1,497147709	0,62	NA

hsa-miR-3652	URS000075DC58_9606	-0,581301101	-1,496197993	0,62	NA
hsa-miR-7705	URS000060B2B9_9606	-0,581301101	-1,496197993	0,62	NA
hsa-miR-4467	URS000075A41A_9606	-0,581147426	-1,496038628	0,62	NA
hsa-miR-939-5p	URS00005A31EB_9606	-0,581147426	-1,496038628	0,62	NA
hsa-miR-6733-3p	URS000075DF73_9606	-0,581015557	-1,49590189	0,62	NA
hsa-miR-6509-5p	URS000075AABA_9606	-0,581015557	-1,49590189	0,62	NA
hsa-miR-1250-5p	URS0000277201_9606	-0,581015557	-1,49590189	0,62	NA
hsa-miR-34b-3p	URS000027352D_9606	-0,582084697	-1,49701087	0,63	NA
hsa-miR-6853-3p	URS000075D50A_9606	-0,580232662	-1,495090341	0,63	NA
hsa-miR-3612	URS000075B853_9606	-0,580232662	-1,495090341	0,63	NA
hsa-miR-1258	URS000075C57C_9606	-0,580232662	-1,495090341	0,63	NA
hsa-miR-766-5p	URS00002B8C45_9606	-0,580232662	-1,495090341	0,63	NA
hsa-miR-4524a-5p	URS000075C58E_9606	-0,580232662	-1,495090341	0,63	NA
hsa-miR-6813-5p	URS000075AEBC_9606	-0,580232662	-1,495090341	0,63	NA
hsa-miR-4772-3p	URS00005901AD_9606	-0,580232662	-1,495090341	0,63	NA
hsa-miR-652-5p	URS000030C9DE_9606	-0,580100793	-1,494953689	0,63	NA
hsa-miR-6888-3p	URS000075B03C_9606	-0,580100793	-1,494953689	0,63	NA
hsa-miR-548ag	URS0000601CA2_9606	-0,580100793	-1,494953689	0,63	NA
hsa-miR-548ao-3p	URS000075CCE4_9606	-0,580100793	-1,494953689	0,63	NA
hsa-miR-101-2-5p	URS000075EB3E_9606	-0,580100793	-1,494953689	0,63	NA
hsa-miR-940	URS000041FA2C_9606	-0,580100793	-1,494953689	0,63	NA
hsa-miR-3613-5p	URS0000045DBD_9606	-0,254723967	-1,193107442	0,63	0,893002336
hsa-miR-455-3p	URS000022A78C_9606	0,37957809	1,300961339	0,63	NA
hsa-miR-3144-3p	URS0000393AD8_9606	-0,579538183	-1,494370813	0,63	NA
hsa-miR-3150a-3p	URS000014F13C_9606	0,506657458	1,420754671	0,63	NA
hsa-miR-491-3p	URS0000202B65_9606	-0,581522086	-1,496427192	0,63	NA
hsa-miR-708-5p	URS000019D79B_9606	0,264580014	1,201286289	0,64	0,904070957
hsa-miR-548at-5p	URS000075D269_9606	-0,354106998	-1,278194158	0,64	NA
hsa-miR-5585-3p	URS00003E6EFA_9606	-0,46208911	-1,377535129	0,64	NA
hsa-miR-132-5p	URS0000028BB8_9606	0,285863037	1,219139349	0,64	NA
hsa-miR-3928-3p	URS000057FDB8_9606	0,390625683	1,310961832	0,64	NA
hsa-miR-106b-3p	URS0000384021_9606	-0,301479163	-1,232407326	0,64	0,906628964
hsa-miR-17-5p	URS00002075FA_9606	0,247816323	1,187408482	0,64	0,906628964
hsa-miR-2277-5p	URS00000D6C3F_9606	0,407627246	1,326502363	0,65	NA
hsa-miR-23c	URS00002DD349_9606	-0,580100793	-1,494953689	0,65	NA
hsa-miR-379-5p	URS000060A1F4_9606	-0,40883168	-1,327610256	0,65	0,90920424
hsa-miR-4286	URS0000353492_9606	0,351555871	1,275935916	0,65	NA
hsa-miR-370-3p	URS00004900F1_9606	-0,509752404	-1,42380582	0,66	NA
hsa-miR-6511b-3p	URS0000759CCE_9606	-0,526028329	-1,439959594	0,66	NA

hsa-miR-9985	URS0000D4FAF7_9606	-0,264775611	-1,201449168	0,66	NA
hsa-miR-7706	URS00005B08C1_9606	0,293776073	1,225844577	0,66	NA
hsa-miR-369-5p	URS00002A71AD_9606	-0,359839709	-1,28328331	0,67	0,916547009
hsa-miR-200a-5p	URS000023B77E_9606	-0,304244582	-1,234771922	0,67	0,916547009
hsa-miR-598-3p	URS000028FD1A_9606	0,247876247	1,187457803	0,67	0,916547009
hsa-miR-3182	URS00002FAA8C_9606	0,25724716	1,195195947	0,67	0,916547009
hsa-miR-505-5p	URS000017EA6A_9606	-0,342053575	-1,267559593	0,67	0,916547009
hsa-miR-181b-5p	URS0000605E00_9606	0,193771193	1,143749564	0,67	0,916547009
hsa-miR-548h-5p	URS00003E16D3_9606	-0,298945053	-1,230244488	0,67	NA
hsa-miR-4653-3p	URS000035646E_9606	-0,608270672	-1,52443081	0,67	NA
hsa-miR-141-3p	URS000003E1A9_9606	0,315478562	1,244424378	0,67	0,918863994
hsa-miR-3074-5p	URS0000166778_9606	0,405158686	1,324234559	0,67	NA
hsa-let-7d-3p	URS000028D142_9606	-0,308333329	-1,238276357	0,68	0,920034518
hsa-miR-331-3p	URS00003DDE27_9606	-0,36096608	-1,284285613	0,68	NA
hsa-miR-2110	URS0000279B6F_9606	-0,224903351	-1,168698953	0,68	0,920034518
hsa-miR-942-5p	URS00005194EC_9606	0,280031081	1,214221043	0,68	0,920034518
hsa-miR-625-5p	URS00001F9866_9606	-0,270744258	-1,206430041	0,68	NA
hsa-miR-222-3p	URS00002C6949_9606	-0,219677859	-1,164473541	0,69	0,920520823
hsa-miR-142-3p	URS00002620A7_9606	-0,331846121	-1,258622921	0,69	0,920520823
hsa-miR-550a-3-5p	URS000050B2E2_9606	-0,438319653	-1,355025172	0,69	NA
hsa-miR-4685-3p	URS00001A5096_9606	-0,406272821	-1,325257607	0,69	NA
hsa-miR-181d-3p	URS000075C355_9606	-0,405899165	-1,324914411	0,69	NA
hsa-miR-4525	URS00004E02F2_9606	-0,405899165	-1,324914411	0,69	NA
hsa-miR-1278	URS0000171FEC_9606	-0,405899165	-1,324914411	0,69	NA
hsa-miR-4689	URS000011A030_9606	-0,405767358	-1,32479337	0,69	NA
hsa-miR-8485	URS000076B539_9606	-0,405767358	-1,32479337	0,69	NA
hsa-miR-20b-5p	URS00002B3783_9606	0,315065229	1,2440679	0,69	0,92308004
hsa-miR-545-3p	URS00002E1509_9606	-0,297222429	-1,228776414	0,70	NA
hsa-miR-660-3p	URS00000011DF_9606	0,28045498	1,214577863	0,70	NA
hsa-let-7d-5p	URS00000A07C1_9606	-0,167182061	-1,122863113	0,70	0,9305246
hsa-miR-888-5p	URS000075D73F_9606	-0,281659889	-1,215592677	0,70	0,9305246
hsa-miR-3913-3p	URS0000242881_9606	0,441521157	1,358035466	0,71	NA
hsa-miR-548b-3p	URS000039A25B_9606	-0,29629926	-1,227990381	0,71	NA
hsa-miR-128-3p	URS000024A59E_9606	-0,239684537	-1,180734451	0,71	0,933236559
hsa-miR-488-5p	URS00002A36ED_9606	0,307624992	1,237668535	0,71	NA
hsa-miR-6883-3p	URS000075E11F_9606	-0,50553887	-1,41965352	0,71	NA
hsa-miR-550a-3p	URS00001F14FE_9606	0,546801318	1,460843184	0,71	NA
hsa-miR-1294	URS00001C85C7_9606	0,313918189	1,243079175	0,72	0,940470952
hsa-miR-425-3p	URS000056B04E_9606	-0,224003774	-1,16797045	0,72	0,940470952

hsa-miR-10399-5p	URS0000D4FABD_9606	-0,298760086	-1,23008677	0,72	NA
hsa-miR-1268a	URS00005A8A8D_9606	0,300000706	1,231145016	0,72	NA
hsa-miR-590-5p	URS00005CACA0_9606	-0,356007816	-1,279879348	0,72	NA
hsa-let-7b-3p	URS00005918D5_9606	0,2154001	1,161025855	0,73	0,940470952
hsa-miR-548e-5p	URS000077B07D_9606	-0,253019031	-1,191698295	0,73	0,940470952
hsa-miR-3187-3p	URS00005E640C_9606	-0,219254771	-1,164132095	0,73	NA
hsa-miR-675-5p	URS00004E5112_9606	-0,264702343	-1,201388154	0,73	NA
hsa-miR-99a-5p	URS0000157026_9606	0,167160851	1,122846605	0,74	0,940470952
hsa-miR-3605-3p	URS00000A9FB9_9606	-0,299059742	-1,230342292	0,74	0,940470952
hsa-miR-16-1-3p	URS000061CB8F_9606	-0,401542538	-1,320919491	0,74	NA
hsa-miR-643	URS00001941B8_9606	-0,302244998	-1,233061706	0,74	NA
hsa-miR-4636	URS00000E732F_9606	0,282574519	1,216363575	0,74	NA
hsa-miR-3118	URS00001EE4D9_9606	-0,301199307	-1,232168285	0,74	NA
hsa-miR-95-3p	URS00002E2DE7_9606	0,166034575	1,121970369	0,74	0,940470952
hsa-miR-708-3p	URS000049EEDF_9606	-0,182983853	-1,135229396	0,74	0,940470952
hsa-miR-501-5p	URS00001E2DBC_9606	0,309187494	1,23900971	0,74	NA
hsa-miR-5009-5p	URS0000482389_9606	-0,301462762	-1,232393315	0,74	NA
hsa-miR-7113-5p	URS00007599A0_9606	-0,301462762	-1,232393315	0,74	NA
hsa-miR-3614-3p	URS000042BE4B_9606	-0,301462762	-1,232393315	0,74	NA
hsa-miR-5193	URS0000224AEA_9606	0,460709736	1,376218685	0,74	NA
hsa-miR-6780b-3p	URS000075C2D2_9606	0,460709736	1,376218685	0,74	NA
hsa-miR-3943	URS000075EDCB_9606	0,460709736	1,376218685	0,74	NA
hsa-miR-3620-5p	URS00004818BD_9606	0,460709736	1,376218685	0,74	NA
hsa-miR-1226-5p	URS000075EAB0_9606	0,460709736	1,376218685	0,74	NA
hsa-miR-4684-5p	URS00003570D1_9606	0,460709736	1,376218685	0,74	NA
hsa-miR-33a-3p	URS00003E3B82_9606	0,460709736	1,376218685	0,74	NA
hsa-miR-1249-3p	URS000060AABB_9606	0,460709736	1,376218685	0,74	NA
hsa-miR-615-5p	URS00004D8280_9606	0,460698207	1,376207687	0,74	NA
hsa-miR-376a-2-5p	URS00004B1322_9606	0,460698207	1,376207687	0,74	NA
hsa-miR-6134	URS000075DB54_9606	0,460698207	1,376207687	0,74	NA
hsa-miR-6726-5p	URS000075C413_9606	0,460698207	1,376207687	0,74	NA
hsa-miR-194-3p	URS0000065566_9606	0,460698207	1,376207687	0,74	NA
hsa-miR-6803-3p	URS000075E2A1_9606	0,460698207	1,376207687	0,74	NA
hsa-miR-3680-3p	URS000075F058_9606	0,460135597	1,37567111	0,74	NA
hsa-miR-3606-5p	URS00003AFAD1_9606	0,460135597	1,37567111	0,74	NA
hsa-miR-6755-3p	URS000075CB56_9606	0,460135597	1,37567111	0,74	NA
hsa-miR-597-3p	URS000075E29A_9606	0,460135597	1,37567111	0,74	NA
hsa-miR-7854-3p	URS000075A4C3_9606	0,460135597	1,37567111	0,74	NA
hsa-miR-4524a-3p	URS0000475586_9606	0,460135597	1,37567111	0,74	NA

hsa-miR-627-3p	URS000075D437_9606	0,460135597	1,37567111	0,74	NA
hsa-miR-550b-3p	URS000031847A_9606	0,460135597	1,37567111	0,74	NA
hsa-miR-2681-3p	URS00002FA7A6_9606	0,460135597	1,37567111	0,74	NA
hsa-miR-4485-3p	URS000038446A_9606	0,460135597	1,37567111	0,74	NA
hsa-miR-487a-5p	URS000075BB46_9606	0,460135597	1,37567111	0,74	NA
hsa-miR-5571-3p	URS000023F8DA_9606	0,460135597	1,37567111	0,74	NA
hsa-miR-4526	URS0000009265_9606	0,460135597	1,37567111	0,74	NA
hsa-miR-3680-5p	URS00005EAC45_9606	0,460135597	1,37567111	0,74	NA
hsa-miR-578	URS000075B968_9606	0,460135597	1,37567111	0,74	NA
hsa-miR-3161	URS00000D5659_9606	0,460135597	1,37567111	0,74	NA
hsa-miR-6845-5p	URS000075CFFD_9606	0,460135597	1,37567111	0,74	NA
hsa-miR-3910	URS00003E1C37_9606	0,460135597	1,37567111	0,74	NA
hsa-miR-4685-5p	URS00000219AD_9606	0,460135597	1,37567111	0,74	NA
hsa-miR-499a-3p	URS000075BB59_9606	0,460135597	1,37567111	0,74	NA
hsa-miR-4712-3p	URS000058FFA6_9606	0,460135597	1,37567111	0,74	NA
hsa-miR-6800-3p	URS000075EE88_9606	0,460135597	1,37567111	0,74	NA
hsa-miR-3691-3p	URS000053E475_9606	0,460135597	1,37567111	0,74	NA
hsa-miR-6501-5p	URS000075C286_9606	0,460135597	1,37567111	0,74	NA
hsa-miR-219b-3p	URS0000603CA3_9606	0,460135597	1,37567111	0,74	NA
hsa-miR-4747-5p	URS0000558A72_9606	0,460135597	1,37567111	0,74	NA
hsa-miR-4511	URS000075E8E6_9606	0,460135597	1,37567111	0,74	NA
hsa-miR-3619-3p	URS000061BAF5_9606	0,460135597	1,375671109	0,74	NA
hsa-miR-4767	URS000035D2F9_9606	0,460135597	1,375671109	0,74	NA
hsa-miR-4729	URS000024F41A_9606	0,460135597	1,375671109	0,74	NA
hsa-miR-3938	URS000075EB68_9606	0,460003728	1,375545373	0,74	NA
hsa-miR-3679-3p	URS00003E3F4E_9606	0,460003728	1,375545373	0,74	NA
hsa-miR-4755-5p	URS0000429226_9606	0,460003728	1,375545373	0,74	NA
hsa-miR-7156-5p	URS000075B2E1_9606	0,460003728	1,375545373	0,74	NA
hsa-miR-605-3p	URS0000785093_9606	0,460003728	1,375545373	0,74	NA
hsa-miR-4754	URS000058C0AB_9606	0,460003728	1,375545373	0,74	NA
hsa-miR-6513-5p	URS000075C1E1_9606	0,460003728	1,375545373	0,74	NA
hsa-miR-4659b-3p	URS0000327F71_9606	0,460003728	1,375545373	0,74	NA
hsa-miR-5695	URS000075D1F5_9606	0,460003727	1,375545371	0,74	NA
hsa-miR-5689	URS000075DECF_9606	0,460003727	1,375545371	0,74	NA
hsa-miR-4707-3p	URS000018D2BE_9606	0,460003727	1,375545371	0,74	NA
hsa-miR-5192	URS0000759A15_9606	0,460003727	1,375545371	0,74	NA
hsa-miR-4757-5p	URS00000A8EBA_9606	0,460003727	1,375545371	0,74	NA
hsa-miR-3529-5p	URS00004F24A9_9606	0,460003727	1,375545371	0,74	NA
hsa-miR-4795-3p	URS0000490308_9606	0,460003727	1,375545371	0,74	NA

hsa-miR-1263	URS000075E8EC_9606	0,460003727	1,375545371	0,74	NA
hsa-miR-1264	URS0000759E8E_9606	0,460003727	1,375545371	0,74	NA
hsa-miR-6753-5p	URS000075EF1E_9606	0,460003727	1,375545371	0,74	NA
hsa-miR-4676-3p	URS0000200A0B_9606	0,460003727	1,375545371	0,74	NA
hsa-miR-6874-3p	URS000075B25C_9606	0,460003727	1,375545371	0,74	NA
hsa-miR-1298-3p	URS000075B3DF_9606	0,460003727	1,375545371	0,74	NA
hsa-miR-6715a-3p	URS0000552FF1_9606	0,460003727	1,375545371	0,74	NA
hsa-miR-4733-3p	URS0000482D79_9606	0,460003727	1,375545371	0,74	NA
hsa-miR-6798-5p	URS000075A1E5_9606	0,460003727	1,375545371	0,74	NA
hsa-miR-3189-3p	URS00001B12A4_9606	0,460003727	1,375545371	0,74	NA
hsa-miR-1227-3p	URS000075CFA8_9606	0,460003727	1,375545371	0,74	NA
hsa-miR-887-5p	URS000075B4E7_9606	0,460003727	1,375545371	0,74	NA
hsa-miR-3074-3p	URS000046F124_9606	0,460003727	1,375545371	0,74	NA
hsa-miR-6825-3p	URS000075D5CF_9606	0,460003727	1,375545371	0,74	NA
hsa-miR-7158-5p	URS00007E381F_9606	0,460003727	1,375545371	0,74	NA
hsa-miR-572	URS000075CEB8_9606	0,460003727	1,375545371	0,74	NA
hsa-miR-5582-3p	URS000075D126_9606	0,460003727	1,375545371	0,74	NA
hsa-miR-1276	URS000042619A_9606	0,460003727	1,375545371	0,74	NA
hsa-miR-6783-5p	URS000075AA32_9606	0,460003727	1,375545371	0,74	NA
hsa-miR-4742-5p	URS0000473E7A_9606	0,460003727	1,375545371	0,74	NA
hsa-miR-6810-3p	URS000075A2D7_9606	0,460003727	1,375545371	0,74	NA
hsa-miR-3131	URS00000FB5B8_9606	0,460003727	1,375545371	0,74	NA
hsa-miR-6774-3p	URS000075DE96_9606	0,460003727	1,375545371	0,74	NA
hsa-miR-4700-5p	URS0000334286_9606	0,460003727	1,375545371	0,74	NA
hsa-miR-4774-5p	URS000055153D_9606	0,460003727	1,375545371	0,74	NA
hsa-miR-6859-3p	URS000075C233_9606	0,460003727	1,375545371	0,74	NA
hsa-miR-6797-3p	URS000075AB1D_9606	0,460003727	1,375545371	0,74	NA
hsa-miR-6781-3p	URS000075AB22_9606	0,460003727	1,375545371	0,74	NA
hsa-miR-4745-3p	URS000054DB95_9606	0,460003727	1,375545371	0,74	NA
hsa-miR-4728-5p	URS00003067E3_9606	0,460003727	1,375545371	0,74	NA
hsa-miR-1293	URS00001DABC0_9606	0,460003727	1,375545371	0,74	NA
hsa-miR-3138	URS00002C83DA_9606	0,460003727	1,375545371	0,74	NA
hsa-miR-4753-3p	URS00003AA845_9606	0,460003727	1,375545371	0,74	NA
hsa-miR-4709-3p	URS000023133F_9606	0,460003727	1,375545371	0,74	NA
hsa-miR-5094	URS000075BFDA_9606	0,460003727	1,375545371	0,74	NA
hsa-miR-5188	URS00004663C8_9606	0,460003727	1,375545371	0,74	NA
hsa-miR-6785-3p	URS000075A0B8_9606	0,460003727	1,375545371	0,74	NA
hsa-miR-139-3p	URS000023BE29_9606	-0,250781395	-1,18985139	0,74	0,940470952
hsa-miR-500a-3p	URS00003F659E_9606	-0,214507577	-1,160307809	0,75	0,940470952

hsa-miR-26a-2-3p	URS000015D23B_9606	-0,1706323	-1,12555168	0,75	0,940470952
hsa-miR-769-3p	URS00005F5472_9606	0,198348595	1,147384231	0,75	NA
hsa-miR-616-3p	URS00005E3F32_9606	-0,378760416	-1,300224204	0,75	NA
hsa-miR-3124-5p	URS000018CD09_9606	-0,298736916	-1,230067015	0,75	NA
hsa-miR-451a	URS00002E857A_9606	-0,190541637	-1,141192078	0,75	0,940470952
hsa-miR-16-2-3p	URS00001E9CCB_9606	0,212501937	1,158695866	0,75	0,940470952
hsa-miR-301a-5p	URS000020C95B_9606	-0,208951214	-1,15584762	0,75	NA
hsa-miR-1301-3p	URS00000FC8EB_9606	-0,168499105	-1,123888649	0,75	0,940470952
hsa-miR-6866-3p	URS000075E526_9606	0,352633976	1,276889761	0,76	NA
hsa-miR-3617-5p	URS000012846D_9606	0,352622443	1,276879553	0,76	NA
hsa-miR-18a-3p	URS00004131FE_9606	-0,218955162	-1,163890361	0,76	0,946251572
hsa-miR-3926	URS0000576DFF_9606	0,352191685	1,276498361	0,76	NA
hsa-miR-6505-5p	URS000075D8DA_9606	0,352191685	1,276498361	0,76	NA
hsa-miR-6740-5p	URS000075E833_9606	0,352191685	1,276498361	0,76	NA
hsa-miR-3174	URS0000243FF1_9606	0,352059878	1,276381743	0,76	NA
hsa-miR-5696	URS000075A304_9606	0,352059878	1,276381743	0,76	NA
hsa-miR-618	URS0000450F92_9606	0,352059878	1,276381743	0,76	NA
hsa-miR-4520-3p	URS00002A0C10_9606	0,352059878	1,276381743	0,76	NA
hsa-miR-503-3p	URS00003C11EC_9606	0,352059878	1,276381743	0,76	NA
hsa-miR-548ax	URS000075D311_9606	0,352059878	1,276381743	0,76	NA
hsa-miR-149-3p	URS000042C6A6_9606	0,352059878	1,276381743	0,76	NA
hsa-miR-3682-3p	URS0000072E4C_9606	0,352059878	1,276381743	0,76	NA
hsa-miR-548ar-5p	URS000075B9A9_9606	0,352059878	1,276381743	0,76	NA
hsa-miR-6741-3p	URS000075D658_9606	0,351839133	1,276186461	0,76	NA
hsa-miR-579-3p	URS00001B0D1E_9606	0,351928083	1,276265146	0,76	NA
hsa-miR-548az-3p	URS000075BC98_9606	0,351928083	1,276265146	0,76	NA
hsa-miR-4450	URS000005E58C_9606	0,351928083	1,276265146	0,76	NA
hsa-miR-548an	URS000075DD84_9606	0,351928083	1,276265146	0,76	NA
hsa-miR-3145-5p	URS00002D2333_9606	0,351718807	1,276080027	0,76	NA
hsa-miR-6818-3p	URS000075D9FB_9606	0,351707274	1,276069826	0,76	NA
hsa-miR-10395-3p	URS0000D52042_9606	0,351553645	1,275933947	0,76	NA
hsa-miR-548d-3p	URS000039FBF7_9606	0,351012914	1,275455809	0,76	NA
hsa-miR-6777-3p	URS000075E320_9606	0,351012914	1,275455809	0,76	NA
hsa-miR-2276-3p	URS000075A253_9606	0,351012914	1,275455809	0,76	NA
hsa-miR-15b-5p	URS00004AD914_9606	-0,150590358	-1,110023606	0,76	0,946251572
hsa-miR-548ap-5p	URS000054B69F_9606	-0,29816865	-1,229582596	0,77	NA
hsa-miR-580-3p	URS000075D9A5_9606	0,460135597	1,37567111	0,77	NA
hsa-miR-338-5p	URS00003776C9_9606	0,460135597	1,37567111	0,77	NA
hsa-miR-6510-3p	URS000075D4B1_9606	-0,366078669	-1,288844908	0,77	NA

hsa-miR-6793-5p	URS000075C6F8_9606	0,29650266	1,228163523	0,77	NA
hsa-miR-106b-5p	URS00004449AE_9606	0,21272664	1,158876349	0,77	0,946251572
hsa-miR-548t-5p	URS000057CDAB_9606	0,295719629	1,227497111	0,77	NA
hsa-miR-4664-3p	URS000044EF2B_9606	0,295593426	1,227389738	0,77	NA
hsa-miR-6820-5p	URS000075E8D5_9606	0,296766139	1,228387843	0,77	NA
hsa-miR-92b-3p	URS000025576D_9606	0,165882743	1,121852297	0,77	0,946251572
hsa-miR-3065-3p	URS000029D793_9606	0,229108262	1,172110237	0,77	0,946251572
hsa-miR-3939	URS000075E44E_9606	0,460724622	1,376232885	0,77	NA
hsa-miR-664b-3p	URS000057B50A_9606	0,261732517	1,198917608	0,77	NA
hsa-miR-6788-5p	URS00007E4C50_9606	0,261628339	1,198831036	0,77	NA
hsa-miR-3145-3p	URS00004060DD_9606	0,222661405	1,166884206	0,77	NA
hsa-miR-548ak	URS000037B2E0_9606	0,238091144	1,179431102	0,78	NA
hsa-miR-3168	URS000020C393_9606	0,351697038	1,276060771	0,78	NA
hsa-miR-6729-3p	URS000075D5F0_9606	0,353317148	1,27749456	0,78	NA
hsa-miR-4748	URS00001FA076_9606	-0,233100901	-1,17535853	0,78	NA
hsa-miR-92b-5p	URS00001A7F58_9606	-0,231533731	-1,174082455	0,78	0,949713027
hsa-miR-676-5p	URS000075F0C5_9606	-0,301462762	-1,232393315	0,78	NA
hsa-miR-3065-5p	URS00003FB16B_9606	0,168364122	1,123783499	0,79	0,949713027
hsa-miR-331-5p	URS00001597DC_9606	0,165846341	1,12182399	0,79	NA
hsa-miR-4690-3p	URS00005C8A12_9606	0,304735025	1,235191753	0,79	NA
hsa-miR-15b-3p	URS000045A9D7_9606	-0,209795901	-1,156524558	0,79	0,949713027
hsa-miR-4740-5p	URS0000521852_9606	0,296413999	1,228088048	0,79	NA
hsa-miR-142-5p	URS00001E0AEA_9606	-0,140989866	-1,102661418	0,79	0,949713027
hsa-miR-6511a-5p	URS000075C82B_9606	0,297891617	1,229346509	0,79	NA
hsa-miR-5001-3p	URS0000483D37_9606	0,238349098	1,179642003	0,79	NA
hsa-miR-29b-2-5p	URS0000403C02_9606	-0,174682881	-1,128716274	0,79	0,949713027
hsa-miR-545-5p	URS00004C4520_9606	0,263626585	1,200492662	0,79	NA
hsa-miR-221-5p	URS0000593537_9606	0,245476571	1,185484312	0,79	0,949713027
hsa-miR-181d-5p	URS0000236310_9606	-0,144258328	-1,105162357	0,79	0,949713027
hsa-miR-423-3p	URS00000BE495_9606	-0,142327868	-1,103684536	0,79	0,949713027
hsa-miR-6791-3p	URS000075A0CB_9606	0,35227584	1,276572824	0,79	NA
hsa-miR-1295a	URS00005CE113_9606	-0,194950422	-1,144684823	0,80	NA
hsa-miR-887-3p	URS00002A8BC6_9606	0,139852052	1,101792122	0,80	0,951767546
hsa-miR-2277-3p	URS00003CA515_9606	0,220188594	1,164885854	0,80	NA
hsa-miR-29b-1-5p	URS00001123BD_9606	-0,253432652	-1,192040004	0,80	NA
hsa-miR-299-5p	URS000017DBB8_9606	0,26293227	1,199915049	0,80	NA
hsa-miR-4510	URS00005F1B8C_9606	-0,18937914	-1,140272897	0,80	NA
hsa-miR-3611	URS00002AB575_9606	-0,301462762	-1,232393315	0,80	NA
hsa-miR-6780a-5p	URS000075E96A_9606	-0,235911657	-1,177650674	0,80	NA

hsa-miR-502-3p	URS0000601CC4_9606	0,139167554	1,101269492	0,80	0,952680634
hsa-miR-3146	URS00003AAF6D_9606	0,261886237	1,19904536	0,80	NA
hsa-miR-4728-3p	URS00001FF3CD_9606	-0,294345043	-1,22632812	0,80	NA
hsa-miR-5187-5p	URS000036B845_9606	-0,246040627	-1,185947896	0,81	NA
hsa-miR-11400	URS0000D557C8_9606	0,350859284	1,275319995	0,81	NA
hsa-miR-216a-5p	URS0000318E24_9606	0,272444533	1,207852706	0,81	NA
hsa-miR-1306-3p	URS000041E780_9606	0,16852985	1,1239126	0,81	NA
hsa-miR-195-3p	URS0000476C64_9606	0,140738695	1,102469462	0,81	0,956537053
hsa-miR-301a-3p	URS00001C11BC_9606	0,206818663	1,154140341	0,81	0,956537053
hsa-miR-6514-5p	URS000075B538_9606	0,183526936	1,135656818	0,82	NA
hsa-miR-223-5p	URS0000485CBB_9606	-0,177021214	-1,130547191	0,82	0,956537053
hsa-miR-4436b-3p	URS0000499F46_9606	-0,182335331	-1,134719201	0,82	NA
hsa-miR-3125	URS0000081D1E_9606	-0,231541804	-1,174089025	0,82	NA
hsa-miR-1843	URS0000D53CED_9606	-0,148769575	-1,108623562	0,82	0,956537053
hsa-miR-125b-2-3p	URS00001925C1_9606	0,103079618	1,074063744	0,82	0,956537053
hsa-miR-30b-3p	URS00002152A8_9606	-0,240648924	-1,181523991	0,82	NA
hsa-miR-128-1-5p	URS0000537082_9606	-0,17598509	-1,129735539	0,82	NA
hsa-miR-378d	URS00001234DF_9606	-0,145852196	-1,106383999	0,82	0,956537053
hsa-miR-126-3p	URS00001F1DA8_9606	-0,145440398	-1,106068241	0,83	0,956537053
hsa-miR-95-5p	URS000075D912_9606	0,125417441	1,090823315	0,83	0,956537053
hsa-miR-218-2-3p	URS00001F9A0F_9606	-0,129049445	-1,093572937	0,83	NA
hsa-let-7b-5p	URS0000324096_9606	0,097685455	1,070055373	0,83	0,956537053
hsa-miR-3613-3p	URS00004EAE33_9606	0,184116537	1,136121033	0,83	NA
hsa-miR-149-5p	URS00001C770D_9606	0,155721199	1,113978353	0,84	0,956537053
hsa-let-7f-2-3p	URS00001C04A2_9606	-0,140171029	-1,102035753	0,84	NA
hsa-miR-2114-5p	URS000075C77C_9606	0,133157283	1,096691144	0,84	0,956537053
hsa-miR-424-5p	URS00000F0F49_9606	-0,125367443	-1,090785511	0,84	0,956537053
hsa-miR-203b-3p	URS00004A26B5_9606	-0,122121305	-1,088333948	0,84	NA
hsa-miR-6513-3p	URS000075C427_9606	0,200795926	1,14933226	0,84	NA
hsa-miR-378c	URS000025307A_9606	-0,139972661	-1,101884235	0,84	NA
hsa-miR-6809-3p	URS000075CC57_9606	-0,321175602	-1,249348185	0,84	NA
hsa-miR-6879-5p	URS000075A09C_9606	-0,321175602	-1,249348185	0,84	NA
hsa-miR-208a-3p	URS00000E5433_9606	-0,321175602	-1,249348185	0,84	NA
hsa-miR-6834-5p	URS000075DF58_9606	-0,321175602	-1,249348185	0,84	NA
hsa-miR-1185-1-3p	URS000016E46F_9606	-0,321175602	-1,249348185	0,84	NA
hsa-miR-1228-3p	URS0000100748_9606	-0,321175602	-1,249348185	0,84	NA
hsa-miR-4727-3p	URS000006A1CB_9606	-0,321175602	-1,249348185	0,84	NA
hsa-miR-4655-5p	URS0000576E6E_9606	-0,321175602	-1,249348185	0,84	NA
hsa-miR-934	URS000075BA4B_9606	-0,321175602	-1,249348185	0,84	NA

hsa-miR-1253	URS000075A7EC_9606	-0,321175602	-1,249348185	0,84	NA
hsa-miR-4445-5p	URS000075C53B_9606	-0,321175602	-1,249348185	0,84	NA
hsa-miR-3150b-5p	URS0000604DA6_9606	-0,321175602	-1,249348185	0,84	NA
hsa-miR-3944-3p	URS0000446855_9606	-0,321175602	-1,249348185	0,84	NA
hsa-miR-4797-3p	URS00004848FF_9606	-0,321175602	-1,249348185	0,84	NA
hsa-miR-6089	URS000075B63F_9606	-0,321175602	-1,249348185	0,84	NA
hsa-miR-5739	URS0000759A14_9606	-0,321175602	-1,249348185	0,84	NA
hsa-miR-10398-3p	URS0000D52BC4_9606	-0,321175602	-1,249348185	0,84	NA
hsa-miR-6782-3p	URS000075EF7F_9606	-0,321175602	-1,249348185	0,84	NA
hsa-miR-1228-5p	URS00004F1E01_9606	-0,321175602	-1,249348185	0,84	NA
hsa-miR-4424	URS000075A76A_9606	-0,321175602	-1,249348185	0,84	NA
hsa-miR-3130-5p	URS00005D7C80_9606	-0,321175602	-1,249348185	0,84	NA
hsa-miR-11181-3p	URS0000754CC5_9606	-0,321175602	-1,249348185	0,84	NA
hsa-miR-4800-3p	URS0000149319_9606	-0,321175602	-1,249348185	0,84	NA
hsa-miR-4520-5p	URS00001F8FF2_9606	-0,321175602	-1,249348185	0,84	NA
hsa-miR-6808-3p	URS0000759DC1_9606	-0,321175602	-1,249348185	0,84	NA
hsa-miR-6731-3p	URS000075C778_9606	-0,321175602	-1,249348185	0,84	NA
hsa-miR-18b-3p	URS00000A057E_9606	-0,321175602	-1,249348185	0,84	NA
hsa-miR-6732-3p	URS000075DD0B_9606	-0,321175602	-1,249348185	0,84	NA
hsa-miR-4802-3p	URS000004B7B7_9606	-0,321175602	-1,249348185	0,84	NA
hsa-miR-9898	URS0000D5252F_9606	-0,321175602	-1,249348185	0,84	NA
hsa-miR-3659	URS00004A6E26_9606	-0,321175602	-1,249348185	0,84	NA
hsa-miR-4533	URS0000759C6E_9606	-0,321175602	-1,249348185	0,84	NA
hsa-miR-3202	URS000005E91B_9606	-0,321175602	-1,249348185	0,84	NA
hsa-miR-3911	URS00001B968D_9606	-0,321175602	-1,249348185	0,84	NA
hsa-miR-3941	URS000075CA3B_9606	-0,321175602	-1,249348185	0,84	NA
hsa-miR-4659b-5p	URS0000208DDC_9606	-0,321175602	-1,249348185	0,84	NA
hsa-miR-7855-5p	URS000075D410_9606	-0,321175602	-1,249348185	0,84	NA
hsa-miR-12114	URS0000D528B4_9606	-0,321175602	-1,249348185	0,84	NA
hsa-miR-519c-5p	URS00005ECFE5_9606	-0,321175602	-1,249348185	0,84	NA
hsa-miR-1910-5p	URS00004F2EB0_9606	-0,321175602	-1,249348185	0,84	NA
hsa-miR-4270	URS00005E80AD_9606	-0,321175602	-1,249348185	0,84	NA
hsa-miR-6130	URS000075DDBE_9606	-0,321175602	-1,249348185	0,84	NA
hsa-miR-609	URS000075D046_9606	-0,321175602	-1,249348185	0,84	NA
hsa-miR-3622b-5p	URS000075E791_9606	-0,321175602	-1,249348185	0,84	NA
hsa-miR-4423-5p	URS0000567AB1_9606	-0,321175602	-1,249348185	0,84	NA
hsa-miR-377-5p	URS000036BEF1_9606	-0,321175602	-1,249348185	0,84	NA
hsa-miR-5584-5p	URS000075A332_9606	-0,321175602	-1,249348185	0,84	NA
hsa-miR-3179	URS00003AF4CD_9606	-0,321175602	-1,249348185	0,84	NA

hsa-miR-1224-3p	URS000075C165_9606	-0,321175602	-1,249348185	0,84	NA
hsa-miR-6801-3p	URS000075DFB9_9606	-0,321175602	-1,249348185	0,84	NA
hsa-miR-6880-3p	URS000075CCC6_9606	-0,321175602	-1,249348185	0,84	NA
hsa-miR-4778-5p	URS00000AAE32_9606	-0,321175602	-1,249348185	0,84	NA
hsa-miR-1237-5p	URS00003E1F0B_9606	-0,321175602	-1,249348185	0,84	NA
hsa-miR-7847-3p	URS00007E3A69_9606	-0,321175602	-1,249348185	0,84	NA
hsa-miR-4659a-5p	URS00004FD12F_9606	-0,321175602	-1,249348185	0,84	NA
hsa-miR-7702	URS000020BB16_9606	-0,321175602	-1,249348185	0,84	NA
hsa-miR-1238-3p	URS000075E57E_9606	-0,321175602	-1,249348185	0,84	NA
hsa-miR-103a-1-5p	URS00005812A9_9606	-0,321175602	-1,249348185	0,84	NA
hsa-miR-489-3p	URS000009C45F_9606	-0,187925571	-1,139124608	0,84	0,958062792
hsa-miR-7-5p	URS0000161522_9606	-0,118080044	-1,085289585	0,86	0,967618887
hsa-miR-1260b	URS0000239117_9606	-0,123096639	-1,089069964	0,86	0,967618887
hsa-miR-4508	URS00004E78D3_9606	-0,147218342	-1,107432174	0,86	0,967618887
hsa-miR-1262	URS0000568FF8_9606	-0,116155514	-1,083842792	0,86	NA
hsa-miR-664b-5p	URS000042B108_9606	-0,123980399	-1,089737307	0,87	NA
hsa-miR-3912-3p	URS0000472A9B_9606	0,204876365	1,152587569	0,87	NA
hsa-miR-296-5p	URS00001C3AC1_9606	-0,103032581	-1,074028727	0,87	0,967618887
hsa-miR-151a-5p	URS00005F8E5B_9606	0,120479939	1,087096445	0,87	0,967618887
hsa-miR-181a-5p	URS00003DA300_9606	0,076874623	1,054730652	0,87	0,967618887
hsa-let-7g-5p	URS00004AFF8D_9606	0,07881653	1,056151304	0,88	0,967618887
hsa-miR-378f	URS0000043B1D_9606	-0,07999443	-1,05701396	0,88	0,967618887
hsa-miR-326	URS00000A939F_9606	-0,085432334	-1,061005646	0,88	0,967618887
hsa-miR-4507	URS0000099F48_9606	-0,295815497	-1,227578681	0,88	NA
hsa-miR-548b-5p	URS000059E19C_9606	0,140226043	1,102077777	0,88	NA
hsa-miR-204-3p	URS000059A01D_9606	0,090473886	1,064719857	0,88	0,967618887
hsa-miR-181c-5p	URS000018C928_9606	0,104502523	1,075123597	0,88	0,967618887
hsa-miR-4716-3p	URS000020579D_9606	-0,116978679	-1,084461382	0,89	NA
hsa-miR-619-5p	URS000075B584_9606	-0,12274674	-1,088805862	0,89	NA
hsa-miR-6717-5p	URS000075A86E_9606	-0,286130998	-1,219365808	0,89	NA
hsa-miR-362-3p	URS00003A19A3_9606	-0,092706278	-1,066368654	0,89	0,967618887
hsa-miR-10399-3p	URS0000D501D5_9606	-0,080226354	-1,057183896	0,89	NA
hsa-miR-138-5p	URS000040780F_9606	0,099447371	1,071362995	0,89	0,967618887
hsa-miR-548ad-5p	URS000077CDCE_9606	-0,091618881	-1,065565207	0,89	0,967618887
hsa-miR-10a-5p	URS000016D2D4_9606	0,060442786	1,04278576	0,90	0,967618887
hsa-miR-654-3p	URS00002F40E9_9606	-0,086539975	-1,061820555	0,90	0,967618887
hsa-miR-2116-3p	URS00005237AB_9606	-0,170998501	-1,125837417	0,90	NA
hsa-miR-7975	URS000075D1BE_9606	-0,149426543	-1,109128517	0,90	NA
hsa-miR-217-5p	URS000041E210_9606	-0,133335893	-1,096826926	0,90	NA

hsa-miR-1229-3p	URS000075BB29_9606	0,149120785	1,108893479	0,90	NA
hsa-miR-6747-3p	URS000075B922_9606	-0,153051417	-1,111918785	0,90	NA
hsa-miR-6758-5p	URS000075A3BC_9606	-0,153023956	-1,111897621	0,90	NA
hsa-miR-548as-5p	URS000075A3D2_9606	-0,153023956	-1,111897621	0,90	NA
hsa-miR-6821-5p	URS000075EAF3_9606	-0,152919548	-1,111817155	0,90	NA
hsa-miR-6787-3p	URS000075CC55_9606	-0,152892087	-1,111795993	0,90	NA
hsa-miR-548ap-3p	URS0000578557_9606	-0,152897787	-1,111800385	0,90	NA
hsa-miR-6781-5p	URS000075A1B4_9606	-0,152897787	-1,111800385	0,90	NA
hsa-miR-933	URS0000425000_9606	-0,152897787	-1,111800385	0,90	NA
hsa-miR-376a-5p	URS000032A93F_9606	-0,152897787	-1,111800385	0,90	NA
hsa-miR-379-3p	URS00001EE123_9606	-0,152897787	-1,111800385	0,90	NA
hsa-miR-4646-5p	URS00001A6D0C_9606	-0,152897787	-1,111800385	0,90	NA
hsa-miR-3620-3p	URS000075C163_9606	-0,152897787	-1,111800385	0,90	NA
hsa-miR-5586-5p	URS0000759C15_9606	-0,152765919	-1,111698767	0,90	NA
hsa-miR-6754-3p	URS000075DFC4_9606	-0,152345409	-1,111374781	0,90	NA
hsa-miR-4428	URS000075CBFD_9606	-0,152203308	-1,11126532	0,90	NA
hsa-miR-6505-3p	URS000075DED1_9606	-0,152191779	-1,111256439	0,90	NA
hsa-miR-3942-5p	URS00005455A9_9606	-0,152191779	-1,111256439	0,90	NA
hsa-miR-6850-5p	URS000075EFCC_9606	-0,152191779	-1,111256439	0,90	NA
hsa-miR-6750-3p	URS000075CE3F_9606	-0,152191779	-1,111256439	0,90	NA
hsa-miR-3064-3p	URS0000047615_9606	-0,151982618	-1,111095342	0,90	NA
hsa-miR-3149	URS00005C1F22_9606	-0,151982618	-1,111095342	0,90	NA
hsa-miR-92a-1-5p	URS00001DA458_9606	-0,151982618	-1,111095342	0,90	NA
hsa-miR-4794	URS00003EAB10_9606	-0,151982618	-1,111095342	0,90	NA
hsa-miR-10401-3p	URS0000D54D27_9606	-0,151982618	-1,111095342	0,90	NA
hsa-miR-6793-3p	URS000075EFC6_9606	-0,151982618	-1,111095342	0,90	NA
hsa-miR-6806-3p	URS000075E3C0_9606	-0,151982618	-1,111095342	0,90	NA
hsa-miR-610	URS00004DC583_9606	-0,151982618	-1,111095342	0,90	NA
hsa-miR-6731-5p	URS000075BFCB_9606	-0,151982618	-1,111095342	0,90	NA
hsa-miR-4804-5p	URS0000325AF9_9606	-0,151982618	-1,111095342	0,90	NA
hsa-miR-642b-5p	URS000075B1CE_9606	-0,151982618	-1,111095342	0,90	NA
hsa-miR-6515-5p	URS000075EAB6_9606	-0,151982618	-1,111095342	0,90	NA
hsa-miR-6736-5p	URS000075A2B8_9606	-0,151982618	-1,111095342	0,90	NA
hsa-miR-4659a-3p	URS00003003B4_9606	-0,151982618	-1,111095342	0,90	NA
hsa-miR-942-3p	URS000075C646_9606	-0,151982618	-1,111095342	0,90	NA
hsa-miR-4731-3p	URS000015C522_9606	-0,151982618	-1,111095342	0,90	NA
hsa-miR-548at-3p	URS00007599F1_9606	-0,151982618	-1,111095342	0,90	NA
hsa-miR-642b-3p	URS0000453664_9606	-0,151982618	-1,111095342	0,90	NA
hsa-miR-4657	URS0000412AC7_9606	-0,151982618	-1,111095342	0,90	NA

hsa-miR-548w	URS00003A8BB2_9606	-0,151982618	-1,111095342	0,90	NA
hsa-miR-5008-5p	URS000075E3D9_9606	-0,151982618	-1,111095342	0,90	NA
hsa-miR-1908-3p	URS000075E4A7_9606	-0,151982618	-1,111095342	0,90	NA
hsa-miR-548y	URS000026875B_9606	-0,15185075	-1,110993788	0,90	NA
hsa-miR-187-5p	URS000047FD4C_9606	-0,15185075	-1,110993788	0,90	NA
hsa-miR-450a-2-3p	URS000075CA21_9606	-0,15185075	-1,110993788	0,90	NA
hsa-miR-182-3p	URS000075B447_9606	-0,15185075	-1,110993788	0,90	NA
hsa-miR-1255a	URS00003CC709_9606	-0,15185075	-1,110993788	0,90	NA
hsa-miR-3139	URS0000364FEF_9606	-0,15185075	-1,110993788	0,90	NA
hsa-miR-4769-5p	URS00004BBEBC_9606	-0,15185075	-1,110993788	0,90	NA
hsa-miR-3175	URS00002394F5_9606	-0,15185075	-1,110993788	0,90	NA
hsa-miR-1271-3p	URS00001D4A78_9606	-0,15185075	-1,110993788	0,90	NA
hsa-miR-6817-3p	URS000075E3B3_9606	-0,15185075	-1,110993788	0,90	NA
hsa-miR-7-2-3p	URS0000572E11_9606	-0,15185075	-1,110993788	0,90	NA
hsa-miR-3188	URS000049704B_9606	-0,15185075	-1,110993788	0,90	NA
hsa-miR-4644	URS000013207B_9606	-0,15185075	-1,110993788	0,90	NA
hsa-miR-4654	URS00002ED720_9606	-0,15185075	-1,110993788	0,90	NA
hsa-miR-412-5p	URS0000321A1A_9606	-0,15185075	-1,110993788	0,90	NA
hsa-miR-6785-5p	URS0000759E52_9606	-0,15185075	-1,110993788	0,90	NA
hsa-miR-943	URS000075DAD2_9606	-0,15185075	-1,110993788	0,90	NA
hsa-miR-6131	URS000075CF50_9606	-0,15128814	-1,110560616	0,90	NA
hsa-miR-3199	URS000031362A_9606	-0,15128814	-1,110560616	0,90	NA
hsa-miR-18b-5p	URS00004565E5_9606	-0,15128814	-1,110560616	0,90	NA
hsa-let-7f-1-3p	URS00002F8148_9606	0,095399403	1,068361137	0,90	0,967618887
hsa-miR-3614-5p	URS00003D4175_9606	-0,152356938	-1,111383663	0,91	NA
hsa-miR-191-3p	URS00002B2B5C_9606	0,11621153	1,083884876	0,91	NA
hsa-miR-5189-5p	URS00005CCD18_9606	-0,151982618	-1,111095342	0,91	NA
hsa-miR-185-5p	URS00004176D4_9606	-0,076383942	-1,054371984	0,91	0,967618887
hsa-miR-96-5p	URS000016FF9C_9606	-0,071522612	-1,050825133	0,91	0,967618887
hsa-miR-378a-3p	URS00000451A1_9606	0,050421021	1,035567089	0,91	0,967618887
hsa-miR-190b-5p	URS000075CB24_9606	-0,099658006	-1,071519426	0,91	0,967618887
hsa-miR-4683	URS00005956D9_9606	0,086134446	1,061522129	0,91	NA
hsa-miR-1224-5p	URS0000435A77_9606	0,076348302	1,054345937	0,92	NA
hsa-miR-323a-3p	URS00003CCAB4_9606	-0,072770929	-1,051734772	0,92	NA
hsa-miR-4746-5p	URS0000156390_9606	-0,151982618	-1,111095342	0,92	NA
hsa-miR-625-3p	URS0000475E09_9606	0,07331023	1,052128	0,92	0,967618887
hsa-miR-589-3p	URS00005F9DAE_9606	-0,106550202	-1,07665065	0,92	NA
hsa-miR-378i	URS0000480E22_9606	-0,050318463	-1,035493476	0,92	0,967618887
hsa-miR-1255b-5p	URS0000211070_9606	-0,104797648	-1,075343553	0,92	0,967618887

hsa-miR-28-3p	URS00001799A3_9606	0,048190644	1,03396736	0,92	0,967618887
hsa-miR-3059-5p	URS0000D5581B_9606	0,110456188	1,079569548	0,92	NA
hsa-miR-28-5p	URS00003E47B1_9606	0,065535678	1,046473427	0,93	0,967618887
hsa-miR-339-3p	URS000055B190_9606	-0,048778933	-1,034389068	0,93	0,967618887
hsa-miR-7977	URS000075A1F7_9606	0,066099563	1,046882527	0,93	0,967618887
hsa-miR-4536-5p	URS000075C6DC_9606	-0,112127609	-1,080820998	0,93	NA
hsa-miR-493-3p	URS00005E7CB2_9606	-0,113080569	-1,081535162	0,93	NA
hsa-miR-98-5p	URS00004E0808_9606	0,055074771	1,038912946	0,93	0,969483066
hsa-miR-429	URS000055BBE5_9606	0,042619967	1,029982601	0,94	0,969483066
hsa-miR-330-3p	URS000007A060_9606	-0,050735563	-1,035792892	0,94	NA
hsa-miR-145-5p	URS0000527F89_9606	0,049464688	1,03488086	0,94	0,969483066
hsa-miR-4479	URS000075D0D9_9606	-0,077231133	-1,054991323	0,94	NA
hsa-miR-329-5p	URS000075BD0A_9606	-0,076973123	-1,054802666	0,94	NA
hsa-miR-1915-3p	URS000039BFD2_9606	-0,078020286	-1,05556856	0,94	NA
hsa-miR-548g-5p	URS00004BC299_9606	-0,076321961	-1,054326687	0,94	NA
hsa-miR-3164	URS000042E8CC_9606	-0,076190166	-1,054230375	0,94	NA
hsa-miR-490-3p	URS00001496AE_9606	-0,076190166	-1,054230375	0,94	NA
hsa-miR-579-5p	URS0000759E88_9606	-0,076190166	-1,054230375	0,94	NA
hsa-miR-3157-3p	URS00004C5EF1_9606	-0,076190166	-1,054230375	0,94	NA
hsa-miR-4783-3p	URS00000B6B44_9606	-0,076190166	-1,054230375	0,94	NA
hsa-miR-937-3p	URS0000553F51_9606	-0,0756276	-1,053819368	0,94	NA
hsa-miR-27b-3p	URS000059311D_9606	0,026057388	1,018225702	0,94	0,969483066
hsa-miR-671-3p	URS00002B7450_9606	0,04374838	1,030788524	0,94	0,969483066
hsa-miR-1285-5p	URS000050A3A3_9606	0,066095058	1,046879258	0,94	NA
hsa-miR-6772-3p	URS0000759C64_9606	-0,077594422	-1,055257016	0,95	NA
hsa-miR-26a-1-3p	URS00000C0D3F_9606	-0,057096347	-1,040369743	0,95	NA
hsa-miR-211-5p	URS00001A3555_9606	-0,07771032	-1,055341793	0,95	NA
hsa-miR-548aq-3p	URS0000118224_9606	0,032371685	1,022691975	0,95	NA
hsa-miR-7-1-3p	URS000028D811_9606	-0,055516694	-1,039231232	0,95	NA
hsa-miR-3622a-5p	URS00003B59DF_9606	0,039845376	1,028003642	0,95	0,971335855
hsa-miR-181c-3p	URS0000244A71_9606	-0,033649254	-1,023598014	0,95	0,971335855
hsa-miR-330-5p	URS00003380C1_9606	0,033942879	1,023806364	0,95	NA
hsa-miR-409-3p	URS00002915C8_9606	0,055344654	1,039107312	0,96	0,971335855
hsa-miR-185-3p	URS00002367FA_9606	0,04769688	1,033613544	0,96	NA
hsa-miR-556-3p	URS00001D6605_9606	-0,056070353	-1,039630131	0,96	NA
hsa-miR-197-3p	URS000061E740_9606	0,049542815	1,034936904	0,96	0,971335855
hsa-miR-4785	URS0000266339_9606	0,061505397	1,043554102	0,96	NA
hsa-miR-9983-3p	URS0000D4FF80_9606	-0,056298175	-1,039794317	0,96	NA
hsa-miR-4738-3p	URS0000299FE3_9606	0,03733325	1,026215165	0,97	NA

hsa-miR-34a-3p	URS00000EED18_9606	-0,038362806	-1,026947768	0,97	0,978664926
hsa-miR-4433b-3p	URS000075CAA8_9606	-0,061960648	-1,043883453	0,97	NA
hsa-miR-378h	URS000033F054_9606	0,028450627	1,019916205	0,97	NA
hsa-miR-106a-5p	URS00003FE4D4_9606	-0,034378659	-1,024115661	0,97	NA
hsa-miR-4521	URS000034E58D_9606	-0,033988966	-1,02383907	0,97	NA
hsa-miR-6892-5p	URS000075CFDA_9606	-0,033074605	-1,02319038	0,97	NA
hsa-miR-24-3p	URS000059273E_9606	0,016377052	1,011416382	0,97	0,98315572
hsa-miR-340-3p	URS000048521E_9606	0,026928195	1,018840486	0,98	NA
hsa-miR-29b-3p	URS000024463E_9606	0,012427611	1,008651372	0,98	0,98641137
hsa-miR-210-3p	URS000055128B_9606	0,012237512	1,008518475	0,98	0,98641137
hsa-miR-3960	URS00003783AB_9606	0,018488241	1,012897537	0,99	0,98641137
hsa-miR-629-3p	URS00004065BA_9606	0,013183335	1,00917987	0,99	NA
hsa-miR-4677-3p	URS0000372130_9606	0,012373212	1,00861334	0,99	NA
hsa-miR-9903	URS0000D4F8CF_9606	-0,007152598	-1,004970113	0,99	NA
hsa-miR-548k	URS000038718E_9606	-0,006097907	-1,004235693	0,99	NA
hsa-miR-1292-5p	URS00005586D0_9606	-0,006793762	-1,004720182	0,99	NA
hsa-miR-4802-5p	URS000044D459_9606	-0,005455764	-1,003788807	0,99	NA
hsa-miR-212-3p	URS00001D6BAE_9606	-0,005324104	-1,003697205	1,00	NA
hsa-miR-6877-5p	URS000075AAB7_9606	-0,00461831	-1,003206298	1,00	NA
hsa-miR-30c-1-3p	URS0000237FB8_9606	0,002245494	1,00155767	1,00	NA
hsa-miR-181b-2-3p	URS0000764165_9606	0,002353543	1,001632683	1,00	NA
hsa-miR-553	URS0000759AC9_9606	NA	NA	NA	NA
hsa-miR-7844-5p	URS000075B7DF_9606	NA	NA	NA	NA
hsa-miR-548g-3p	URS000075C7C4_9606	NA	NA	NA	NA
hsa-miR-6835-3p	URS000075AE82_9606	NA	NA	NA	NA
hsa-miR-4422	URS00005606AA_9606	NA	NA	NA	NA
hsa-miR-4484	URS00001CC0F4_9606	NA	NA	NA	NA
hsa-miR-548bb-5p	URS00007E3B9B_9606	NA	NA	NA	NA
hsa-miR-548a-5p	URS00005F5B1B_9606	NA	NA	NA	NA
hsa-miR-548av-5p	URS000075AB65_9606	NA	NA	NA	NA
hsa-miR-548ah-5p	URS0000759B5E_9606	NA	NA	NA	NA
hsa-miR-3646	URS000075EC3B_9606	NA	NA	NA	NA
hsa-miR-522-3p	URS000075E661_9606	NA	NA	NA	NA
hsa-miR-122b-3p	URS000060BAA7_9606	NA	NA	NA	NA
hsa-miR-649	URS000075DD5B_9606	NA	NA	NA	NA
hsa-miR-5583-5p	URS000075CF82_9606	NA	NA	NA	NA
hsa-miR-606	URS00007599CB_9606	NA	NA	NA	NA
hsa-miR-4432	URS000075B6CF_9606	NA	NA	NA	NA
hsa-miR-4662a-3p	URS0000215483_9606	NA	NA	NA	NA

hsa-miR-3156-5p	URS0000064063_9606	NA	NA	NA	NA
hsa-miR-4662b	URS0000586024_9606	NA	NA	NA	NA
hsa-miR-498-3p	URS0000D5237C_9606	NA	NA	NA	NA
hsa-miR-518e-3p	URS000075E57D_9606	NA	NA	NA	NA
hsa-miR-651-3p	URS000075D3EF_9606	NA	NA	NA	NA
hsa-miR-541-5p	URS0000076E54_9606	NA	NA	NA	NA
hsa-miR-548ba	URS000075BACB_9606	NA	NA	NA	NA
hsa-miR-570-5p	URS000031D186_9606	NA	NA	NA	NA
hsa-miR-3974	URS000075BB54_9606	NA	NA	NA	NA
hsa-miR-4735-3p	URS0000051ADF_9606	NA	NA	NA	NA
hsa-miR-562	URS000075A5E2_9606	NA	NA	NA	NA
hsa-miR-3184-3p	URS000075F097_9606	NA	NA	NA	NA
hsa-miR-519b-3p	URS00003883FE_9606	NA	NA	NA	NA
hsa-miR-519a-3p	URS0000135E29_9606	NA	NA	NA	NA
hsa-miR-519c-3p	URS000037D7E5_9606	NA	NA	NA	NA
hsa-miR-372-3p	URS0000759ECA_9606	NA	NA	NA	NA
hsa-miR-520a-3p	URS0000101689_9606	NA	NA	NA	NA
hsa-miR-520b-3p	URS00000ED701_9606	NA	NA	NA	NA
hsa-miR-520c-3p	URS000049A7EB_9606	NA	NA	NA	NA
hsa-miR-520e-3p	URS00001A5F54_9606	NA	NA	NA	NA
hsa-miR-520d-3p	URS000075D962_9606	NA	NA	NA	NA
hsa-miR-4517	URS00003FA24D_9606	NA	NA	NA	NA
hsa-miR-4506	URS000075D2D9_9606	NA	NA	NA	NA
hsa-miR-4760-3p	URS000018370B_9606	NA	NA	NA	NA
hsa-miR-3529-3p	URS000075C41F_9606	NA	NA	NA	NA
hsa-miR-6757-3p	URS000075AEFD_9606	NA	NA	NA	NA
hsa-miR-552-3p	URS000075A6FD_9606	NA	NA	NA	NA
hsa-miR-499b-3p	URS000023B5A7_9606	NA	NA	NA	NA
hsa-miR-1255b-2-3p	URS000075EDF2_9606	NA	NA	NA	NA
hsa-miR-634	URS000075CE29_9606	NA	NA	NA	NA
hsa-miR-5195-5p	URS00007E4C71_9606	NA	NA	NA	NA
hsa-miR-6813-3p	URS000075D02D_9606	NA	NA	NA	NA
hsa-miR-521	URS00001DBB42_9606	NA	NA	NA	NA
hsa-miR-122-3p	URS000075BAE6_9606	NA	NA	NA	NA
hsa-miR-548al	URS00004526F6_9606	NA	NA	NA	NA
hsa-miR-4681	URS000014FEF7_9606	NA	NA	NA	NA
hsa-miR-3923	URS000075D491_9606	NA	NA	NA	NA
hsa-miR-4798-3p	URS0000339954_9606	NA	NA	NA	NA
hsa-miR-5684	URS000075BF22_9606	NA	NA	NA	NA

hsa-miR-4714-5p	URS00005DA9AC_9606	NA	NA	NA	NA
hsa-miR-3664-5p	URS000075CCEC_9606	NA	NA	NA	NA
hsa-miR-4724-5p	URS00003CCBB9_9606	NA	NA	NA	NA
hsa-miR-1243	URS000075E096_9606	NA	NA	NA	NA
hsa-miR-5197-3p	URS000075CFA3_9606	NA	NA	NA	NA
hsa-miR-4499	URS0000759ABB_9606	NA	NA	NA	NA
hsa-miR-3925-5p	URS0000249054_9606	NA	NA	NA	NA
hsa-miR-6856-5p	URS000075A8A4_9606	NA	NA	NA	NA
hsa-miR-3916	URS00001310DE_9606	NA	NA	NA	NA
hsa-miR-6838-5p	URS000075EF04_9606	NA	NA	NA	NA
hsa-miR-646	URS0000759ED4_9606	NA	NA	NA	NA
hsa-miR-6779-3p	URS000075B933_9606	NA	NA	NA	NA
hsa-miR-129-2-3p	URS000048F59D_9606	NA	NA	NA	NA
hsa-miR-129-1-3p	URS00004CCDA3_9606	NA	NA	NA	NA
hsa-miR-6819-3p	URS000075B71F_9606	NA	NA	NA	NA
hsa-miR-596	URS000075B35F_9606	NA	NA	NA	NA
hsa-miR-208b-5p	URS000075AC54_9606	NA	NA	NA	NA
hsa-miR-6888-5p	URS000075DC2D_9606	NA	NA	NA	NA
hsa-miR-4747-3p	URS0000047A8F_9606	NA	NA	NA	NA
hsa-miR-3142	URS000075C0E1_9606	NA	NA	NA	NA
hsa-miR-9500	URS00007E3D21_9606	NA	NA	NA	NA
hsa-miR-4739	URS00002578DA_9606	NA	NA	NA	NA
hsa-miR-6743-5p	URS0000759F68_9606	NA	NA	NA	NA
hsa-miR-6085	URS000075CC84_9606	NA	NA	NA	NA
hsa-miR-4678	URS00005C0339_9606	NA	NA	NA	NA
hsa-miR-4464	URS00000C36BD_9606	NA	NA	NA	NA
hsa-miR-1244	URS000075B58F_9606	NA	NA	NA	NA
hsa-miR-5588-3p	URS000075EB8A_9606	NA	NA	NA	NA
hsa-miR-6838-3p	URS000075CEB9_9606	NA	NA	NA	NA
hsa-miR-1245a	URS000075D2F5_9606	NA	NA	NA	NA
hsa-miR-371b-3p	URS00004DFAD6_9606	NA	NA	NA	NA
hsa-miR-371a-3p	URS000075B452_9606	NA	NA	NA	NA
hsa-miR-519e-3p	URS00004F4C18_9606	NA	NA	NA	NA
hsa-miR-512-3p	URS000020F110_9606	NA	NA	NA	NA
hsa-miR-520f-3p	URS000000E601_9606	NA	NA	NA	NA
hsa-miR-648	URS000075E498_9606	NA	NA	NA	NA
hsa-miR-4715-5p	URS00001D8537_9606	NA	NA	NA	NA
hsa-miR-5590-3p	URS000075D290_9606	NA	NA	NA	NA
hsa-miR-5692b	URS000075CF33_9606	NA	NA	NA	NA

hsa-miR-5692c	URS000075B4F4_9606	NA	NA	NA	NA
hsa-miR-1295b-3p	URS000075AF37_9606	NA	NA	NA	NA
hsa-miR-154-3p	URS00000C0921_9606	NA	NA	NA	NA
hsa-miR-96-3p	URS00005176F0_9606	NA	NA	NA	NA
hsa-miR-5092	URS000075D27D_9606	NA	NA	NA	NA
hsa-miR-889-5p	URS000075AAF0_9606	NA	NA	NA	NA
hsa-miR-4495	URS0000759A3B_9606	NA	NA	NA	NA
hsa-miR-3668	URS000075C07E_9606	NA	NA	NA	NA
hsa-miR-4491	URS0000759EB8_9606	NA	NA	NA	NA
hsa-miR-511-3p	URS0000780BB8_9606	NA	NA	NA	NA
hsa-miR-4307	URS000075EC2B_9606	NA	NA	NA	NA
hsa-miR-367-3p	URS00001D5AA3_9606	NA	NA	NA	NA
hsa-miR-4699-3p	URS00004A3E7E_9606	NA	NA	NA	NA
hsa-miR-5002-5p	URS0000759CD5_9606	NA	NA	NA	NA
hsa-miR-3973	URS000075AC8C_9606	NA	NA	NA	NA
hsa-miR-520h	URS000059C31C_9606	NA	NA	NA	NA
hsa-miR-520g-3p	URS0000021D08_9606	NA	NA	NA	NA
hsa-miR-4761-5p	URS00002F924F_9606	NA	NA	NA	NA
hsa-miR-216b-3p	URS000075ABB0_9606	NA	NA	NA	NA
hsa-miR-3681-3p	URS000075E1C5_9606	NA	NA	NA	NA
hsa-miR-4717-3p	URS0000597BCD_9606	NA	NA	NA	NA
hsa-miR-6865-3p	URS000075BA09_9606	NA	NA	NA	NA
hsa-miR-6760-3p	URS000075EC62_9606	NA	NA	NA	NA
hsa-miR-499b-5p	URS0000409262_9606	NA	NA	NA	NA
hsa-miR-383-3p	URS000075B780_9606	NA	NA	NA	NA
hsa-miR-5685	URS000075B307_9606	NA	NA	NA	NA
hsa-miR-4433a-3p	URS00005A3BC9_9606	NA	NA	NA	NA
hsa-miR-6715b-5p	URS000075F0AC_9606	NA	NA	NA	NA
hsa-miR-4514	URS000075CDC6_9606	NA	NA	NA	NA
hsa-miR-3937	URS000075BC71_9606	NA	NA	NA	NA
hsa-miR-4441	URS000075BE81_9606	NA	NA	NA	NA
hsa-miR-3918	URS00004AD450_9606	NA	NA	NA	NA
hsa-miR-9900	URS0000D5155D_9606	NA	NA	NA	NA
hsa-miR-936	URS000075A073_9606	NA	NA	NA	NA
hsa-miR-622	URS000075E944_9606	NA	NA	NA	NA
hsa-miR-1-5p	URS000075C105_9606	NA	NA	NA	NA
hsa-miR-4793-5p	URS0000097EFE_9606	NA	NA	NA	NA
hsa-miR-4317	URS00005977B9_9606	NA	NA	NA	NA
hsa-miR-1250-3p	URS00007605DA_9606	NA	NA	NA	NA

hsa-miR-6849-3p	URS000075C7D0_9606	NA	NA	NA	NA
hsa-miR-4738-5p	URS00000CA292_9606	NA	NA	NA	NA
hsa-miR-6837-5p	URS000075DBA0_9606	NA	NA	NA	NA
hsa-miR-4726-3p	URS0000528E50_9606	NA	NA	NA	NA
hsa-miR-6840-5p	URS000075C1B6_9606	NA	NA	NA	NA
hsa-miR-6795-3p	URS000075E1D2_9606	NA	NA	NA	NA
hsa-miR-7108-3p	URS000075BD2F_9606	NA	NA	NA	NA
hsa-miR-4655-3p	URS00003B66C6_9606	NA	NA	NA	NA
hsa-miR-4780	URS00000F1728_9606	NA	NA	NA	NA
hsa-miR-6832-3p	URS000075E203_9606	NA	NA	NA	NA
hsa-miR-4671-5p	URS00002AA689_9606	NA	NA	NA	NA
hsa-miR-1973	URS00005030C1_9606	NA	NA	NA	NA
hsa-miR-544b	URS00000EE7F0_9606	NA	NA	NA	NA
hsa-miR-4722-3p	URS000041A3B2_9606	NA	NA	NA	NA
hsa-miR-3690	URS0000330DDA_9606	NA	NA	NA	NA
hsa-miR-8073	URS000075C665_9606	NA	NA	NA	NA
hsa-miR-4421	URS00002B0920_9606	NA	NA	NA	NA
hsa-miR-1248	URS0000057A7C_9606	NA	NA	NA	NA
hsa-miR-6841-3p	URS000075CB32_9606	NA	NA	NA	NA
hsa-miR-1915-5p	URS000075B84E_9606	NA	NA	NA	NA
hsa-miR-5091	URS00001D99D1_9606	NA	NA	NA	NA
hsa-miR-105-3p	URS00002872CB_9606	NA	NA	NA	NA
hsa-miR-6075	URS000075AA03_9606	NA	NA	NA	NA
hsa-miR-7112-5p	URS000075E3CE_9606	NA	NA	NA	NA
hsa-miR-137-5p	URS00004BCF82_9606	NA	NA	NA	NA
hsa-miR-5707	URS000075EFC2_9606	NA	NA	NA	NA
hsa-miR-4509	URS000075A776_9606	NA	NA	NA	NA
hsa-miR-190b-3p	URS0000D5564C_9606	NA	NA	NA	NA
hsa-miR-371b-5p	URS0000434271_9606	NA	NA	NA	NA
hsa-miR-373-5p	URS000075AC39_9606	NA	NA	NA	NA
hsa-miR-371a-5p	URS000025282C_9606	NA	NA	NA	NA
hsa-miR-4264	URS000075D2D0_9606	NA	NA	NA	NA
hsa-miR-3925-3p	URS0000295AD8_9606	NA	NA	NA	NA
hsa-miR-6814-3p	URS000075CD8B_9606	NA	NA	NA	NA
hsa-miR-4638-5p	URS000005CE28_9606	NA	NA	NA	NA
hsa-miR-3660	URS000058863C_9606	NA	NA	NA	NA
hsa-miR-6751-3p	URS000075EDBE_9606	NA	NA	NA	NA
hsa-miR-3920	URS000075CC01_9606	NA	NA	NA	NA
hsa-miR-6128	URS000075BB2F_9606	NA	NA	NA	NA

hsa-miR-378j	URS000075DCB5_9606	NA	NA	NA	NA
hsa-miR-6868-5p	URS000075CE71_9606	NA	NA	NA	NA
hsa-miR-4799-3p	URS00002B4F03_9606	NA	NA	NA	NA
hsa-miR-3176	URS00004AAAFF_9606	NA	NA	NA	NA
hsa-miR-6506-5p	URS000075D6B3_9606	NA	NA	NA	NA
hsa-miR-6860	URS000075BCE7_9606	NA	NA	NA	NA
hsa-miR-637	URS000075EF97_9606	NA	NA	NA	NA
hsa-miR-6861-5p	URS000075B829_9606	NA	NA	NA	NA
hsa-miR-20b-3p	URS00004FF449_9606	NA	NA	NA	NA
hsa-miR-6773-3p	URS000075E38F_9606	NA	NA	NA	NA
hsa-miR-367-5p	URS000075F0DB_9606	NA	NA	NA	NA
hsa-miR-302a-5p	URS000075C59E_9606	NA	NA	NA	NA
hsa-miR-600	URS000075B06B_9606	NA	NA	NA	NA
hsa-miR-412-3p	URS000075C49D_9606	NA	NA	NA	NA
hsa-miR-635	URS000075DC55_9606	NA	NA	NA	NA
hsa-miR-6774-5p	URS000075EDA5_9606	NA	NA	NA	NA
hsa-miR-302b-5p	URS000075BD14_9606	NA	NA	NA	NA
hsa-miR-302d-5p	URS000075A32E_9606	NA	NA	NA	NA
hsa-miR-3124-3p	URS000075A084_9606	NA	NA	NA	NA
hsa-miR-5681a	URS000075CE3C_9606	NA	NA	NA	NA
hsa-miR-4680-5p	URS0000217BB7_9606	NA	NA	NA	NA
hsa-miR-3185	URS0000152381_9606	NA	NA	NA	NA
hsa-miR-10522-5p	URS0000D53152_9606	NA	NA	NA	NA
hsa-miR-4699-5p	URS00004AB20A_9606	NA	NA	NA	NA
hsa-miR-1252-5p	URS000075C0BE_9606	NA	NA	NA	NA
hsa-miR-4493	URS000075E54E_9606	NA	NA	NA	NA
hsa-miR-7515	URS000075D66F_9606	NA	NA	NA	NA
hsa-miR-548ao-5p	URS000075B785_9606	NA	NA	NA	NA
hsa-miR-10393-5p	URS0000D50F76_9606	NA	NA	NA	NA
hsa-miR-3117-5p	URS00004977C5_9606	NA	NA	NA	NA
hsa-miR-591	URS000075DDAA_9606	NA	NA	NA	NA
hsa-miR-4725-5p	URS000036BA36_9606	NA	NA	NA	NA
hsa-miR-631	URS000075E148_9606	NA	NA	NA	NA
hsa-miR-4513	URS000075E3D4_9606	NA	NA	NA	NA
hsa-miR-617	URS000075B33B_9606	NA	NA	NA	NA
hsa-miR-6088	URS000075EC34_9606	NA	NA	NA	NA
hsa-miR-5186	URS000075B990_9606	NA	NA	NA	NA
hsa-miR-4468	URS000075D41E_9606	NA	NA	NA	NA
hsa-miR-3670	URS000075EB43_9606	NA	NA	NA	NA

hsa-miR-3160-3p	URS0000086029_9606	NA	NA	NA	NA
hsa-miR-4487	URS000075AB0E_9606	NA	NA	NA	NA
hsa-miR-4751	URS0000102473_9606	NA	NA	NA	NA
hsa-miR-4458	URS000032EF64_9606	NA	NA	NA	NA
hsa-miR-655-5p	URS000075AA28_9606	NA	NA	NA	NA
hsa-miR-924	URS000075B8FA_9606	NA	NA	NA	NA
hsa-miR-3171	URS000031F6C2_9606	NA	NA	NA	NA
hsa-miR-1468-3p	URS000075A234_9606	NA	NA	NA	NA
hsa-miR-4708-3p	URS00004F4FFB_9606	NA	NA	NA	NA
hsa-miR-8064	URS000075EDCC_9606	NA	NA	NA	NA
hsa-miR-298	URS000075E8F5_9606	NA	NA	NA	NA
hsa-miR-4771	URS000048E1E0_9606	NA	NA	NA	NA
hsa-miR-2467-3p	URS0000533099_9606	NA	NA	NA	NA
hsa-miR-6132	URS000075A7DE_9606	NA	NA	NA	NA
hsa-miR-3665	URS000075AFFF_9606	NA	NA	NA	NA
hsa-miR-6499-3p	URS000075C626_9606	NA	NA	NA	NA
hsa-miR-6076	URS00007599B2_9606	NA	NA	NA	NA
hsa-miR-4480	URS000075AC09_9606	NA	NA	NA	NA
hsa-miR-4755-3p	URS00003E226A_9606	NA	NA	NA	NA
hsa-miR-4313	URS000075A0F0_9606	NA	NA	NA	NA
hsa-miR-6743-3p	URS000075A77C_9606	NA	NA	NA	NA
hsa-miR-6811-3p	URS000075DB58_9606	NA	NA	NA	NA
hsa-miR-5581-5p	URS000075AD14_9606	NA	NA	NA	NA
hsa-miR-381-5p	URS00004A35E2_9606	NA	NA	NA	NA
hsa-miR-4706	URS00000C403E_9606	NA	NA	NA	NA
hsa-miR-4746-3p	URS000040D421_9606	NA	NA	NA	NA
hsa-miR-6853-5p	URS000075C2F0_9606	NA	NA	NA	NA
hsa-miR-548v	URS000045E0EC_9606	NA	NA	NA	NA
hsa-miR-7106-3p	URS000075C7CF_9606	NA	NA	NA	NA
hsa-miR-4663	URS000055E478_9606	NA	NA	NA	NA
hsa-miR-133a-5p	URS0000383E7F_9606	NA	NA	NA	NA
hsa-miR-1537-5p	URS0000759C89_9606	NA	NA	NA	NA
hsa-miR-4718	URS00000E8004_9606	NA	NA	NA	NA
hsa-miR-626	URS000075E887_9606	NA	NA	NA	NA
hsa-miR-4261	URS000075C945_9606	NA	NA	NA	NA
hsa-miR-5093	URS000075BA89_9606	NA	NA	NA	NA
hsa-miR-613	URS000075B7E4_9606	NA	NA	NA	NA
hsa-miR-492	URS000032599B_9606	NA	NA	NA	NA
hsa-miR-4515	URS00005D35D9_9606	NA	NA	NA	NA

hsa-miR-4434	URS000075CBBF_9606	NA	NA	NA	NA
hsa-miR-5703	URS00005DEC41_9606	NA	NA	NA	NA
hsa-miR-6081	URS0000759AA7_9606	NA	NA	NA	NA
hsa-miR-4633-3p	URS0000112018_9606	NA	NA	NA	NA
hsa-miR-6500-5p	URS000075DAB5_9606	NA	NA	NA	NA
hsa-miR-7154-3p	URS000075A93E_9606	NA	NA	NA	NA
hsa-miR-650	URS000075A00C_9606	NA	NA	NA	NA
hsa-miR-6797-5p	URS000075DF47_9606	NA	NA	NA	NA
hsa-miR-1249-5p	URS0000782078_9606	NA	NA	NA	NA
hsa-miR-5191	URS000075CB1C_9606	NA	NA	NA	NA
hsa-miR-3167	URS000075AD9F_9606	NA	NA	NA	NA
hsa-miR-593-5p	URS0000759B98_9606	NA	NA	NA	NA
hsa-miR-564	URS000075ED17_9606	NA	NA	NA	NA
hsa-miR-4763-3p	URS00004A40D8_9606	NA	NA	NA	NA
hsa-miR-4736	URS0000077E9F_9606	NA	NA	NA	NA
hsa-miR-449b-5p	URS00003758F0_9606	NA	NA	NA	NA
hsa-miR-1256	URS0000098B3B_9606	NA	NA	NA	NA
hsa-miR-4529-5p	URS00005D0CA3_9606	NA	NA	NA	NA
hsa-miR-6775-3p	URS000075A8B6_9606	NA	NA	NA	NA
hsa-miR-6735-3p	URS000075AB2A_9606	NA	NA	NA	NA
hsa-miR-6722-5p	URS000075C73A_9606	NA	NA	NA	NA
hsa-miR-663a	URS00004929F1_9606	NA	NA	NA	NA
hsa-miR-6822-3p	URS000075E00B_9606	NA	NA	NA	NA
hsa-miR-604	URS000075DB77_9606	NA	NA	NA	NA
hsa-miR-4505	URS000075EBEE_9606	NA	NA	NA	NA
hsa-miR-7974	URS000075E96D_9606	NA	NA	NA	NA
hsa-miR-5196-5p	URS0000214763_9606	NA	NA	NA	NA
hsa-miR-3649	URS000075BD6C_9606	NA	NA	NA	NA
hsa-miR-6878-5p	URS000075BBD5_9606	NA	NA	NA	NA
hsa-miR-6883-5p	URS000075B504_9606	NA	NA	NA	NA
hsa-miR-638	URS000075DB2F_9606	NA	NA	NA	NA
hsa-miR-3945	URS000075B173_9606	NA	NA	NA	NA
hsa-miR-4253	URS000075C0DF_9606	NA	NA	NA	NA
hsa-miR-3144-5p	URS00002A79D1_9606	NA	NA	NA	NA
hsa-miR-4652-5p	URS00005186AB_9606	NA	NA	NA	NA
hsa-miR-4697-5p	URS0000592E67_9606	NA	NA	NA	NA
hsa-miR-6885-5p	URS000075D552_9606	NA	NA	NA	NA
hsa-miR-6835-5p	URS000075A5BD_9606	NA	NA	NA	NA
hsa-miR-608	URS000075EBEF_9606	NA	NA	NA	NA

hsa-miR-6747-5p	URS000075A10C_9606	NA	NA	NA	NA
hsa-miR-555	URS000075B6A9_9606	NA	NA	NA	NA
hsa-miR-6756-5p	URS000075A4DE_9606	NA	NA	NA	NA
hsa-miR-4455	URS00005F9A1A_9606	NA	NA	NA	NA
hsa-miR-5681b	URS000075E035_9606	NA	NA	NA	NA
hsa-miR-3085-5p	URS0000D56B0E_9606	NA	NA	NA	NA
hsa-miR-3907	URS000075D93D_9606	NA	NA	NA	NA
hsa-miR-3165	URS0000237E04_9606	NA	NA	NA	NA
hsa-miR-6769a-5p	URS000075A69A_9606	NA	NA	NA	NA
hsa-miR-323a-5p	URS000018E72A_9606	NA	NA	NA	NA
hsa-miR-3650	URS000075C93C_9606	NA	NA	NA	NA
hsa-miR-4694-5p	URS00000E780E_9606	NA	NA	NA	NA
hsa-miR-563	URS000075C22C_9606	NA	NA	NA	NA
hsa-miR-656-5p	URS000075DD8D_9606	NA	NA	NA	NA
hsa-miR-323b-5p	URS000075D04C_9606	NA	NA	NA	NA
hsa-miR-494-5p	URS000075CDF9_9606	NA	NA	NA	NA
hsa-miR-410-5p	URS00002233F4_9606	NA	NA	NA	NA
hsa-miR-6832-5p	URS0000759BAE_9606	NA	NA	NA	NA
hsa-miR-567	URS000075CED1_9606	NA	NA	NA	NA
hsa-miR-630	URS000075AFB4_9606	NA	NA	NA	NA
hsa-miR-1257	URS000075B4B8_9606	NA	NA	NA	NA
hsa-miR-3688-5p	URS000025CFB9_9606	NA	NA	NA	NA
hsa-miR-891a-3p	URS000075A749_9606	NA	NA	NA	NA
hsa-miR-6778-5p	URS000075B2C3_9606	NA	NA	NA	NA
hsa-miR-1233-5p	URS000075C9B8_9606	NA	NA	NA	NA
hsa-miR-6742-5p	URS000075B7CE_9606	NA	NA	NA	NA
hsa-miR-203a-5p	URS0000770B9E_9606	NA	NA	NA	NA
hsa-miR-644a	URS000075C3AA_9606	NA	NA	NA	NA
hsa-miR-569	URS000075E39A_9606	NA	NA	NA	NA
hsa-miR-19b-2-5p	URS000075D58E_9606	NA	NA	NA	NA
hsa-miR-19a-5p	URS00001754CF_9606	NA	NA	NA	NA
hsa-miR-3143	URS0000610EFA_9606	NA	NA	NA	NA
hsa-miR-374c-5p	URS0000517429_9606	NA	NA	NA	NA
hsa-miR-4666a-5p	URS00001D54AD_9606	NA	NA	NA	NA
hsa-miR-5591-3p	URS000075D351_9606	NA	NA	NA	NA
hsa-miR-4693-5p	URS0000142768_9606	NA	NA	NA	NA
hsa-miR-4524b-5p	URS000075ADAD_9606	NA	NA	NA	NA
hsa-miR-4423-3p	URS00001481F9_9606	NA	NA	NA	NA
hsa-miR-1185-2-3p	URS0000484751_9606	NA	NA	NA	NA

hsa-miR-4633-5p	URS0000085250_9606	NA	NA	NA	NA
hsa-miR-3924	URS000075D5EB_9606	NA	NA	NA	NA
hsa-miR-3671	URS000075E9F9_9606	NA	NA	NA	NA
hsa-miR-539-3p	URS000039607D_9606	NA	NA	NA	NA
hsa-miR-5007-3p	URS000075A772_9606	NA	NA	NA	NA
hsa-miR-6516-3p	URS000075BD04_9606	NA	NA	NA	NA
hsa-miR-5195-3p	URS00007E42F5_9606	NA	NA	NA	NA
hsa-miR-623	URS000075DCB1_9606	NA	NA	NA	NA
hsa-miR-6881-3p	URS000075D12C_9606	NA	NA	NA	NA
hsa-miR-639	URS000075B8B8_9606	NA	NA	NA	NA
hsa-miR-4790-5p	URS00004B2CF8_9606	NA	NA	NA	NA
hsa-miR-3181	URS00007E410F_9606	NA	NA	NA	NA
hsa-miR-517c-3p	URS00003FBECA_9606	NA	NA	NA	NA
hsa-miR-4256	URS000075D5EF_9606	NA	NA	NA	NA
hsa-miR-4727-5p	URS00005C8887_9606	NA	NA	NA	NA
hsa-miR-6828-3p	URS000075A876_9606	NA	NA	NA	NA
hsa-miR-516b-5p	URS000005E7DD_9606	NA	NA	NA	NA
hsa-miR-3686	URS000075B3BC_9606	NA	NA	NA	NA
hsa-miR-7843-3p	URS000075D5E9_9606	NA	NA	NA	NA
hsa-miR-3616-5p	URS000075EF21_9606	NA	NA	NA	NA
hsa-miR-3672	URS000075CA6F_9606	NA	NA	NA	NA
hsa-miR-5708	URS000075CEDD_9606	NA	NA	NA	NA
hsa-miR-640	URS000075D1E0_9606	NA	NA	NA	NA
hsa-miR-6811-5p	URS000075CB61_9606	NA	NA	NA	NA
hsa-miR-6836-3p	URS000075D7D0_9606	NA	NA	NA	NA
hsa-miR-4737	URS000057DFC6_9606	NA	NA	NA	NA
hsa-miR-4531	URS0000034450_9606	NA	NA	NA	NA
hsa-miR-620	URS000075B716_9606	NA	NA	NA	NA
hsa-miR-6874-5p	URS000075C2DE_9606	NA	NA	NA	NA
hsa-miR-4642	URS0000477B7D_9606	NA	NA	NA	NA
hsa-miR-548s	URS000047A67D_9606	NA	NA	NA	NA
hsa-miR-6812-5p	URS000075F0BD_9606	NA	NA	NA	NA
hsa-miR-4701-3p	URS000006983A_9606	NA	NA	NA	NA
hsa-miR-5587-5p	URS000075B8EE_9606	NA	NA	NA	NA
hsa-miR-6843-3p	URS000075D3D1_9606	NA	NA	NA	NA
hsa-miR-568	URS000075CC27_9606	NA	NA	NA	NA
hsa-miR-297	URS000075D760_9606	NA	NA	NA	NA
hsa-miR-3912-5p	URS000075C7F0_9606	NA	NA	NA	NA
hsa-miR-8070	URS000075E1F5_9606	NA	NA	NA	NA

hsa-miR-550b-2-5p	URS000009C1E9_9606	NA	NA	NA	NA
hsa-miR-4477b	URS000075BEA7_9606	NA	NA	NA	NA
hsa-miR-4263	URS000075E59C_9606	NA	NA	NA	NA
hsa-miR-4768-5p	URS0000274F93_9606	NA	NA	NA	NA
hsa-miR-6875-3p	URS000075D4A7_9606	NA	NA	NA	NA
hsa-miR-4774-3p	URS0000495FF7_9606	NA	NA	NA	NA
hsa-miR-3152-5p	URS00000962CC_9606	NA	NA	NA	NA
hsa-miR-3674	URS000075D2BF_9606	NA	NA	NA	NA
hsa-miR-4646-3p	URS00005BBF99_9606	NA	NA	NA	NA
hsa-miR-6739-3p	URS000075C88F_9606	NA	NA	NA	NA
hsa-miR-4446-5p	URS00001BDD9A_9606	NA	NA	NA	NA
hsa-miR-2113	URS000075C7E0_9606	NA	NA	NA	NA
hsa-miR-548bb-3p	URS00007E4A05_9606	NA	NA	NA	NA
hsa-miR-548ah-3p	URS0000462CE4_9606	NA	NA	NA	NA
hsa-miR-6771-3p	URS000075EE65_9606	NA	NA	NA	NA
hsa-miR-583	URS000075B05D_9606	NA	NA	NA	NA
hsa-miR-518b	URS00003676C9_9606	NA	NA	NA	NA
hsa-miR-518d-3p	URS00001B6361_9606	NA	NA	NA	NA
hsa-miR-518c-3p	URS0000759F7E_9606	NA	NA	NA	NA
hsa-miR-6768-3p	URS000075A069_9606	NA	NA	NA	NA
hsa-miR-6507-3p	URS000075B7B9_9606	NA	NA	NA	NA
hsa-miR-3609	URS000006F90B_9606	NA	NA	NA	NA
hsa-miR-519d-3p	URS0000298BA3_9606	NA	NA	NA	NA
hsa-miR-5692a	URS000075A42B_9606	NA	NA	NA	NA
hsa-miR-1252-3p	URS000075D86C_9606	NA	NA	NA	NA
hsa-miR-4694-3p	URS0000156205_9606	NA	NA	NA	NA
hsa-miR-196a-1-3p	URS0000D5297E_9606	NA	NA	NA	NA
hsa-miR-6776-3p	URS000075CDD0_9606	NA	NA	NA	NA
hsa-miR-12118	URS0000D54202_9606	NA	NA	NA	NA
hsa-miR-4475	URS0000759E95_9606	NA	NA	NA	NA
hsa-miR-5197-5p	URS000075A40B_9606	NA	NA	NA	NA
hsa-miR-8066	URS000075E442_9606	NA	NA	NA	NA
hsa-miR-5571-5p	URS000029EAB2_9606	NA	NA	NA	NA
hsa-miR-6768-5p	URS000075B489_9606	NA	NA	NA	NA
hsa-miR-4789-3p	URS00002D6EA8_9606	NA	NA	NA	NA
hsa-miR-4700-3p	URS00003840E5_9606	NA	NA	NA	NA
hsa-miR-4438	URS0000759B8B_9606	NA	NA	NA	NA
hsa-miR-5580-3p	URS000075ECCB_9606	NA	NA	NA	NA
hsa-miR-1911-3p	URS0000759AE6_9606	NA	NA	NA	NA

hsa-miR-7153-3p	URS000075E990_9606	NA	NA	NA	NA
hsa-miR-1295b-5p	URS000075EE23_9606	NA	NA	NA	NA
hsa-miR-4445-3p	URS000075C856_9606	NA	NA	NA	NA
hsa-miR-512-5p	URS0000062B37_9606	NA	NA	NA	NA
hsa-miR-4418	URS000075ADC1_9606	NA	NA	NA	NA
hsa-miR-6807-3p	URS000075EEBD_9606	NA	NA	NA	NA
hsa-miR-1183	URS000075A336_9606	NA	NA	NA	NA
hsa-miR-4318	URS000075AC68_9606	NA	NA	NA	NA
hsa-miR-374c-3p	URS0000140295_9606	NA	NA	NA	NA
hsa-miR-3123	URS00000939AF_9606	NA	NA	NA	NA
hsa-miR-6873-5p	URS000075A286_9606	NA	NA	NA	NA
hsa-miR-5586-3p	URS000075D7E6_9606	NA	NA	NA	NA
hsa-miR-5694	URS000075AB57_9606	NA	NA	NA	NA
hsa-miR-6871-3p	URS000075BEDF_9606	NA	NA	NA	NA
hsa-miR-6165	URS0000298172_9606	NA	NA	NA	NA
hsa-miR-4274	URS000075E6E7_9606	NA	NA	NA	NA
hsa-miR-4519	URS000075DD24_9606	NA	NA	NA	NA
hsa-miR-449b-3p	URS0000490063_9606	NA	NA	NA	NA
hsa-miR-6858-3p	URS000075D01E_9606	NA	NA	NA	NA
hsa-miR-4323	URS000075EB70_9606	NA	NA	NA	NA
hsa-miR-4687-5p	URS00003B6938_9606	NA	NA	NA	NA
hsa-miR-6746-3p	URS000075DC00_9606	NA	NA	NA	NA
hsa-miR-6877-3p	URS000075CBC4_9606	NA	NA	NA	NA
hsa-miR-4293	URS000075DAF5_9606	NA	NA	NA	NA
hsa-miR-7846-3p	URS000075B936_9606	NA	NA	NA	NA
hsa-miR-6876-5p	URS0000759DEA_9606	NA	NA	NA	NA
hsa-miR-4476	URS000075B0F6_9606	NA	NA	NA	NA
hsa-miR-8083	URS000075BA5F_9606	NA	NA	NA	NA
hsa-miR-4695-5p	URS0000293918_9606	NA	NA	NA	NA
hsa-miR-4661-3p	URS00001D68C9_9606	NA	NA	NA	NA
hsa-miR-10524-5p	URS0000D50577_9606	NA	NA	NA	NA
hsa-miR-1265	URS000075B9B8_9606	NA	NA	NA	NA
hsa-miR-6808-5p	URS000075A409_9606	NA	NA	NA	NA
hsa-miR-6893-5p	URS000075C9E1_9606	NA	NA	NA	NA
hsa-miR-2682-5p	URS0000366551_9606	NA	NA	NA	NA
hsa-miR-3186-5p	URS000075C3AF_9606	NA	NA	NA	NA
hsa-miR-6760-5p	URS000075BFA0_9606	NA	NA	NA	NA
hsa-miR-4756-5p	URS00005A37D5_9606	NA	NA	NA	NA
hsa-miR-1321	URS000075A206_9606	NA	NA	NA	NA

hsa-miR-6895-5p	URS000075CE58_9606	NA	NA	NA	NA
hsa-miR-7160-3p	URS000075CE84_9606	NA	NA	NA	NA
hsa-miR-4512	URS0000247AC6_9606	NA	NA	NA	NA
hsa-miR-6794-5p	URS000075A643_9606	NA	NA	NA	NA
hsa-miR-1178-5p	URS00008E3963_9606	NA	NA	NA	NA
hsa-miR-3927-3p	URS000075EF94_9606	NA	NA	NA	NA
hsa-miR-370-5p	URS000075E3E2_9606	NA	NA	NA	NA
hsa-miR-802	URS000075A099_9606	NA	NA	NA	NA
hsa-miR-8079	URS000075BA0A_9606	NA	NA	NA	NA
hsa-miR-8062	URS000075BBC9_9606	NA	NA	NA	NA
hsa-miR-3666	URS000075B762_9606	NA	NA	NA	NA
hsa-miR-4295	URS000075BA14_9606	NA	NA	NA	NA
hsa-miR-33b-3p	URS00001270D3_9606	NA	NA	NA	NA
hsa-miR-3677-5p	URS0000307592_9606	NA	NA	NA	NA
hsa-miR-4255	URS000075A8EB_9606	NA	NA	NA	NA
hsa-miR-5000-5p	URS000075A2CA_9606	NA	NA	NA	NA
hsa-miR-4259	URS00007E453F_9606	NA	NA	NA	NA
hsa-miR-4666a-3p	URS00004489C5_9606	NA	NA	NA	NA
hsa-miR-10397-3p	URS0000D507F2_9606	NA	NA	NA	NA
hsa-miR-3651	URS0000299AD8_9606	NA	NA	NA	NA
hsa-miR-2115-3p	URS000075A638_9606	NA	NA	NA	NA
hsa-miR-217-3p	URS000075DC53_9606	NA	NA	NA	NA
hsa-miR-3675-3p	URS000075E61A_9606	NA	NA	NA	NA
hsa-miR-6504-3p	URS000075C041_9606	NA	NA	NA	NA
hsa-miR-4272	URS000075E244_9606	NA	NA	NA	NA
hsa-miR-6830-5p	URS000075BBFB_9606	NA	NA	NA	NA
hsa-miR-4275	URS000075B95D_9606	NA	NA	NA	NA
hsa-miR-6767-3p	URS000075ED01_9606	NA	NA	NA	NA
hsa-miR-6890-3p	URS000075BFFE_9606	NA	NA	NA	NA
hsa-miR-1324	URS000075F046_9606	NA	NA	NA	NA
hsa-miR-4494	URS000075BBE0_9606	NA	NA	NA	NA
hsa-miR-4756-3p	URS000038D12F_9606	NA	NA	NA	NA
hsa-miR-6501-3p	URS000075EBDF_9606	NA	NA	NA	NA
hsa-miR-4257	URS000075D6B0_9606	NA	NA	NA	NA
hsa-miR-4691-3p	URS000012F9EC_9606	NA	NA	NA	NA
hsa-miR-4768-3p	URS00002B3F70_9606	NA	NA	NA	NA
hsa-miR-3155b	URS0000759908_9606	NA	NA	NA	NA
hsa-miR-5190	URS000075EB25_9606	NA	NA	NA	NA
hsa-miR-4302	URS000075E65D_9606	NA	NA	NA	NA

hsa-miR-4328	URS000030B5E2_9606	NA	NA	NA	NA
hsa-miR-8060	URS000075BDAC_9606	NA	NA	NA	NA
hsa-miR-10226	URS0000D512E7_9606	NA	NA	NA	NA
hsa-miR-9902	URS0000D549DA_9606	NA	NA	NA	NA
hsa-miR-4530	URS000075E84B_9606	NA	NA	NA	NA
hsa-miR-6884-3p	URS000075E1B2_9606	NA	NA	NA	NA
hsa-miR-6872-3p	URS000075F01C_9606	NA	NA	NA	NA
hsa-miR-4258	URS000075AD5E_9606	NA	NA	NA	NA
hsa-miR-1247-3p	URS000032835F_9606	NA	NA	NA	NA
hsa-miR-4292	URS000075A553_9606	NA	NA	NA	NA
hsa-miR-6886-5p	URS000075EF90_9606	NA	NA	NA	NA
hsa-miR-1203	URS000075A53F_9606	NA	NA	NA	NA
hsa-miR-6850-3p	URS000075B553_9606	NA	NA	NA	NA
hsa-miR-6891-3p	URS000075A640_9606	NA	NA	NA	NA
hsa-miR-4723-3p	URS000051E359_9606	NA	NA	NA	NA
hsa-miR-6893-3p	URS000075AE91_9606	NA	NA	NA	NA
hsa-miR-939-3p	URS00003AD2AA_9606	NA	NA	NA	NA
hsa-miR-1914-5p	URS000075C11C_9606	NA	NA	NA	NA
hsa-miR-1266-3p	URS0000759C7A_9606	NA	NA	NA	NA
hsa-miR-6078	URS000075CCC7_9606	NA	NA	NA	NA
hsa-miR-6812-3p	URS000075C15B_9606	NA	NA	NA	NA
hsa-miR-4315	URS000075EF88_9606	NA	NA	NA	NA
hsa-miR-4304	URS000075E8E8_9606	NA	NA	NA	NA
hsa-miR-10392-3p	URS0000D53E45_9606	NA	NA	NA	NA
hsa-miR-6746-5p	URS000075AF8F_9606	NA	NA	NA	NA
hsa-miR-6070	URS000075EFDD_9606	NA	NA	NA	NA
hsa-miR-1181	URS000075CF96_9606	NA	NA	NA	NA
hsa-miR-8067	URS000075BF41_9606	NA	NA	NA	NA
hsa-miR-4305	URS000075AA82_9606	NA	NA	NA	NA
hsa-miR-325	URS000075C2FC_9606	NA	NA	NA	NA
hsa-miR-372-5p	URS000075B4E5_9606	NA	NA	NA	NA
hsa-miR-6862-3p	URS0000759D25_9606	NA	NA	NA	NA
hsa-miR-4330	URS000075AEBE_9606	NA	NA	NA	NA
hsa-miR-519d-5p	URS000075AFE4_9606	NA	NA	NA	NA
hsa-miR-7113-3p	URS000075D451_9606	NA	NA	NA	NA
hsa-miR-8088	URS000075D4E7_9606	NA	NA	NA	NA
hsa-miR-520f-5p	URS000075BA1C_9606	NA	NA	NA	NA
hsa-miR-520b-5p	URS0000D5654E_9606	NA	NA	NA	NA
hsa-miR-517-5p	URS0000227241_9606	NA	NA	NA	NA

hsa-miR-6845-3p	URS000075E6C8_9606	NA	NA	NA	NA
hsa-miR-6730-3p	URS000075E787_9606	NA	NA	NA	NA
hsa-miR-4750-3p	URS000075EC59_9606	NA	NA	NA	NA
hsa-miR-4251	URS000075DEB4_9606	NA	NA	NA	NA
hsa-miR-4329	URS000075B49A_9606	NA	NA	NA	NA
hsa-miR-1199-5p	URS000075DBCC_9606	NA	NA	NA	NA
hsa-miR-3151-3p	URS000075B369_9606	NA	NA	NA	NA
hsa-miR-3158-5p	URS0000221D0E_9606	NA	NA	NA	NA
hsa-miR-1184	URS000075B5BA_9606	NA	NA	NA	NA
hsa-miR-6068	URS000075E142_9606	NA	NA	NA	NA
hsa-miR-3692-5p	URS000075A198_9606	NA	NA	NA	NA
hsa-miR-875-3p	URS000075B7A1_9606	NA	NA	NA	NA
hsa-miR-4638-3p	URS0000403BF8_9606	NA	NA	NA	NA
hsa-miR-4720-5p	URS00000DE8FA_9606	NA	NA	NA	NA
hsa-miR-3187-5p	URS0000486E0B_9606	NA	NA	NA	NA
hsa-miR-8089	URS000075D801_9606	NA	NA	NA	NA
hsa-miR-4456	URS000075DA57_9606	NA	NA	NA	NA
hsa-miR-3120-5p	URS0000509DB8_9606	NA	NA	NA	NA
hsa-miR-6529-3p	URS0000D54054_9606	NA	NA	NA	NA
hsa-miR-134-3p	URS000075CBC9_9606	NA	NA	NA	NA
hsa-miR-6790-3p	URS000075CD03_9606	NA	NA	NA	NA
hsa-miR-6738-5p	URS000075A6C6_9606	NA	NA	NA	NA
hsa-miR-6804-3p	URS000075C72B_9606	NA	NA	NA	NA
hsa-miR-3166	URS000075B8E5_9606	NA	NA	NA	NA
hsa-miR-6836-5p	URS000075CC00_9606	NA	NA	NA	NA
hsa-miR-1909-3p	URS000009A9A2_9606	NA	NA	NA	NA
hsa-miR-6869-3p	URS000075E76C_9606	NA	NA	NA	NA
hsa-miR-2682-3p	URS000075AE09_9606	NA	NA	NA	NA
hsa-miR-4763-5p	URS000075B1DD_9606	NA	NA	NA	NA
hsa-miR-6720-3p	URS000075D814_9606	NA	NA	NA	NA
hsa-miR-3621	URS00001CBC9F_9606	NA	NA	NA	NA
hsa-miR-1301-5p	URS000077643D_9606	NA	NA	NA	NA
hsa-miR-9851-5p	URS0000D50487_9606	NA	NA	NA	NA
hsa-miR-1251-3p	URS0000113463_9606	NA	NA	NA	NA
hsa-miR-196a-3p	URS00000E4877_9606	NA	NA	NA	NA
hsa-miR-6789-3p	URS000075E505_9606	NA	NA	NA	NA
hsa-miR-4634	URS0000512F70_9606	NA	NA	NA	NA
hsa-miR-10400-5p	URS0000D566B9_9606	NA	NA	NA	NA
hsa-miR-10396b-5p	URS0000D51A84_9606	NA	NA	NA	NA

hsa-miR-8052	URS000075E2FE_9606	NA	NA	NA	NA
hsa-miR-4741	URS0000547F6A_9606	NA	NA	NA	NA
hsa-miR-6762-5p	URS000075C247_9606	NA	NA	NA	NA
hsa-miR-4651	URS00005F9738_9606	NA	NA	NA	NA
hsa-miR-6766-5p	URS000075E3FA_9606	NA	NA	NA	NA
hsa-miR-12130	URS0000D519C7_9606	NA	NA	NA	NA
hsa-miR-9901	URS0000D500A8_9606	NA	NA	NA	NA
hsa-miR-8071	URS000075C590_9606	NA	NA	NA	NA
hsa-miR-4433a-5p	URS000075A620_9606	NA	NA	NA	NA
hsa-miR-4449	URS00004DE2FC_9606	NA	NA	NA	NA
hsa-miR-8056	URS000075C623_9606	NA	NA	NA	NA
hsa-miR-4711-3p	URS000007BE64_9606	NA	NA	NA	NA
hsa-miR-633	URS000075EE25_9606	NA	NA	NA	NA
hsa-miR-524-5p	URS00003E236A_9606	NA	NA	NA	NA
hsa-miR-520d-5p	URS00002A037E_9606	NA	NA	NA	NA
hsa-miR-6798-3p	URS000075C554_9606	NA	NA	NA	NA
hsa-miR-7848-3p	URS000075EF24_9606	NA	NA	NA	NA
hsa-miR-3682-5p	URS000075B2B0_9606	NA	NA	NA	NA
hsa-miR-4266	URS000075B9A3_9606	NA	NA	NA	NA
hsa-miR-4278	URS000075D2F2_9606	NA	NA	NA	NA
hsa-miR-921	URS0000759CD8_9606	NA	NA	NA	NA
hsa-miR-4473	URS000075A17D_9606	NA	NA	NA	NA
hsa-miR-8074	URS000075DED4_9606	NA	NA	NA	NA
hsa-miR-4477a	URS000075ABFD_9606	NA	NA	NA	NA
hsa-miR-6715b-3p	URS000075B498_9606	NA	NA	NA	NA
hsa-miR-520e-5p	URS0000D4F992_9606	NA	NA	NA	NA
hsa-miR-4465	URS000075D297_9606	NA	NA	NA	NA
hsa-miR-6794-3p	URS000075AB87_9606	NA	NA	NA	NA
hsa-miR-4276	URS000075E8E2_9606	NA	NA	NA	NA
hsa-miR-452-3p	URS0000101787_9606	NA	NA	NA	NA
hsa-miR-1912-5p	URS0000D51A0B_9606	NA	NA	NA	NA
hsa-miR-520a-5p	URS0000534239_9606	NA	NA	NA	NA
hsa-miR-525-5p	URS0000459998_9606	NA	NA	NA	NA
hsa-miR-3156-3p	URS000075D48C_9606	NA	NA	NA	NA
hsa-miR-6763-3p	URS000075B8D7_9606	NA	NA	NA	NA
hsa-miR-6826-3p	URS000075D5F4_9606	NA	NA	NA	NA
hsa-miR-155-3p	URS000034309A_9606	NA	NA	NA	NA
hsa-miR-6792-3p	URS000075E024_9606	NA	NA	NA	NA
hsa-miR-6749-3p	URS000075B263_9606	NA	NA	NA	NA

hsa-miR-6780a-3p	URS000075A7D3_9606	NA	NA	NA	NA
hsa-miR-1200	URS000075DC9F_9606	NA	NA	NA	NA
hsa-miR-4444	URS00002A82B3_9606	NA	NA	NA	NA
hsa-miR-4665-3p	URS00000BD6DC_9606	NA	NA	NA	NA
hsa-miR-1469	URS0000539433_9606	NA	NA	NA	NA
hsa-miR-6771-5p	URS000075EF6D_9606	NA	NA	NA	NA
hsa-miR-6727-5p	URS000075A9AA_9606	NA	NA	NA	NA
hsa-miR-3677-3p	URS0000171020_9606	NA	NA	NA	NA
hsa-miR-518f-5p	URS00005CF1BF_9606	NA	NA	NA	NA
hsa-miR-526a-5p	URS0000087207_9606	NA	NA	NA	NA
hsa-miR-6867-3p	URS000075AB8A_9606	NA	NA	NA	NA
hsa-miR-4279	URS000075D139_9606	NA	NA	NA	NA
hsa-miR-3191-5p	URS0000782790_9606	NA	NA	NA	NA
hsa-miR-3192-3p	URS000075D74E_9606	NA	NA	NA	NA
hsa-miR-4314	URS000075BC08_9606	NA	NA	NA	NA
hsa-miR-526b-5p	URS00004C21E4_9606	NA	NA	NA	NA
hsa-miR-573	URS000058BB84_9606	NA	NA	NA	NA
hsa-miR-518a-5p	URS0000068BEA_9606	NA	NA	NA	NA
hsa-miR-3678-3p	URS000075BC79_9606	NA	NA	NA	NA
hsa-miR-12121	URS0000D52CE1_9606	NA	NA	NA	NA
hsa-miR-3972	URS000075E1EC_9606	NA	NA	NA	NA
hsa-miR-6514-3p	URS000075C48C_9606	NA	NA	NA	NA
hsa-miR-1269b	URS000015CE89_9606	NA	NA	NA	NA
hsa-miR-1269a	URS0000026A1D_9606	NA	NA	NA	NA
hsa-miR-8058	URS000075E3DD_9606	NA	NA	NA	NA
hsa-miR-4309	URS000075CF21_9606	NA	NA	NA	NA
hsa-miR-432-3p	URS0000105D19_9606	NA	NA	NA	NA
hsa-miR-7150	URS000075DD8E_9606	NA	NA	NA	NA
hsa-miR-6878-3p	URS000075D163_9606	NA	NA	NA	NA
hsa-miR-4730	URS00002E4C79_9606	NA	NA	NA	NA
hsa-miR-6770-3p	URS000075D6C4_9606	NA	NA	NA	NA
hsa-miR-4298	URS00000726BD_9606	NA	NA	NA	NA
hsa-miR-3689a-3p	URS000075BBD1_9606	NA	NA	NA	NA
hsa-miR-3689b-3p	URS000075BB55_9606	NA	NA	NA	NA
hsa-miR-10400-3p	URS0000D520C1_9606	NA	NA	NA	NA
hsa-miR-4674	URS000038E667_9606	NA	NA	NA	NA
hsa-miR-7109-5p	URS000075EDFB_9606	NA	NA	NA	NA
hsa-miR-6803-5p	URS000075A789_9606	NA	NA	NA	NA
hsa-miR-3170	URS000042935A_9606	NA	NA	NA	NA

hsa-miR-2054	URS000075EFDE_9606	NA	NA	NA	NA
hsa-miR-4322	URS000075EA05_9606	NA	NA	NA	NA
hsa-miR-4265	URS000075C018_9606	NA	NA	NA	NA
hsa-miR-8061	URS000075E23B_9606	NA	NA	NA	NA
hsa-miR-6083	URS000075D3D4_9606	NA	NA	NA	NA
hsa-miR-3180-5p	URS00004EE409_9606	NA	NA	NA	NA
hsa-miR-718	URS000075B1FD_9606	NA	NA	NA	NA
hsa-miR-6738-3p	URS000075A1B6_9606	NA	NA	NA	NA
hsa-miR-8081	URS000075B5D0_9606	NA	NA	NA	NA
hsa-miR-4776-3p	URS0000396F65_9606	NA	NA	NA	NA
hsa-miR-5004-3p	URS000075DCE2_9606	NA	NA	NA	NA
hsa-miR-12133	URS0000D5391E_9606	NA	NA	NA	NA
hsa-miR-4260	URS000075B003_9606	NA	NA	NA	NA
hsa-miR-659-3p	URS000075C04A_9606	NA	NA	NA	NA
hsa-miR-8055	URS000075DB01_9606	NA	NA	NA	NA
hsa-miR-548ad-3p	URS00000319E1_9606	NA	NA	NA	NA
hsa-miR-4668-3p	URS0000065FDF_9606	NA	NA	NA	NA
hsa-miR-3662	URS00003808EC_9606	NA	NA	NA	NA
hsa-miR-4311	URS000075E69C_9606	NA	NA	NA	NA
hsa-miR-518a-3p	URS0000024ACC_9606	NA	NA	NA	NA
hsa-miR-526a-3p	URS0000D54612_9606	NA	NA	NA	NA
hsa-miR-518f-3p	URS000075E9BD_9606	NA	NA	NA	NA
hsa-miR-8054	URS000075F0EE_9606	NA	NA	NA	NA
hsa-miR-526b-3p	URS000038B25B_9606	NA	NA	NA	NA
hsa-miR-551b-5p	URS000025A6B4_9606	NA	NA	NA	NA
hsa-miR-614	URS000075E5BD_9606	NA	NA	NA	NA
hsa-miR-523-3p	URS00001383CF_9606	NA	NA	NA	NA
hsa-miR-8087	URS000075A8CE_9606	NA	NA	NA	NA
hsa-miR-4426	URS00002731AC_9606	NA	NA	NA	NA
hsa-miR-4647	URS000024CEE0_9606	NA	NA	NA	NA
hsa-miR-6796-3p	URS000075E480_9606	NA	NA	NA	NA
hsa-miR-8080	URS000075AAE2_9606	NA	NA	NA	NA
hsa-miR-6816-3p	URS000075A9E7_9606	NA	NA	NA	NA
hsa-miR-3714	URS000075CF0B_9606	NA	NA	NA	NA
hsa-miR-525-3p	URS000075C235_9606	NA	NA	NA	NA
hsa-miR-524-3p	URS00003BD367_9606	NA	NA	NA	NA
hsa-miR-373-3p	URS0000508C13_9606	NA	NA	NA	NA
hsa-miR-12117	URS0000D51AF9_9606	NA	NA	NA	NA
hsa-miR-595	URS000075B75E_9606	NA	NA	NA	NA

hsa-miR-6082	URS000075B120_9606	NA	NA	NA	NA
hsa-miR-8084	URS000075B7EF_9606	NA	NA	NA	NA
hsa-miR-3610	URS0000582627_9606	NA	NA	NA	NA
hsa-miR-10523-5p	URS0000D4EFF8_9606	NA	NA	NA	NA
hsa-miR-7849-3p	URS000075E1D7_9606	NA	NA	NA	NA
hsa-miR-4643	URS00002FBE0C_9606	NA	NA	NA	NA
hsa-miR-602	URS000075BFCD_9606	NA	NA	NA	NA
hsa-miR-4704-5p	URS00001996FD_9606	NA	NA	NA	NA
hsa-miR-4797-5p	URS00003BBE70_9606	NA	NA	NA	NA
hsa-miR-4262	URS0000759A3D_9606	NA	NA	NA	NA
hsa-miR-4523	URS00004B6231_9606	NA	NA	NA	NA
hsa-miR-619-3p	URS000075AB14_9606	NA	NA	NA	NA
hsa-miR-3654	URS000075C695_9606	NA	NA	NA	NA
hsa-miR-12126	URS0000D4F544_9606	NA	NA	NA	NA
hsa-miR-5680	URS000075A29E_9606	NA	NA	NA	NA
hsa-miR-4524b-3p	URS000075E1CB_9606	NA	NA	NA	NA
hsa-miR-4463	URS0000582334_9606	NA	NA	NA	NA
hsa-miR-6529-5p	URS0000D544A8_9606	NA	NA	NA	NA
hsa-miR-3908	URS000075D459_9606	NA	NA	NA	NA
hsa-miR-4690-5p	URS00005B6CAF_9606	NA	NA	NA	NA
hsa-miR-4676-5p	URS00005F4832_9606	NA	NA	NA	NA
hsa-miR-575	URS000075C5FB_9606	NA	NA	NA	NA
hsa-miR-4538	URS00007E4EFF_9606	NA	NA	NA	NA
hsa-miR-4453	URS000075B4E9_9606	NA	NA	NA	NA
hsa-miR-208a-5p	URS000005FEE9_9606	NA	NA	NA	NA
hsa-miR-4496	URS000056B180_9606	NA	NA	NA	NA
hsa-miR-12124	URS0000D505D2_9606	NA	NA	NA	NA
hsa-miR-1910-3p	URS000075A0DB_9606	NA	NA	NA	NA
hsa-miR-4478	URS00003FBC84_9606	NA	NA	NA	NA
hsa-miR-3929	URS00005BE56D_9606	NA	NA	NA	NA
hsa-miR-7843-5p	URS000075A92E_9606	NA	NA	NA	NA
hsa-miR-4632-5p	URS000075A071_9606	NA	NA	NA	NA
hsa-miR-1182	URS000075DDC1_9606	NA	NA	NA	NA
hsa-miR-515-3p	URS000075DC5B_9606	NA	NA	NA	NA
hsa-miR-6849-5p	URS000075D40D_9606	NA	NA	NA	NA
hsa-miR-1296-3p	URS000075B7D1_9606	NA	NA	NA	NA
hsa-miR-4280	URS000075AF03_9606	NA	NA	NA	NA
hsa-miR-6074	URS000075BE70_9606	NA	NA	NA	NA
hsa-miR-7151-5p	URS000075D1EC_9606	NA	NA	NA	NA

hsa-miR-1272	URS00000E1E9E_9606	NA	NA	NA	NA
hsa-miR-1322	URS000075DEC8_9606	NA	NA	NA	NA
hsa-miR-12129	URS0000D55A15_9606	NA	NA	NA	NA
hsa-miR-3919	URS000075D4D6_9606	NA	NA	NA	NA
hsa-miR-759	URS000075CCA8_9606	NA	NA	NA	NA
hsa-miR-1288-5p	URS00008E39F4_9606	NA	NA	NA	NA
hsa-miR-922	URS000075D35F_9606	NA	NA	NA	NA
hsa-miR-761	URS000075A6C8_9606	NA	NA	NA	NA
hsa-miR-4310	URS000075E10F_9606	NA	NA	NA	NA
hsa-miR-4436a	URS00004C6C6F_9606	NA	NA	NA	NA
hsa-miR-4269	URS000075EA8E_9606	NA	NA	NA	NA
hsa-miR-211-3p	URS0000044FD7_9606	NA	NA	NA	NA
hsa-miR-764	URS0000759DF8_9606	NA	NA	NA	NA
hsa-miR-5693	URS000075A448_9606	NA	NA	NA	NA
hsa-miR-4277	URS000075B86E_9606	NA	NA	NA	NA
hsa-miR-4289	URS0000253AF5_9606	NA	NA	NA	NA
hsa-miR-4707-5p	URS00003EB443_9606	NA	NA	NA	NA
hsa-miR-5587-3p	URS000075CC36_9606	NA	NA	NA	NA
hsa-miR-4740-3p	URS0000135D5B_9606	NA	NA	NA	NA
hsa-miR-1471	URS000075B0BA_9606	NA	NA	NA	NA
hsa-miR-1470	URS000075EBF5_9606	NA	NA	NA	NA
hsa-miR-2276-5p	URS000075E54C_9606	NA	NA	NA	NA
hsa-miR-4442	URS00003AD80B_9606	NA	NA	NA	NA
hsa-miR-6784-5p	URS000075F061_9606	NA	NA	NA	NA
hsa-miR-3927-5p	URS000075DAC9_9606	NA	NA	NA	NA
hsa-miR-4254	URS000075CCFE_9606	NA	NA	NA	NA
hsa-miR-320a-5p	URS000042A57E_9606	NA	NA	NA	NA
hsa-miR-375-5p	URS000075CCDA_9606	NA	NA	NA	NA
hsa-miR-4431	URS00002031FC_9606	NA	NA	NA	NA
hsa-miR-611	URS000075B74D_9606	NA	NA	NA	NA
hsa-miR-10392-5p	URS0000D53045_9606	NA	NA	NA	NA
hsa-miR-6125	URS000075F0F0_9606	NA	NA	NA	NA
hsa-miR-4285	URS000075E55E_9606	NA	NA	NA	NA
hsa-miR-4787-5p	URS0000521832_9606	NA	NA	NA	NA
hsa-miR-598-5p	URS000075F0D1_9606	NA	NA	NA	NA
hsa-miR-6786-5p	URS000075AD99_9606	NA	NA	NA	NA
hsa-miR-554	URS000075E66F_9606	NA	NA	NA	NA
hsa-miR-138-2-3p	URS000075AA94_9606	NA	NA	NA	NA
hsa-miR-4518	URS00004DEA91_9606	NA	NA	NA	NA

hsa-miR-6870-3p	URS000075EA66_9606	NA	NA	NA	NA
hsa-miR-3917	URS000035925E_9606	NA	NA	NA	NA
hsa-miR-301b-5p	URS000076F524_9606	NA	NA	NA	NA
hsa-miR-4539	URS00007E3F78_9606	NA	NA	NA	NA
hsa-miR-4502	URS0000498DAA_9606	NA	NA	NA	NA
hsa-miR-4734	URS00001ADE63_9606	NA	NA	NA	NA
hsa-miR-612	URS0000759916_9606	NA	NA	NA	NA
hsa-miR-4486	URS0000085D5C_9606	NA	NA	NA	NA
hsa-miR-3663-5p	URS000075ECFC_9606	NA	NA	NA	NA
hsa-miR-4299	URS000075ED80_9606	NA	NA	NA	NA
hsa-miR-3655	URS0000759BF4_9606	NA	NA	NA	NA
hsa-miR-539-5p	URS00003E59B7_9606	NA	NA	NA	NA
hsa-miR-1914-3p	URS000075E34C_9606	NA	NA	NA	NA
hsa-miR-6086	URS000075EDA9_9606	NA	NA	NA	NA
hsa-miR-4481	URS0000759B99_9606	NA	NA	NA	NA
hsa-miR-4534	URS000075B60F_9606	NA	NA	NA	NA
hsa-miR-8069	URS000075E1C1_9606	NA	NA	NA	NA
hsa-miR-4722-5p	URS000047996E_9606	NA	NA	NA	NA
hsa-miR-657	URS000075C4C7_9606	NA	NA	NA	NA
hsa-miR-4252	URS000075C911_9606	NA	NA	NA	NA
hsa-miR-10396b-3p	URS0000D54BA1_9606	NA	NA	NA	NA
hsa-miR-10396a-3p	URS0000D5616E_9606	NA	NA	NA	NA
hsa-miR-4312	URS000075A5FD_9606	NA	NA	NA	NA
hsa-miR-4783-5p	URS0000401970_9606	NA	NA	NA	NA
hsa-miR-658	URS00005A1F52_9606	NA	NA	NA	NA
hsa-miR-8072	URS000075AD9B_9606	NA	NA	NA	NA
hsa-miR-10396a-5p	URS0000D52B14_9606	NA	NA	NA	NA
hsa-miR-621	URS000075E0A8_9606	NA	NA	NA	NA
hsa-miR-6847-3p	URS000075EBAD_9606	NA	NA	NA	NA
hsa-miR-4268	URS000075D63F_9606	NA	NA	NA	NA
hsa-miR-8077	URS000075D913_9606	NA	NA	NA	NA
hsa-miR-3135b	URS0000164F91_9606	NA	NA	NA	NA
hsa-miR-5589-5p	URS0000759BC8_9606	NA	NA	NA	NA
hsa-miR-4327	URS0000759901_9606	NA	NA	NA	NA
hsa-miR-3160-5p	URS0000046A2A_9606	NA	NA	NA	NA
hsa-miR-6124	URS000075CC26_9606	NA	NA	NA	NA
hsa-miR-6077	URS000075B0B2_9606	NA	NA	NA	NA
hsa-miR-711	URS00004DC6C5_9606	NA	NA	NA	NA
hsa-miR-3689d	URS000075EE8F_9606	NA	NA	NA	NA

hsa-miR-4294	URS000075CB04_9606	NA	NA	NA	NA
hsa-miR-3201	URS000075A82C_9606	NA	NA	NA	NA
hsa-miR-1193	URS000002F2FA_9606	NA	NA	NA	NA
hsa-miR-4320	URS000075C783_9606	NA	NA	NA	NA
hsa-miR-6744-3p	URS000075EC03_9606	NA	NA	NA	NA
hsa-miR-6069	URS000075DD02_9606	NA	NA	NA	NA
hsa-miR-4284	URS00001FC26E_9606	NA	NA	NA	NA
hsa-miR-5787	URS000075CA3A_9606	NA	NA	NA	NA
hsa-miR-3153	URS0000192A75_9606	NA	NA	NA	NA
hsa-miR-8059	URS000075EE37_9606	NA	NA	NA	NA
hsa-miR-6090	URS0000759F58_9606	NA	NA	NA	NA
hsa-miR-920	URS000075EE93_9606	NA	NA	NA	NA
hsa-miR-2861	URS00003B13B8_9606	NA	NA	NA	NA
hsa-miR-3178	URS0000365675_9606	NA	NA	NA	NA
hsa-miR-762	URS0000327AFF_9606	NA	NA	NA	NA
hsa-miR-4675	URS000012C8F2_9606	NA	NA	NA	NA
hsa-miR-128-2-5p	URS000075ECF1_9606	NA	NA	NA	NA
hsa-miR-6752-5p	URS000075BFED_9606	NA	NA	NA	NA
hsa-miR-4483	URS000075DCE3_9606	NA	NA	NA	NA
hsa-miR-4281	URS00005C46A6_9606	NA	NA	NA	NA
hsa-miR-92a-2-5p	URS0000451A59_9606	NA	NA	NA	NA
hsa-miR-5087	URS000075E658_9606	NA	NA	NA	NA
hsa-miR-6073	URS000075A441_9606	NA	NA	NA	NA
hsa-miR-3713	URS000075A943_9606	NA	NA	NA	NA
hsa-miR-198	URS000075CAC3_9606	NA	NA	NA	NA
hsa-miR-489-5p	URS000075EF73_9606	NA	NA	NA	NA
hsa-miR-8078	URS000075A5F8_9606	NA	NA	NA	NA
hsa-miR-4316	URS000075C3BE_9606	NA	NA	NA	NA
hsa-miR-663b	URS000075C3F6_9606	NA	NA	NA	NA
hsa-miR-3151-5p	URS00002004E0_9606	NA	NA	NA	NA
hsa-miR-4447	URS000075E94B_9606	NA	NA	NA	NA
hsa-miR-4472	URS000029B04A_9606	NA	NA	NA	NA
hsa-miR-3147	URS00005BB0FD_9606	NA	NA	NA	NA
hsa-miR-4724-3p	URS00004C247E_9606	NA	NA	NA	NA
hsa-miR-5682	URS000075D612_9606	NA	NA	NA	NA
hsa-miR-6824-5p	URS000075B3A8_9606	NA	NA	NA	NA
hsa-miR-6789-5p	URS000075DD04_9606	NA	NA	NA	NA
hsa-miR-6800-5p	URS000075AFAE_9606	NA	NA	NA	NA
hsa-miR-4789-5p	URS000002D005_9606	NA	NA	NA	NA

hsa-miR-4420	URS000075EE0B_9606	NA	NA	NA	NA
hsa-miR-4436b-5p	URS0000577194_9606	NA	NA	NA	NA
hsa-miR-670-5p	URS000075D4D2_9606	NA	NA	NA	NA
hsa-miR-4691-5p	URS00001067D1_9606	NA	NA	NA	NA
hsa-miR-11181-5p	URS000050D64A_9606	NA	NA	NA	NA
hsa-miR-6126	URS000075D118_9606	NA	NA	NA	NA
hsa-miR-4439	URS000075E7F6_9606	NA	NA	NA	NA
hsa-miR-6864-3p	URS000075C193_9606	NA	NA	NA	NA
hsa-miR-6858-5p	URS000075C360_9606	NA	NA	NA	NA
hsa-miR-6765-5p	URS000075D0AA_9606	NA	NA	NA	NA
hsa-miR-937-5p	URS0000776393_9606	NA	NA	NA	NA
hsa-miR-1238-5p	URS000075EC66_9606	NA	NA	NA	NA
hsa-miR-4758-5p	URS0000378EED_9606	NA	NA	NA	NA
hsa-miR-6790-5p	URS000075A707_9606	NA	NA	NA	NA
hsa-miR-10395-5p	URS0000D53F1E_9606	NA	NA	NA	NA
hsa-miR-5011-3p	URS000075EB49_9606	NA	NA	NA	NA
hsa-miR-4715-3p	URS00000037E8_9606	NA	NA	NA	NA
hsa-miR-1202	URS000075E909_9606	NA	NA	NA	NA
hsa-miR-3977	URS000075BDA6_9606	NA	NA	NA	NA
hsa-miR-3978	URS000075C7BE_9606	NA	NA	NA	NA
hsa-miR-4776-5p	URS00001C978C_9606	NA	NA	NA	NA
hsa-miR-4535	URS000075DF88_9606	NA	NA	NA	NA
hsa-miR-8057	URS000075C349_9606	NA	NA	NA	NA
hsa-miR-647	URS000075B269_9606	NA	NA	NA	NA
hsa-miR-5089-5p	URS000075A7A4_9606	NA	NA	NA	NA
hsa-miR-1227-5p	URS0000773D7C_9606	NA	NA	NA	NA
hsa-miR-3940-5p	URS00001E8DA7_9606	NA	NA	NA	NA
hsa-miR-487b-5p	URS000075B2DC_9606	NA	NA	NA	NA
hsa-miR-632	URS000075C639_9606	NA	NA	NA	NA
hsa-miR-1231	URS000075CC1D_9606	NA	NA	NA	NA
hsa-miR-7108-5p	URS000075A4B1_9606	NA	NA	NA	NA
hsa-miR-147a	URS00002849B7_9606	NA	NA	NA	NA
hsa-miR-2053	URS000075C1AA_9606	NA	NA	NA	NA
hsa-miR-4273	URS000075A3CA_9606	NA	NA	NA	NA
hsa-miR-2909	URS000075B640_9606	NA	NA	NA	NA
hsa-miR-607	URS0000759A61_9606	NA	NA	NA	NA
hsa-miR-3692-3p	URS000075BD86_9606	NA	NA	NA	NA
hsa-miR-4652-3p	URS00000AFFDD_9606	NA	NA	NA	NA
hsa-miR-3122	URS000032AE90_9606	NA	NA	NA	NA

hsa-miR-5572	URS000075D1DF_9606	NA	NA	NA	NA
hsa-miR-599	URS000075A123_9606	NA	NA	NA	NA
hsa-miR-557	URS000075C267_9606	NA	NA	NA	NA
hsa-miR-2681-5p	URS0000759EBF_9606	NA	NA	NA	NA
hsa-miR-548x-3p	URS000043D2C1_9606	NA	NA	NA	NA
hsa-miR-548aj-3p	URS000007A1BB_9606	NA	NA	NA	NA
hsa-miR-548as-3p	URS000075AD19_9606	NA	NA	NA	NA
hsa-miR-548ar-3p	URS00004283FE_9606	NA	NA	NA	NA
hsa-miR-4282	URS000075AC52_9606	NA	NA	NA	NA
hsa-miR-7161-5p	URS0000759DEF_9606	NA	NA	NA	NA
hsa-miR-559	URS000075AE29_9606	NA	NA	NA	NA
hsa-miR-4796-3p	URS000025EABA_9606	NA	NA	NA	NA
hsa-miR-605-5p	URS000075CCFA_9606	NA	NA	NA	NA
hsa-miR-5688	URS000075ACD8_9606	NA	NA	NA	NA
hsa-miR-12120	URS0000D5666B_9606	NA	NA	NA	NA
hsa-miR-3936	URS000058DCD8_9606	NA	NA	NA	NA
hsa-miR-302e	URS000075E566_9606	NA	NA	NA	NA
hsa-miR-302c-3p	URS000027080C_9606	NA	NA	NA	NA
hsa-miR-302d-3p	URS000041E949_9606	NA	NA	NA	NA
hsa-miR-302b-3p	URS0000346991_9606	NA	NA	NA	NA
hsa-miR-302a-3p	URS0000070CD2_9606	NA	NA	NA	NA
hsa-miR-580-5p	URS00008E39A0_9606	NA	NA	NA	NA
hsa-miR-5700	URS000075CB22_9606	NA	NA	NA	NA
hsa-miR-302f	URS000075D4BB_9606	NA	NA	NA	NA
hsa-miR-4801	URS000020B655_9606	NA	NA	NA	NA
hsa-miR-5683	URS000075E023_9606	NA	NA	NA	NA
hsa-miR-6856-3p	URS000075EC61_9606	NA	NA	NA	NA
hsa-miR-6512-5p	URS000075DDC9_9606	NA	NA	NA	NA
hsa-miR-433-5p	URS000025F226_9606	NA	NA	NA	NA
hsa-miR-4637	URS000036C820_9606	NA	NA	NA	NA
hsa-miR-5003-3p	URS000075A132_9606	NA	NA	NA	NA
hsa-miR-6502-3p	URS000075B62B_9606	NA	NA	NA	NA
hsa-miR-6863	URS0000759FE7_9606	NA	NA	NA	NA
hsa-miR-12127	URS0000D549A8_9606	NA	NA	NA	NA
hsa-miR-5007-5p	URS000075D494_9606	NA	NA	NA	NA
hsa-miR-577	URS00004CD810_9606	NA	NA	NA	NA
hsa-miR-7161-3p	URS000075E7DE_9606	NA	NA	NA	NA
hsa-miR-548p	URS00002E628F_9606	NA	NA	NA	NA
hsa-miR-451b	URS000022EC48_9606	NA	NA	NA	NA

hsa-miR-4703-5p	URS00001B42CA_9606	NA	NA	NA	NA
hsa-miR-4781-5p	URS00000913AC_9606	NA	NA	NA	NA
hsa-miR-4759	URS00005ED2F1_9606	NA	NA	NA	NA
hsa-miR-3169	URS000075C0C6_9606	NA	NA	NA	NA
hsa-miR-4779	URS00005D4237_9606	NA	NA	NA	NA
hsa-miR-2392	URS00003F7488_9606	NA	NA	NA	NA
hsa-miR-5582-5p	URS000075A76C_9606	NA	NA	NA	NA
hsa-miR-4717-5p	URS000025DD6B_9606	NA	NA	NA	NA
hsa-miR-6757-5p	URS0000759C08_9606	NA	NA	NA	NA
hsa-miR-6732-5p	URS000075DB80_9606	NA	NA	NA	NA
hsa-miR-6782-5p	URS000075C94B_9606	NA	NA	NA	NA
hsa-miR-6841-5p	URS000075C704_9606	NA	NA	NA	NA
hsa-miR-6755-5p	URS000075D344_9606	NA	NA	NA	NA
hsa-miR-12115	URS0000D5512C_9606	NA	NA	NA	NA
hsa-miR-3681-5p	URS000075CD8F_9606	NA	NA	NA	NA
hsa-miR-6718-5p	URS0000759F79_9606	NA	NA	NA	NA
hsa-miR-203b-5p	URS00005ED615_9606	NA	NA	NA	NA
hsa-miR-5584-3p	URS000075A021_9606	NA	NA	NA	NA
hsa-miR-3163	URS00003A5E54_9606	NA	NA	NA	NA
hsa-miR-300	URS000075CFC2_9606	NA	NA	NA	NA
hsa-miR-875-5p	URS0000312ECD_9606	NA	NA	NA	NA
hsa-miR-3976	URS000075D2E1_9606	NA	NA	NA	NA
hsa-miR-5011-5p	URS000075C2FA_9606	NA	NA	NA	NA
hsa-miR-8076	URS000075A244_9606	NA	NA	NA	NA
hsa-miR-4686	URS000059225F_9606	NA	NA	NA	NA
hsa-miR-586	URS000075D29B_9606	NA	NA	NA	NA
hsa-miR-5579-5p	URS000075A46D_9606	NA	NA	NA	NA
hsa-miR-411-3p	URS000037DAEA_9606	NA	NA	NA	NA
hsa-miR-380-3p	URS00002CAE0C_9606	NA	NA	NA	NA
hsa-miR-7852-3p	URS000075B643_9606	NA	NA	NA	NA
hsa-miR-6834-3p	URS000075C061_9606	NA	NA	NA	NA
hsa-miR-12132	URS0000D503F8_9606	NA	NA	NA	NA
hsa-miR-892c-5p	URS000075B420_9606	NA	NA	NA	NA
hsa-miR-506-5p	URS000051D911_9606	NA	NA	NA	NA
hsa-miR-1323	URS000058276F_9606	NA	NA	NA	NA
hsa-miR-8063	URS0000759D19_9606	NA	NA	NA	NA
hsa-miR-4698	URS00001F3F1D_9606	NA	NA	NA	NA
hsa-miR-7853-5p	URS0000759FA4_9606	NA	NA	NA	NA
hsa-miR-3922-5p	URS00004D1EF6_9606	NA	NA	NA	NA

hsa-miR-5003-5p	URS000075A6AE_9606	NA	NA	NA	NA
hsa-miR-3622b-3p	URS000075EAAB_9606	NA	NA	NA	NA
hsa-miR-6765-3p	URS000075BB8F_9606	NA	NA	NA	NA
hsa-miR-4999-3p	URS000075BC86_9606	NA	NA	NA	NA
hsa-miR-4639-3p	URS0000465D9A_9606	NA	NA	NA	NA
hsa-miR-1208	URS000075B904_9606	NA	NA	NA	NA
hsa-miR-1245b-3p	URS00001C8C4E_9606	NA	NA	NA	NA
hsa-miR-3619-5p	URS000053966D_9606	NA	NA	NA	NA
hsa-miR-7157-5p	URS000075E5FE_9606	NA	NA	NA	NA
hsa-miR-6736-3p	URS0000759B63_9606	NA	NA	NA	NA
hsa-miR-1207-3p	URS000075CE03_9606	NA	NA	NA	NA
hsa-miR-4692	URS000036BD40_9606	NA	NA	NA	NA
hsa-miR-1972	URS000042A1A2_9606	NA	NA	NA	NA
hsa-miR-4650-5p	URS0000474B6E_9606	NA	NA	NA	NA
hsa-miR-6823-5p	URS000075C050_9606	NA	NA	NA	NA
hsa-miR-4704-3p	URS00003E272F_9606	NA	NA	NA	NA
hsa-miR-103b	URS000075C8F4_9606	NA	NA	NA	NA
hsa-miR-1279	URS000075A375_9606	NA	NA	NA	NA
hsa-miR-4528	URS00007E3FAB_9606	NA	NA	NA	NA
hsa-miR-153-5p	URS000075C325_9606	NA	NA	NA	NA
hsa-miR-4267	URS000075CCB3_9606	NA	NA	NA	NA
hsa-miR-4673	URS0000316834_9606	NA	NA	NA	NA
hsa-miR-4712-5p	URS00004B94AF_9606	NA	NA	NA	NA
hsa-miR-770-5p	URS000075A169_9606	NA	NA	NA	NA
hsa-miR-1825	URS000075AF4A_9606	NA	NA	NA	NA
hsa-miR-6814-5p	URS000075E7CD_9606	NA	NA	NA	NA
hsa-miR-662	URS000075C986_9606	NA	NA	NA	NA
hsa-miR-4301	URS000075DDFA_9606	NA	NA	NA	NA
hsa-miR-6887-3p	URS000075EA2A_9606	NA	NA	NA	NA
hsa-miR-6756-3p	URS000075D237_9606	NA	NA	NA	NA
hsa-miR-3127-3p	URS0000292363_9606	NA	NA	NA	NA
hsa-miR-3162-3p	URS0000157FBE_9606	NA	NA	NA	NA
hsa-miR-6892-3p	URS000075C294_9606	NA	NA	NA	NA
hsa-miR-4319	URS000075ED91_9606	NA	NA	NA	NA
hsa-miR-4308	URS000075A774_9606	NA	NA	NA	NA
hsa-miR-5088-3p	URS000075C587_9606	NA	NA	NA	NA
hsa-miR-3678-5p	URS000075A9CF_9606	NA	NA	NA	NA
hsa-miR-5009-3p	URS000075D1F6_9606	NA	NA	NA	NA
hsa-miR-6761-3p	URS000075BA43_9606	NA	NA	NA	NA

hsa-miR-6072	URS000075CE24_9606	NA	NA	NA	NA
hsa-miR-6727-3p	URS000075DDC6_9606	NA	NA	NA	NA
hsa-miR-1539	URS000075C7DF_9606	NA	NA	NA	NA
hsa-miR-6748-3p	URS000075E2F1_9606	NA	NA	NA	NA
hsa-miR-5699-3p	URS0000331800_9606	NA	NA	NA	NA
hsa-miR-3121-5p	URS000055AE24_9606	NA	NA	NA	NA
hsa-miR-196b-3p	URS000052E1A6_9606	NA	NA	NA	NA
hsa-miR-1281	URS000075E7A3_9606	NA	NA	NA	NA
hsa-miR-1292-3p	URS000077ED64_9606	NA	NA	NA	NA
hsa-miR-7107-5p	URS000075D394_9606	NA	NA	NA	NA
hsa-miR-6749-5p	URS0000759E9A_9606	NA	NA	NA	NA
hsa-miR-6499-5p	URS000075C4B1_9606	NA	NA	NA	NA
hsa-miR-6775-5p	URS0000759DC2_9606	NA	NA	NA	NA
hsa-miR-6506-3p	URS000075DC71_9606	NA	NA	NA	NA
hsa-miR-4536-3p	URS0000111E5E_9606	NA	NA	NA	NA
hsa-miR-1204	URS000075E520_9606	NA	NA	NA	NA
hsa-miR-1282	URS000075E8C2_9606	NA	NA	NA	NA
hsa-miR-1283	URS0000552112_9606	NA	NA	NA	NA
hsa-miR-6508-5p	URS000075D928_9606	NA	NA	NA	NA
hsa-miR-520g-5p	URS000075EBEC_9606	NA	NA	NA	NA
hsa-miR-645	URS000075E7D0_9606	NA	NA	NA	NA
hsa-miR-6080	URS0000759B11_9606	NA	NA	NA	NA
hsa-miR-4287	URS000075DF76_9606	NA	NA	NA	NA
hsa-miR-12125	URS0000D51DF6_9606	NA	NA	NA	NA
hsa-miR-7110-3p	URS000075CF8A_9606	NA	NA	NA	NA
hsa-miR-518c-5p	URS000003B660_9606	NA	NA	NA	NA
hsa-miR-6764-3p	URS000075CD40_9606	NA	NA	NA	NA
hsa-miR-4766-5p	URS00000292B1_9606	NA	NA	NA	NA
hsa-miR-4427	URS000075C315_9606	NA	NA	NA	NA
hsa-miR-6719-3p	URS000075A32A_9606	NA	NA	NA	NA
hsa-miR-7162-3p	URS000075EA0F_9606	NA	NA	NA	NA
hsa-miR-4682	URS0000348C0E_9606	NA	NA	NA	NA
hsa-miR-4793-3p	URS00001A1CA6_9606	NA	NA	NA	NA
hsa-miR-1205	URS0000759FAE_9606	NA	NA	NA	NA
hsa-miR-10394-5p	URS0000D53067_9606	NA	NA	NA	NA
hsa-miR-6817-5p	URS00007599D0_9606	NA	NA	NA	NA
hsa-miR-4769-3p	URS000045D31D_9606	NA	NA	NA	NA
hsa-miR-1913	URS000075C082_9606	NA	NA	NA	NA
hsa-miR-767-3p	URS000050F899_9606	NA	NA	NA	NA

hsa-miR-6839-5p	URS000075B938_9606	NA	NA	NA	NA
hsa-miR-3128	URS00002AA55A_9606	NA	NA	NA	NA
hsa-miR-3190-5p	URS00007857FE_9606	NA	NA	NA	NA
hsa-miR-3922-3p	URS00004CD06B_9606	NA	NA	NA	NA
hsa-miR-6504-5p	URS000075B71A_9606	NA	NA	NA	NA
hsa-miR-7155-5p	URS000075ADD2_9606	NA	NA	NA	NA
hsa-miR-6776-5p	URS000075C4D0_9606	NA	NA	NA	NA
hsa-miR-4490	URS000075ABA2_9606	NA	NA	NA	NA
hsa-miR-7152-3p	URS000075E016_9606	NA	NA	NA	NA
hsa-miR-7978	URS000075C403_9606	NA	NA	NA	NA
hsa-miR-3137	URS0000427B2B_9606	NA	NA	NA	NA
hsa-miR-215-3p	URS000075D75C_9606	NA	NA	NA	NA
hsa-miR-4679	URS00002F0692_9606	NA	NA	NA	NA
hsa-miR-6889-3p	URS000075D99E_9606	NA	NA	NA	NA
hsa-miR-7114-5p	URS000075E8C6_9606	NA	NA	NA	NA
hsa-miR-581	URS000075A12F_9606	NA	NA	NA	NA
hsa-miR-4786-3p	URS0000078918_9606	NA	NA	NA	NA
hsa-miR-4790-3p	URS00005CA611_9606	NA	NA	NA	NA
hsa-miR-4462	URS000075CB43_9606	NA	NA	NA	NA
hsa-miR-7114-3p	URS0000759FDB_9606	NA	NA	NA	NA
hsa-miR-6846-3p	URS000075C264_9606	NA	NA	NA	NA
hsa-miR-6821-3p	URS000075C3E6_9606	NA	NA	NA	NA
hsa-miR-6759-3p	URS000075B2EE_9606	NA	NA	NA	NA
hsa-miR-6831-3p	URS000075A9CE_9606	NA	NA	NA	NA
hsa-miR-10525-3p	URS0000D520C0_9606	NA	NA	NA	NA
hsa-miR-4522	URS000075DEFC_9606	NA	NA	NA	NA
hsa-miR-6857-3p	URS000075B016_9606	NA	NA	NA	NA
hsa-miR-5002-3p	URS000075DA7B_9606	NA	NA	NA	NA
hsa-miR-7153-5p	URS000075AAA0_9606	NA	NA	NA	NA
hsa-miR-6770-5p	URS000075A6A9_9606	NA	NA	NA	NA
hsa-miR-4786-5p	URS000023BDD3_9606	NA	NA	NA	NA
hsa-miR-4693-3p	URS00004A6747_9606	NA	NA	NA	NA
hsa-miR-4770	URS0000097E9E_9606	NA	NA	NA	NA
hsa-miR-3663-3p	URS000075C2EC_9606	NA	NA	NA	NA
hsa-miR-1225-3p	URS000075D62D_9606	NA	NA	NA	NA
hsa-miR-1233-3p	URS000075D36A_9606	NA	NA	NA	NA
hsa-miR-4537	URS00007E385E_9606	NA	NA	NA	NA
hsa-miR-6823-3p	URS000075DEC2_9606	NA	NA	NA	NA
hsa-miR-558	URS000075ED39_9606	NA	NA	NA	NA

hsa-miR-5004-5p	URS000054A671_9606	NA	NA	NA	NA
hsa-miR-4784	URS000021E7E5_9606	NA	NA	NA	NA
hsa-miR-3975	URS000075A627_9606	NA	NA	NA	NA
hsa-miR-6133	URS000075BE4E_9606	NA	NA	NA	NA
hsa-miR-6127	URS000075F03F_9606	NA	NA	NA	NA
hsa-miR-6129	URS000075DA70_9606	NA	NA	NA	NA
hsa-miR-4721	URS000036D67C_9606	NA	NA	NA	NA
hsa-miR-3184-5p	URS000075CC75_9606	NA	NA	NA	NA
hsa-miR-5194	URS0000585553_9606	NA	NA	NA	NA
hsa-miR-6804-5p	URS000075E381_9606	NA	NA	NA	NA
hsa-miR-4500	URS000001953B_9606	NA	NA	NA	NA
hsa-miR-496	URS000003BF62_9606	NA	NA	NA	NA
hsa-miR-1236-5p	URS00008E3A19_9606	NA	NA	NA	NA
hsa-miR-4765	URS0000586DB1_9606	NA	NA	NA	NA
hsa-miR-1909-5p	URS000075C862_9606	NA	NA	NA	NA
hsa-miR-4745-5p	URS00005485E8_9606	NA	NA	NA	NA
hsa-miR-571	URS000075C61C_9606	NA	NA	NA	NA
hsa-miR-4772-5p	URS0000411752_9606	NA	NA	NA	NA
hsa-miR-4695-3p	URS00005895E8_9606	NA	NA	NA	NA
hsa-miR-8082	URS000075EC91_9606	NA	NA	NA	NA
hsa-miR-3134	URS000053B1E8_9606	NA	NA	NA	NA
hsa-miR-4782-3p	URS0000064E13_9606	NA	NA	NA	NA
hsa-miR-6766-3p	URS000075DCFB_9606	NA	NA	NA	NA
hsa-miR-548f-5p	URS000075B630_9606	NA	NA	NA	NA
hsa-miR-4696	URS00002F59AC_9606	NA	NA	NA	NA
hsa-miR-5589-3p	URS000075C293_9606	NA	NA	NA	NA
hsa-miR-6722-3p	URS000075A2AF_9606	NA	NA	NA	NA
hsa-miR-7112-3p	URS000075C91C_9606	NA	NA	NA	NA
hsa-miR-4711-5p	URS0000397A01_9606	NA	NA	NA	NA
hsa-miR-4641	URS0000307985_9606	NA	NA	NA	NA
hsa-miR-3189-5p	URS000054ED3F_9606	NA	NA	NA	NA
hsa-miR-4290	URS000075B42A_9606	NA	NA	NA	NA
hsa-miR-6799-3p	URS000075D4BE_9606	NA	NA	NA	NA
hsa-miR-938	URS000075DF80_9606	NA	NA	NA	NA
hsa-miR-6886-3p	URS000075D6A4_9606	NA	NA	NA	NA
hsa-miR-4632-3p	URS000075A2F8_9606	NA	NA	NA	NA
hsa-miR-24-1-5p	URS00002D0FC3_9606	NA	NA	NA	NA
hsa-miR-3135a	URS0000411672_9606	NA	NA	NA	NA
hsa-miR-6778-3p	URS000075C1BB_9606	NA	NA	NA	NA

hsa-miR-6829-3p	URS000075C970_9606	NA	NA	NA	NA
hsa-miR-661	URS000075A4E8_9606	NA	NA	NA	NA
hsa-miR-4297	URS000075DF06_9606	NA	NA	NA	NA
hsa-miR-3683	URS000075E3A1_9606	NA	NA	NA	NA
hsa-miR-668-5p	URS000075A144_9606	NA	NA	NA	NA
hsa-miR-1199-3p	URS000075F0BC_9606	NA	NA	NA	NA
hsa-miR-6854-3p	URS000075CB83_9606	NA	NA	NA	NA
hsa-miR-8086	URS000075CB45_9606	NA	NA	NA	NA
hsa-miR-7160-5p	URS000075AAFD_9606	NA	NA	NA	NA
hsa-miR-8075	URS000075A38E_9606	NA	NA	NA	NA
hsa-miR-6882-3p	URS000075C8AB_9606	NA	NA	NA	NA
hsa-miR-4731-5p	URS00005E08E8_9606	NA	NA	NA	NA
hsa-miR-4720-3p	URS000039B339_9606	NA	NA	NA	NA
hsa-miR-516b-3p	URS0000205A39_9606	NA	NA	NA	NA
hsa-miR-7162-5p	URS000075B7A9_9606	NA	NA	NA	NA
hsa-miR-3148	URS0000308E4D_9606	NA	NA	NA	NA
hsa-miR-6861-3p	URS000075D08E_9606	NA	NA	NA	NA
hsa-miR-1289	URS0000030489_9606	NA	NA	NA	NA
hsa-miR-4791	URS000002CF68_9606	NA	NA	NA	NA
hsa-miR-6744-5p	URS000075E254_9606	NA	NA	NA	NA
hsa-miR-4764-5p	URS0000103638_9606	NA	NA	NA	NA
hsa-miR-9851-3p	URS0000D562D3_9606	NA	NA	NA	NA
hsa-miR-1207-5p	URS000055C019_9606	NA	NA	NA	NA
hsa-miR-548au-3p	URS000075AEA5_9606	NA	NA	NA	NA
hsa-miR-3136-3p	URS00003D0904_9606	NA	NA	NA	NA
hsa-miR-7155-3p	URS000075D584_9606	NA	NA	NA	NA
hsa-miR-4743-5p	URS0000117FAF_9606	NA	NA	NA	NA
hsa-miR-8053	URS000075B09C_9606	NA	NA	NA	NA
hsa-miR-6787-5p	URS000075DD35_9606	NA	NA	NA	NA
hsa-miR-6762-3p	URS000075CFC5_9606	NA	NA	NA	NA
hsa-miR-3119	URS0000201018_9606	NA	NA	NA	NA
hsa-miR-6739-5p	URS000075C51C_9606	NA	NA	NA	NA
hsa-miR-4471	URS000056AA01_9606	NA	NA	NA	NA
hsa-miR-6716-5p	URS000075DC9C_9606	NA	NA	NA	NA
hsa-miR-8085	URS000075B084_9606	NA	NA	NA	NA
hsa-miR-5591-5p	URS000075C971_9606	NA	NA	NA	NA
hsa-miR-4300	URS000075A674_9606	NA	NA	NA	NA
hsa-miR-7106-5p	URS000075AD04_9606	NA	NA	NA	NA
hsa-miR-4713-3p	URS00005CB08D_9606	NA	NA	NA	NA

hsa-miR-6508-3p	URS000075AA33_9606	NA	NA	NA	NA
hsa-miR-4649-5p	URS000044FB51_9606	NA	NA	NA	NA
hsa-miR-4437	URS000075EB18_9606	NA	NA	NA	NA
hsa-miR-4656	URS00003F7B61_9606	NA	NA	NA	NA
hsa-miR-6829-5p	URS000075D8A1_9606	NA	NA	NA	NA
hsa-miR-4498	URS000027F5F3_9606	NA	NA	NA	NA
hsa-miR-6780b-5p	URS000075E471_9606	NA	NA	NA	NA
hsa-miR-3191-3p	URS0000116F83_9606	NA	NA	NA	NA
hsa-miR-6825-5p	URS0000759D52_9606	NA	NA	NA	NA
hsa-miR-6816-5p	URS000075C63F_9606	NA	NA	NA	NA
hsa-miR-4283	URS0000759EB4_9606	NA	NA	NA	NA
hsa-miR-6870-5p	URS000075D04E_9606	NA	NA	NA	NA
hsa-miR-4723-5p	URS000004BACE_9606	NA	NA	NA	NA
hsa-miR-7111-5p	URS000075EE2D_9606	NA	NA	NA	NA
hsa-miR-6846-5p	URS000019D324_9606	NA	NA	NA	NA
hsa-miR-6848-5p	URS000075E309_9606	NA	NA	NA	NA
hsa-miR-6887-5p	URS000075BEF5_9606	NA	NA	NA	NA
hsa-miR-6795-5p	URS000075BAA5_9606	NA	NA	NA	NA
hsa-miR-7110-5p	URS000075A075_9606	NA	NA	NA	NA
hsa-miR-3132	URS000053C4D7_9606	NA	NA	NA	NA
hsa-miR-6772-5p	URS000075990B_9606	NA	NA	NA	NA
hsa-miR-4451	URS000037E170_9606	NA	NA	NA	NA
hsa-miR-6801-5p	URS000075D708_9606	NA	NA	NA	NA
hsa-miR-10393-3p	URS0000D535D5_9606	NA	NA	NA	NA
hsa-miR-601	URS000075C340_9606	NA	NA	NA	NA
hsa-miR-4527	URS000075E48E_9606	NA	NA	NA	NA
hsa-miR-6753-3p	URS000075A4C6_9606	NA	NA	NA	NA
hsa-miR-7107-3p	URS0000759FB2_9606	NA	NA	NA	NA
hsa-miR-6880-5p	URS000075EE40_9606	NA	NA	NA	NA
hsa-miR-541-3p	URS000075A3AC_9606	NA	NA	NA	NA
hsa-miR-654-5p	URS00002B0B46_9606	NA	NA	NA	NA
hsa-miR-6769b-5p	URS000075C6AD_9606	NA	NA	NA	NA
hsa-miR-876-3p	URS00002E8D60_9606	NA	NA	NA	NA
hsa-miR-3935	URS000075E0F1_9606	NA	NA	NA	NA
hsa-miR-8065	URS000075B817_9606	NA	NA	NA	NA
hsa-miR-6806-5p	URS000075D836_9606	NA	NA	NA	NA
hsa-miR-4703-3p	URS00003AFECC_9606	NA	NA	NA	NA
hsa-miR-12122	URS0000D4F007_9606	NA	NA	NA	NA
hsa-miR-668-3p	URS000075C6C5_9606	NA	NA	NA	NA

hsa-miR-6852-3p	URS000075E354_9606	NA	NA	NA	NA
hsa-miR-3618	URS000075E3B9_9606	NA	NA	NA	NA
hsa-miR-4796-5p	URS00003BED7E_9606	NA	NA	NA	NA
hsa-miR-6895-3p	URS000075EC02_9606	NA	NA	NA	NA
hsa-miR-593-3p	URS000075D407_9606	NA	NA	NA	NA
hsa-miR-6740-3p	URS000075CDE3_9606	NA	NA	NA	NA
hsa-miR-6830-3p	URS0000759D6B_9606	NA	NA	NA	NA
hsa-miR-4504	URS000075AB46_9606	NA	NA	NA	NA
hsa-miR-7973	URS000075C1F6_9606	NA	NA	NA	NA
hsa-miR-9986	URS0000D54710_9606	NA	NA	NA	NA
hsa-miR-3689a-5p	URS000075D33D_9606	NA	NA	NA	NA
hsa-miR-3689f	URS000075A613_9606	NA	NA	NA	NA
hsa-miR-3944-5p	URS00002ED2C9_9606	NA	NA	NA	NA
hsa-miR-4648	URS00001F7D99_9606	NA	NA	NA	NA
hsa-miR-3177-5p	URS0000534224_9606	NA	NA	NA	NA
hsa-miR-3152-3p	URS000029E08E_9606	NA	NA	NA	NA
hsa-miR-1206	URS000075DD83_9606	NA	NA	NA	NA
hsa-miR-2117	URS000075E03C_9606	NA	NA	NA	NA
hsa-miR-4425	URS000035AC91_9606	NA	NA	NA	NA
hsa-miR-5705	URS000075EB7D_9606	NA	NA	NA	NA
hsa-miR-8068	URS000075C6A8_9606	NA	NA	NA	NA
hsa-miR-2052	URS000075D81D_9606	NA	NA	NA	NA
hsa-miR-4764-3p	URS000075CBE1_9606	NA	NA	NA	NA
hsa-miR-5687	URS000075E517_9606	NA	NA	NA	NA
hsa-miR-4321	URS000075C164_9606	NA	NA	NA	NA
hsa-miR-5579-3p	URS000075E056_9606	NA	NA	NA	NA
hsa-miR-5704	URS000075E6A7_9606	NA	NA	NA	NA
hsa-miR-12116	URS0000D5425A_9606	NA	NA	NA	NA
hsa-miR-3162-5p	URS00001E2938_9606	NA	NA	NA	NA
hsa-miR-4540	URS000075CD2F_9606	NA	NA	NA	NA
hsa-miR-4474-5p	URS00002B237F_9606	NA	NA	NA	NA
hsa-miR-4671-3p	URS00005DEA88_9606	NA	NA	NA	NA
hsa-miR-12123	URS0000D565FA_9606	NA	NA	NA	NA
hsa-miR-5701	URS00000EA6BC_9606	NA	NA	NA	NA
hsa-miR-7159-5p	URS000075C337_9606	NA	NA	NA	NA
hsa-miR-6802-3p	URS000075A75D_9606	NA	NA	NA	NA
hsa-miR-5100	URS0000079F78_9606	NA	NA	NA	NA
hsa-miR-4291	URS00002FB584_9606	NA	NA	NA	NA
hsa-miR-12128	URS0000D50273_9606	NA	NA	NA	NA

hsa-miR-11399	URS0000D52A68_9606	NA	NA	NA	NA
hsa-miR-7154-5p	URS000075D02B_9606	NA	NA	NA	NA
hsa-miR-6509-3p	URS000075D8E0_9606	NA	NA	NA	NA
hsa-miR-6720-5p	URS000075ED75_9606	NA	NA	NA	NA
hsa-miR-6512-3p	URS000075CC78_9606	NA	NA	NA	NA
hsa-miR-6084	URS000075A98B_9606	NA	NA	NA	NA
hsa-miR-4777-5p	URS000047F4E3_9606	NA	NA	NA	NA
hsa-miR-515-5p	URS00000A68B2_9606	NA	NA	NA	NA
hsa-miR-519e-5p	URS000075AC86_9606	NA	NA	NA	NA
hsa-miR-4713-5p	URS00004F12D0_9606	NA	NA	NA	NA
hsa-miR-4303	URS000075AF7F_9606	NA	NA	NA	NA
hsa-miR-12119	URS0000D5215D_9606	NA	NA	NA	NA
hsa-miR-6071	URS000075A4D5_9606	NA	NA	NA	NA
hsa-miR-5706	URS0000381CEC_9606	NA	NA	NA	NA
hsa-miR-4782-5p	URS00000401F7_9606	NA	NA	NA	NA
hsa-miR-6844	URS000075C4CD_9606	NA	NA	NA	NA
hsa-miR-6864-5p	URS000075E86B_9606	NA	NA	NA	NA
hsa-miR-4452	URS000075A9FF_9606	NA	NA	NA	NA
hsa-miR-3915	URS0000458AD2_9606	NA	NA	NA	NA
hsa-miR-4325	URS000075EFFA_9606	NA	NA	NA	NA
hsa-miR-5047	URS000075CFDE_9606	NA	NA	NA	NA
hsa-miR-4666b	URS00007599A3_9606	NA	NA	NA	NA
hsa-miR-5006-5p	URS000075CF59_9606	NA	NA	NA	NA
hsa-miR-5590-5p	URS000075E858_9606	NA	NA	NA	NA
hsa-miR-449c-3p	URS000075DBB5_9606	NA	NA	NA	NA
hsa-miR-1178-3p	URS000075AB23_9606	NA	NA	NA	NA
hsa-miR-5691	URS000075A37B_9606	NA	NA	NA	NA
hsa-miR-6805-3p	URS000075D13A_9606	NA	NA	NA	NA
hsa-miR-9718	URS0000D53E6B_9606	NA	NA	NA	NA
hsa-miR-6079	URS0000759C18_9606	NA	NA	NA	NA
hsa-miR-588	URS000075CAF8_9606	NA	NA	NA	NA
hsa-miR-4701-5p	URS000021FE20_9606	NA	NA	NA	NA
hsa-miR-1302	URS0000442977_9606	NA	NA	NA	NA
hsa-miR-450b-3p	URS00002FF522_9606	NA	NA	NA	NA
hsa-miR-6728-5p	URS000075A56F_9606	NA	NA	NA	NA
hsa-miR-6773-5p	URS0000759BD9_9606	NA	NA	NA	NA
hsa-miR-6857-5p	URS000075A75C_9606	NA	NA	NA	NA
hsa-miR-6855-5p	URS000075AFD3_9606	NA	NA	NA	NA
hsa-miR-6839-3p	URS000075B5AB_9606	NA	NA	NA	NA

hsa-miR-4288	URS000075A3D3_9606	NA	NA	NA	NA
hsa-miR-4752	URS00002217EB_9606	NA	NA	NA	NA
hsa-miR-4474-3p	URS00003A57E5_9606	NA	NA	NA	NA
hsa-miR-6796-5p	URS0000759E61_9606	NA	NA	NA	NA
hsa-miR-6759-5p	URS000075A5C0_9606	NA	NA	NA	NA
hsa-miR-6818-5p	URS000075B57E_9606	NA	NA	NA	NA
hsa-miR-4677-5p	URS000032BDF4_9606	NA	NA	NA	NA
hsa-miR-302c-5p	URS000075DC15_9606	NA	NA	NA	NA
hsa-miR-3658	URS000075A6CE_9606	NA	NA	NA	NA
hsa-miR-4760-5p	URS00002126B8_9606	NA	NA	NA	NA
hsa-miR-498-5p	URS00004CCA62_9606	NA	NA	NA	NA
hsa-miR-3942-3p	URS00000672B5_9606	NA	NA	NA	NA
hsa-miR-587	URS000075D89C_9606	NA	NA	NA	NA
hsa-miR-5006-3p	URS000055E32C_9606	NA	NA	NA	NA
hsa-miR-670-3p	URS000075EF30_9606	NA	NA	NA	NA
hsa-miR-7152-5p	URS000075A801_9606	NA	NA	NA	NA
hsa-miR-7159-3p	URS000075E7E4_9606	NA	NA	NA	NA
hsa-miR-4482-3p	URS000075E237_9606	NA	NA	NA	NA
hsa-miR-4743-3p	URS000075B649_9606	NA	NA	NA	NA
hsa-miR-1304-5p	URS00000753AD_9606	NA	NA	NA	NA
hsa-miR-12131	URS0000D53350_9606	NA	NA	NA	NA
hsa-miR-133b	URS000032BD73_9606	NA	NA	NA	NA
hsa-miR-7856-5p	URS000075BCE8_9606	NA	NA	NA	NA
